# DFT-based machine-learning for the rational design of magnetocaloric high-entropy alloys

**DOI:** 10.1038/s41467-026-77123-w

**Published:** 2026-08-25

**Authors:** Zhe Cui, Carlos Romero-Muñiz, Jia Yan Law, Victorino Franco

**Affiliations:** Multidisciplinary Unit for Energy Science, Department of Condensed Matter Physics, University of Seville, Seville, Spain

**Keywords:** Phase transitions and critical phenomena, Theory and computation, Magnetic properties and materials

## Abstract

The vast compositional space of high-entropy alloys (HEA) holds promise for next-generation functional materials, yet its exploration is stifled by a combinatorial bottleneck: conventional first-principles accuracy is computationally prohibitive for complex disordered lattices, while black-box machine learning (ML) lacks physical interpretability. We overcome this by establishing a minimal-supercell principle, demonstrating that the magnetostructural behaviour is an emergent feature of local atomic environments rather than long-range configurations. This enables a physics-informed ML framework that inverts the conventional screening approach, transitioning from limited local identification to global design-space mapping, reducing computational time by 40 – 60% without compromising accuracy. Applying this framework to MM’X family uncovers a fundamental design dichotomy: mean electronic descriptors govern baseline phase stability, while dispersion descriptors, specifically the magnetic-moment dispersion, drive the critical transformation response. This analysis reveals an intrinsic stability-performance trade-off, where the disorder required to maximise the transformation driving force inevitably penalises thermodynamic stability. By quantifying this Pareto frontier, we propose a hierarchical tuning strategy that decouples phase transition temperature from hysteresis, providing a scalable paradigm to transform HEA exploration from serendipitous discovery into rational design.

## Abstract (web summary)

Using a DFT + ML workflow to predict magnetocaloric high-entropy alloy compositions that links macroscopic and microscopic properties, this manuscript highlights actionable knobs to fine-tune the desired properties of experimentally obtained alloys.

## Introduction

The global shift towards sustainable energy-efficient cooling solutions has heightened the search for advanced magnetic materials. Considering that refrigeration and air conditioning account for approximately 20% of global electricity consumption, substituting vapour-compression systems with solid-state magnetocaloric cooling (based on the magnetocaloric effect) offers a pathway to substantially decrease carbon emissions^1,2,3^. Several magnetocaloric material families, such as Gd-based systems, Heusler alloys, MM’X alloys and LaFe_13−*x*_Si_*x*_-based systems, have also attracted considerable interest^4^. However, many of the state-of-the-art magnetocaloric materials rely heavily on rare-earth (RE) elements (e.g. Gd, La, Dy, Ce, etc.), which suffer from severe supply chain volatility and high environmental extraction costs^5–7^. This challenge has shifted the frontier of materials science toward high-entropy alloys (HEAs), which not only offer a route towards RE-free magnetocaloric materials but also present potential benefits in terms of mechanical robustness and corrosion/oxidation resistance, thereby further enhancing their practical applicability. HEAs are a class of compositionally complex materials composed of multiple principal elements that stabilise novel solid-solution phases through high configurational entropy^8,9^. Since their inception, the field has evolved from simple equiatomic single-phase to complex 3rd generation HEA systems with intermetallic and/or multiphases, massively expanding the compositional space of discoverable properties^10–12^. By navigating this vast compositional space, HEAs offer the potential to design RE-free alternatives^7,13^ with precisely tuneable critical parameters, such as the thermomagnetic phase transition temperatures and isothermal entropy change, via the vast combinatorial possibilities of the cocktail effect^14–16^.

However, the immense potential of HEAs comes at the cost of a severe combinatorial bottleneck. The sheer array of possible element combinations and stoichiometries creates a design space so vast (a typical quinary HEA system can yield billions of stoichiometric combinations) that traditional *Edisonian* trial-and-error experimental approaches are statistically impractical^8,17,18^. While experimental verification remains essential for performance evaluation, it struggles to keep pace with the magnitude of multicomponent systems available^19,20^. Theoretical guidance is therefore essential, yet modelling magnetic disorder in HEAs presents a fundamental dilemma. First-principles methods, such as density functional theory (DFT), provide necessary insights into electronic, magnetic and thermodynamic behaviours, but their high computational cost limits the number of configurations that can be realistically assessed for HEAs^21,22^. Furthermore, standard DFT calculations rely on periodic boundary conditions, meaning they inherently struggle to accurately reflect the random distribution of substitutional atoms in these alloys. To model disordered, nearly random atomic distributions in HEAs within periodic boundary conditions, researchers usually turn to approximate methods, such as special quasirandom structures (SQS)^23–25^, the coherent potential approximation (CPA)^26–28^, virtual crystal approximation (VCA)^29,30^, as well as statistical averaging approaches^31,32^. However, a critical gap remains between these techniques: SQS provides atomic-level resolution but requires large, computationally expensive supercells to statistically mimic randomness, rendering them prohibitive for high-throughput screening. Conversely, mean-field methods like CPA are efficient but obliterate the local lattice distortions and chemical environments that often govern functional performance. This leads to a central question: can we dissociate computational cost from predictive accuracy in disordered systems while retaining the atomic-level resolution needed to grasp the underlying physics?

Machine learning (ML) has been proposed to navigate this dilemma^33,34^, yet current implementations often suffer from disjointed training strategies and their efficacy is strictly limited by the quality of their training data. Models based solely on standard DFT inherit its computational burden and biases^21,35^, while those grounded on experimental data, although more reflective of actual behaviour, often grapple with data scarcity, noise and uneven sampling bias^36,37^. This typically results in a loss in predictive reliability, especially in the high-entropy regime^38,39^, leaving vast regions of the potential design space unexplored. Furthermore, standard black-box ML approaches often provide predictions without physical insight, failing to reveal the underlying mechanisms (like magnetic moment variations) that actually drive performance. To realise the full potential of HEAs, we require a framework that not only accelerates discovery but also provides interpretable physical guidance rooted in first-principles accuracy.

**Fig. 1:**
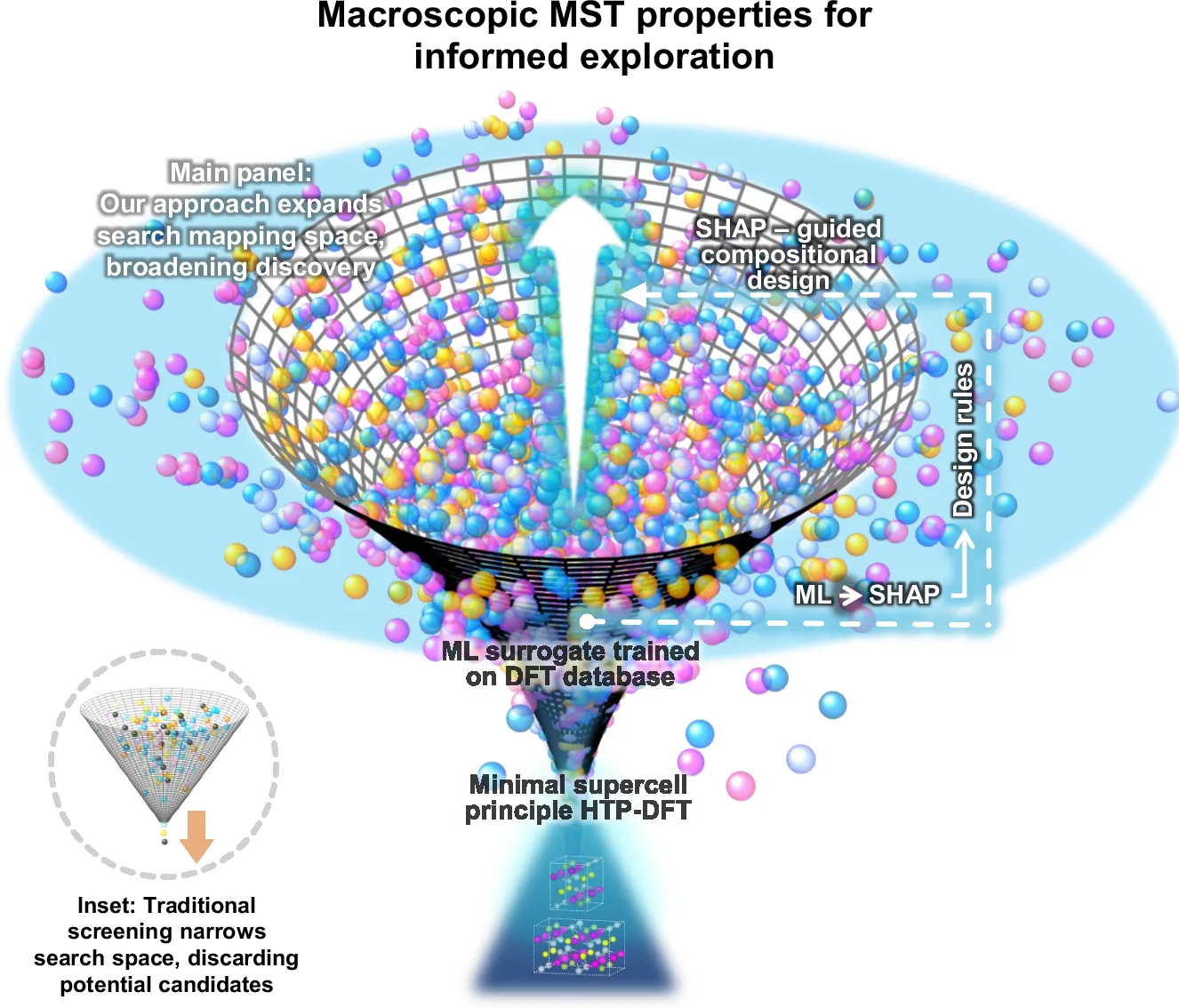
Inverted materials screening extends to global design-space mapping. Main panel: The workflow begins at the narrow base, where the minimal-supercell principle and high-throughput DFT (HTP-DFT) capture essential local atomic environments for a training dataset with first-principles accuracy. A physics-based machine learning (ML) surrogate projects local insights upward, expanding the search space to comprehensively map the vast high-entropy alloy (HEA) compositional landscape. Outcome: SHapley Additive exPlanations (SHAP) analysis decodes the expanded map to extract interpretable design rules, such as magnetic moment dispersion, guiding the rational discovery of optimised magnetocaloric materials with magnetostructural (MST) behaviour. Inset (bottom-left): Traditional screening methods inherently narrow the search space, prematurely discarding promising candidates.

In this work, we resolve this dichotomy by unifying a minimal-supercell principle with an interpretable DFT–ML framework. We posit that for many functional HEAs, macroscopic properties are not driven by long-range configurational randomness but emerge from specific local atomic motifs. Figure 1 illustrates this workflow, which effectively inverts the conventional screening paradigm. Instead of filtering down from a massive initial pool of candidates via a traditional screening funnel limited to a narrow slice of configurational space, as illustrated in the inset, our approach anchors the model on a concise foundation of minimal structures at the base. High-throughput DFT (HTP-DFT) calculations on these minimal supercells capture essential local atomic environments, generating a robust training dataset with first-principles accuracy and maximising information density at a low computational cost. This dataset feeds a physics-informed ML surrogate, acting as a scaling engine to break through the conventional computational ceiling. In this expanded regime, the model extrapolates to map the stability-performance Pareto frontier across the global design space, identifying high-performance regions missed by standard heuristic screening. Finally, SHapley Additive exPlanations (SHAP)^40,41^ analysis decodes this global map, extracting interpretable design rules, specifically the roles of *d*-electron and magnetic moment, to guide the rational design of optimised magnetocaloric high-entropy materials.

We validate this framework using the MM’X family of magnetocaloric alloys (where M, M’ are transition metal elements and X is a *p*-block element). This system serves as an ideal testbed because its functional performance, specifically the large magnetocaloric and multicaloric effect^42–45^, relies on strong magnetostructural coupling, a phenomenon highly sensitive to the very local lattice distortions that mean-field methods fail to capture. This family also hosts the first proven first-order thermomagnetic phase transition in HEA space^11,46^, defining the emerging class of 3^rd^ generation HEAs. Our results demonstrate a 40%–60% reduction in computational time while maintaining excellent agreement with experimental lattice parameters and magnetic phase transition temperatures. By projecting physical descriptors into property space, we establish a scalable, physically interpretable pathway for the rational accelerated design of functional high-entropy materials.

## Results

### Efficient DFT methodology for HEAs: supercell selection and atomic ordering

To resolve the combinatorial bottleneck inherent to HEA exploration, we first established the physical validity of the minimal-supercell principle. We benchmarked seven supercells across three compositional series, MnNiSi_1−*x*_Ge_*x*_, Mn_*x*_Fe_1−*x*_NiSi and MnNi_*x*_Fe_1−*x*_Si, in both hexagonal and orthorhombic crystal structures. For each series, compositions from *x* = 0 to 1 were sampled in steps of 0.125, using a family of compact supercells (1 × 1 × 1 (12 atoms), 2 × 1 × 1, 1 × 2 × 1, 1 × 1 × 2 (24 atoms), 2 × 2 × 1, 1 × 2 × 2, 2 × 1 × 2 (48 atoms)). Notably, the 1 × 1 × 1 supercell could not accommodate all compositions considered in this work, which motivated the use of larger supercells where required. The objective was to determine if compact, ordered cell sizes could reproduce the physical trends of larger, disordered systems. For each composition *x* and each {structure, supercell} combination, a single representative configuration (the lowest-energy ordered variant) was used for the primary analysis. To separate supercell-size effects from the dispersion associated with local atomic ordering, additional non-representative variants were analysed at intermediate compositions (*x* = 0.375, 0.5 and 0.625). All quantities are reported per atom, utilising consistent supercell scaling and identical geometric/magnetic preprocessing for both phases (see ‘Methods’ section).

**Fig. 2:**
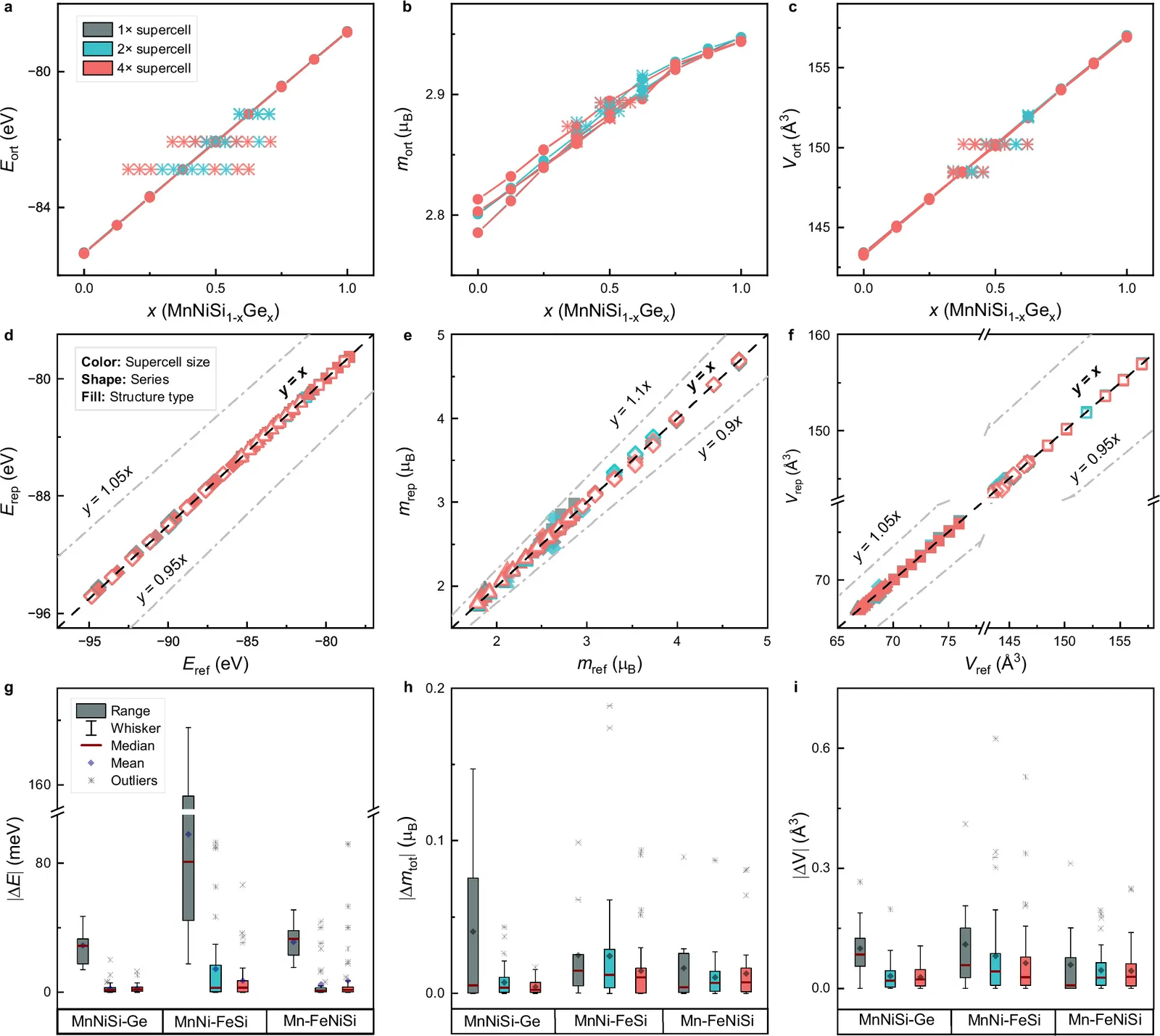
Composition–property relationships and supercell consistency. **a** Compositional dependence of energy, *E*, **b** magnetic moment, *m* and **c** volume, *V*, for the orthorhombic structure in MnNiSi_1-x_Ge_x_. Colours denote supercell size throughout; solid lines connect representative configurations, while symbols indicate non-representative configurations at the same *x*; a small horizontal displacement is added solely for visual separation. **d**–**f** Parity plots comparing representative values (${P}_{{{\mathrm{rep}}}}$) against the per-composition reference values (${P}_{{{\mathrm{ref}}}}$) for energy, magnetic moment and volume. The tight clustering along the 1:1 line confirms that minimal supercells introduce negligible systematic bias. Here, symbol shapes distinguish substitution series (squares, MnNiSi-Ge; diamonds, MnNi-FeSi; upward triangles, Mn-FeNiSi) and filled/open symbols distinguish hexagonal and orthorhombic structures. **g**–**i** Box plots summarising the deviation magnitude, $\left|{\rm\Delta }P\right|=\left|{P}_{{{\mathrm{rep}}}}-{P}_{{{\mathrm{ref}}}}\right|$, across all series, demonstrating that property predictions are robust against supercell choice. Boxes show interquartile range (IQR), centre line is the median, whiskers are 1.5 × IQR, and outliers are represented by scatter points. Exact *n* values and underlying data for each box-plot group are provided in Source Data. Detailed residual plots and full error-distribution analyses are provided in Supplementary Fig. 1.

As shown in Fig. 2a–c, the key physical descriptors, energy (*E*), magnetic moment (*m*) and volume (*V*), exhibit a remarkable insensitivity to supercell size (as observed at a fixed *x* from the compositional dependence of energy curves for MnNiSi_1−x_Ge_x_). The energy rankings remain consistent across all cell sizes, and deviations in magnetic moments are negligible (typically <1%). This robustness is observed across all three substitution series (MnNiSi_1−*x*_Ge_*x*_, MnNi_1−*x*_Fe_*x*_Si and Mn_1−*x*_Fe_*x*_NiSi) and persists in both orthorhombic and hexagonal phases (Supplementary Figs. 2–4). Occasional spread is observed near intermediate Mn–Fe substitutions, consistent with variations of Mn/Fe moments. *m* and *V* display a somewhat broader yet still acceptable spread. Notably, the MnNi_0.375_Fe_0.625_Si composition in the 2 × 1 × 2 hexagonal supercell (Supplementary Fig. 4d, f green line/points) stabilises in a ferrimagnetic configuration. This behaviour can occasionally arise in DFT calculations, resulting in *m* and *V* values that may not be appropriate for that specific point^32^. Therefore, we exclude these from the supercell-consistency analysis to ensure the reliability of our results. Together, these results confirm a critical hypothesis: macroscopic properties in these functional alloys are emergent features of local atomic motifs rather than long-range random disorder. Consequently, we establish that the smallest symmetry-compatible supercell (24 atoms, 2× supercell) is sufficient to capture the essential physics, providing a computationally efficient yet rigorous basis for global exploration.

To quantify this agreement, we compared the properties of the lowest-energy representative configuration (*P*_rep_) against the median reference values of the full supercell ensemble (*P*_ref_). The resulting parity plots in Fig. 2d–f display a tight clustering along the 1:1 diagonal (black dashed), indicating that the minimal cell introduces negligible systematic bias. Furthermore, the deviation magnitude, $\Delta P\left(x\right)={P}_{{{{\rm{rep}}}}}-{P}_{{{{\rm{ref}}}}}$, fluctuates narrowly around zero with weak compositional dependence, as observed in Fig. 2g–i. Detailed residual trends and full error-distribution analyses are provided in Supplementary Fig. 1. Pooling all series (Supplementary Table 2), the deviations remain very small: the median energy error ∣ΔE∣ is only 2.172 meV/f.u., while volume deviations $| \Delta V| /{V}_{{{{\rm{ref}}}}}$ are typically below 0.1%, and magnetic moment deviations $| \Delta m| /{m}_{{{{\rm{ref}}}}}$ remain at the sub-percent level. A Kruskal–Wallis test across supercells detects a difference for *E* (H=70.429, $p=3.34\times {10}^{-13}$) and a weak effect for *V* (H=12.669, p=0.049), while *m* shows no significant dependence (H=4.096, p=0.664). However, given that the median deviations and 95th-percentile intervals of ΔE and ΔV fall well within the typical precision limits of DFT calculations^47^ (approximately 1–2 meV/atom (3–6 meV/f.u. in this paper) for ΔE, and 0.38% for average volume deviation), these statistical differences have limited practical impact and do not indicate systematic bias.

As detailed in Supplementary Table 2, energy deviations between supercell sizes generally lie in the single digit meV/f.u. range. We observe that increasing the cell size from the primitive 1 × 1 × 1 to 2× supercells yields only a marginal reduction in energy dispersion, while expanding to 4× offers no tangible gain. This robustness is summarised in Fig. 2g–i (∣ΔP∣ magnitude faceted by series, colour = supercell, pooling all *x* and both structures, detailed supercell-resolved distributions provided in Supplementary Fig. 1d–f), which physically justifies our strategy: the minimal 24-atom supercell captures the essential physics with statistical equivalence to computationally expensive 48-atom ensembles, and simultaneously delivers a 40%–60% reduction in computational cost (as shown in Supplementary Tables 3–5). This efficiency gain arises from a synergistic combination of reduced self-consistent field (SCF) iteration counts and lower intrinsic per-step computational costs.

Expanding the supercell from primitive 1 × 1 × 1 to larger 2× and 4× configurations yields no statistically significant improvement in predicted trends, despite the substantial increase in computational cost. This observation reinforces the core tenet of the minimal-supercell principle: macroscopic functional properties are governed primarily by local atomic motifs rather than by long-range configurational disorder. Consequently, a streamlined DFT strategy based on the minimal representative cells provides a rigorous yet efficient proxy for the thermodynamic limit. To validate this approach against experimental reality, we benchmarked the predictions of our minimal-supercell framework (using a single lowest-energy ordered variant) against experimental data for diverse HEA-MM’X compositions reported in the literature and our own in-house work. As detailed in Table 1, the DFT-calculated lattice parameters and equilibrium volumes align with experimental measurements within typical DFT error margins. This concordance demonstrates that the minimal-supercell approximation offers a robust, experimentally anchored foundation for the high-throughput generation of our training dataset.

**Table 1:** Experimental benchmarking of the minimal-supercell strategy

Composition	Structure		*a* (Å)	*b* (Å)	*c* (Å)	*V* (Å³)	∆ (%)
Mn_0.625_Ni_0.625_Si_0.625_ Fe_0.375_Co_0.375_Ge_0.375_	ort	DFT	5.833	3.594	6.947	145.615	2.009
Mn_0.62_Ni_0.62_Si_0.62_ Fe_0.38_Co_0.38_Ge_0.38_^66^		exp.	5.777	3.686	6.978	148.600	
Mn_0.625_Ni_0.625_Si_0.625_ Fe_0.375_Co_0.375_Ge_0.375_	hex	DFT	3.985	-	5.097	70.126	3.007
Mn_0.62_Ni_0.62_Si_0.62_ Fe_0.38_Co_0.38_Ge_0.38_^66^		exp.	4.016	-	5.180	72.300	
Mn_0.75_Ni_0.75_Si_0.75_ Fe_0.5_Ge_0.25_	ort	DFT	5.844	3.565	6.987	145.565	2.502
Mn_0.75_Ni_0.75_Si_0.75_ Fe_0.5_Ge_0.25_^67^		exp.	5.842	3.660	6.978	149.300	
Mn_0.75_Ni_0.75_Si_0.75_ Fe_0.5_Ge_0.25_	hex	DFT	3.986	-	5.070	69.730	3.688
Mn_0.75_Ni_0.75_Si_0.75_ Fe_0.5_Ge_0.25_^67^		exp.	4.022	-	5.168	72.400	
Mn_0.75_Fe_0.25_NiGe_0.75_Si_0.25_	ort	DFT	5.944	3.620	7.033	151.343	2.359
Mn_0.74_Fe_0.26_NiGe_0.75_Si_0.25_^68^		exp.	5.935	3.710	7.041	155.000	
Mn_0.75_Fe_0.25_NiGe_0.75_Si_0.25_	hex	DFT	4.046	-	5.189	73.456	2.578
Mn_0.74_Fe_0.26_NiGe_0.75_Si_0.25_^68^		exp.	4.060	-	5.280	75.400	
Mn_0.625_Fe_0.75_Ni_0.625_ Si_0.5_Ge_0.5_	ort	DFT	5.902	3.543	7.098	148.436	1.218
Mn_0.67_Fe_0.665_Ni_0.665_ Si_0.5_Ge_0.5_^46^,^a^		exp.	5.812	3.682	7.023	150.266	
Mn_0.625_Fe_0.75_Ni_0.625_ Si_0.5_Ge_0.5_	hex	DFT	4.027	-	5.062	70.981	2.362
Mn_0.67_Fe_0.665_Ni_0.665_ Si_0.5_Ge_0.5_^46^,^a^		exp.	4.031	-	5.167	72.698	

Comparison of DFT-calculated lattice parameters (*a*, *b*, *c*) and unit-cell volumes (*V*) against experimental and literature values for HEA-MM’X, in both hexagonal (hex) and orthorhombic (ort) structures. DFT values correspond to fully relaxed 0 K calculations using the minimal-supercell approach; experimental values (exp.) are taken from refs. ^46,66–68^. Experimental values from this work are marked with ^a^. The relative volume difference $\Delta (\%)=100\times |{V}_{\exp .}-{V}_{{{{\rm{DFT}}}}}|/{V}_{\exp .}$ quantifies the predictive accuracy of the method.

### A physics-informed machine-learning surrogate for global design space mapping

**Fig. 3:**
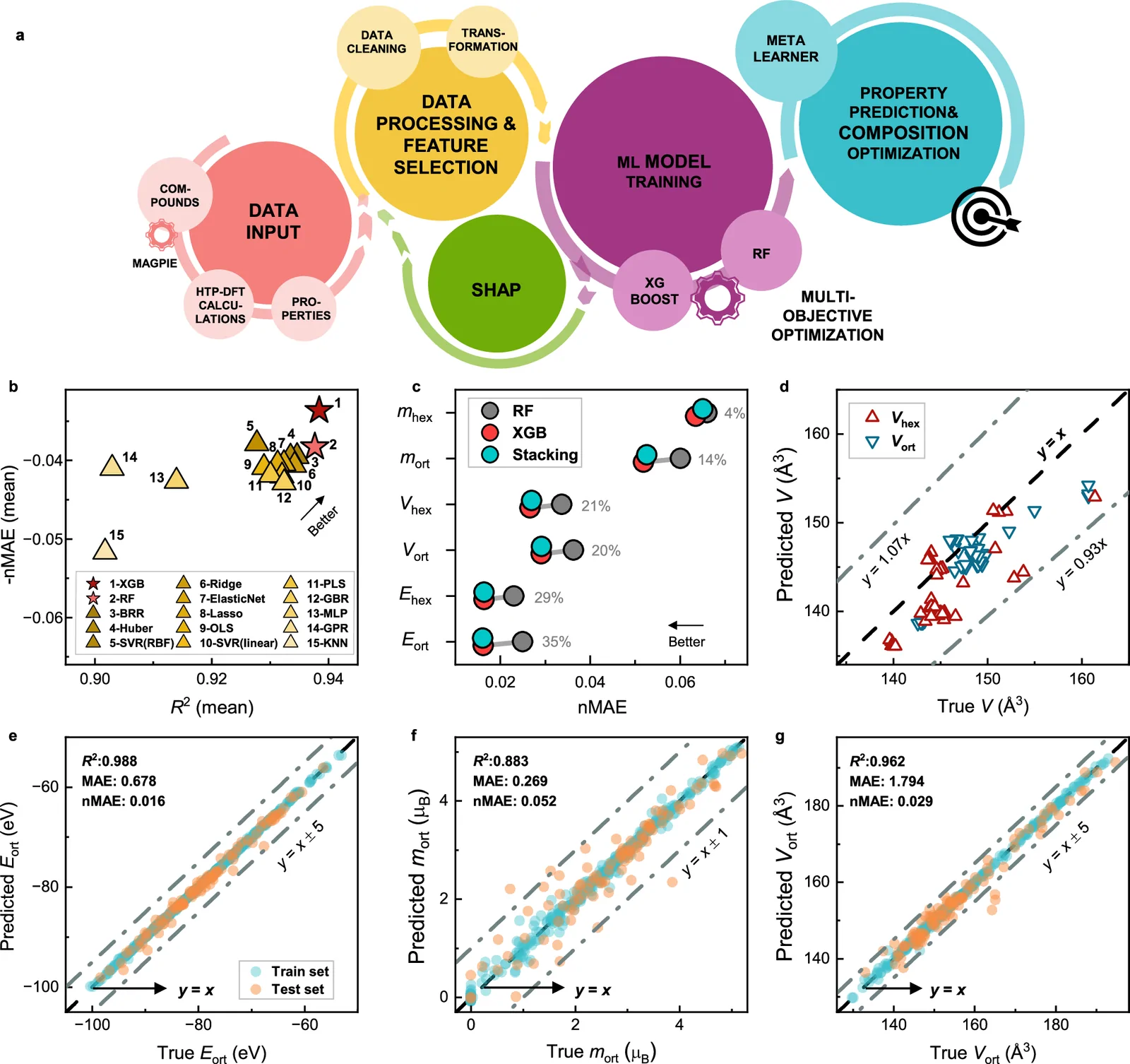
Machine-learning surrogate performance and validation. **a** Schematic diagram of the ML workflow linking the HTP-DFT dataset to the final multi-output surrogate model. **b** Model screening: trade-off between nMAE and mean *R*^2^ for various ML algorithms. It highlights XGB and RF as the best performers (star symbols). **c** Aligned comparison plot of test nMAE values for RF, XGB and stacking models across the six target properties (*E*, *V* and *m* for both structures). Lower nMAE indicates better predictive accuracy. The shift from RF to XGB shows the improvement achieved by the XGB surrogate, whereas the near-overlap between XGB and stacking indicates that the stacking ensemble provides no systematic additional gain over the XGB baseline. **d** Comparison between ML-predicted vs. experimental/literature unit-cell volumes for various hexagonal (hex) and orthorhombic (ort) MM’X. The solid black line (*y* = *x*) represents perfect agreement, and dashed lines indicate a ±7% error band. Experimental values are taken from in-house work and literature^11,66,68–81^. **e**–**g** Detailed parity plots between ML predictions and DFT calculations for ${E}_{{{{\rm{ort}}}}}$, ${m}_{{{{\rm{ort}}}}}$ and ${V}_{{{{\rm{ort}}}}}$, respectively. The tight grouping around *y* = *x* (black dashed line) demonstrates high accuracy.

With a high-throughput dataset of physics-based training data secured, we developed a multi-output ML surrogate to project these local insights across the vast compositional landscape. The workflow, summarised in Fig. 3a, links experimentally anchored DFT inputs to a gradient-boosted decision tree architecture. We utilised a curated dataset of 470 MM’X compositions derived from our HTP-DFT study^48^. Following an 80/20 split, 376 compositions were allocated for training and cross-validation, with 94 reserved as a held-out test set. To ensure model interpretability and reduce overfitting, we applied a rigorous feature selection pipeline, condensing 132 composition-only Magpie descriptors^49^ into a compact set of 29 non-redundant descriptors (retaining ~22% of the original feature space). This selection isolates the descriptors with the strongest predictive signals for *V*, *m* and *E*^50^.

Leveraging this condensed feature set, we benchmarked candidate algorithms using the *Scikit-learn* library^51^ wrapped in a MultiOutputRegressor to jointly predict six targets. Model robustness was assessed via 10-fold cross-validation on the training split (see Supplementary Table 6). For each model and target, we computed the coefficient of determination (*R*^2^), mean absolute error (MAE), root mean squared error (RMSE) and normalised mean absolute error (nMAE); aggregate performance is summarised by the mean nMAE and mean *R*^2^ across all outputs.

As shown in Fig. 3b, linear baselines already achieve high correlation (*R*^2^ > 0.93, mean nMAE ≈ 0.04), confirming the intrinsic physical relevance of our selected descriptors. However, tree-based ensembles and kernel methods demonstrate a superior capacity to capture complex non-linear magnetostructural coupling. In particular, Random Forest (RF)^52^ and XGBoost (XGB)^53^ emerge as the efficiency leaders, achieving mean nMAE ≈ 0.038 and 0.034, respectively. Consequently, we selected these architectures for multi-objective hyperparameter optimisation.

Final validation on the independent held-out test set confirms the superiority of the XGBoost surrogate (see Supplementary Table 7). While RF serves as a strong baseline (*R*^2^ ≈ 0.94–0.98, test nMAE ≈ 0.02–0.04), XGB systematically achieves higher accuracy across all targets (*R*^2^ ≈ 0.96–0.99, nMAE ≈ 0.02–0.03). Most notably, the model demonstrates remarkable robustness in predicting the magnetic moment, a property traditionally difficult to capture due to sensitive electronic interactions. Even for these challenging targets, the surrogate maintains high predictive accuracy (*R*^2^ ≈ 0.80–0.88, nMAE ≈ 0.05–0.06), validating its capability to capture complex magnetic transitions in unseen regions of the compositional space. A stacking ensemble^54^ was also tested (See Fig. 3c), but it did not yield meaningful improvement over XGB, which was therefore retained for all subsequent analyses.

The predictive power of this final model is detailed in Fig. 3e, f and Supplementary Fig. 6. For volumes and total energies, the parity plots reveal a tight clustering around the *y* = *x* line, consistent with the high test-set scores reported in Supplementary Table 7 (*R*^2^ ≈ 0.96–0.99, nMAE ≈ 0.02–0.03). While the magnetic moments of orthorhombic and hexagonal phases exhibit slightly larger scattering, the model successfully captures the critical trends required to evaluate magnetocaloric performance. This confirms that the surrogate is sufficiently accurate to discern the subtle relative stabilities between competing phases, a requisite for identifying magnetostructural transformations.

For validating the predictive capacity of the model with physical reality, we employed a dual-benchmarking strategy, comparing the prediction of the surrogate against a consolidated library of experimental data (literature and in-house) and computational enumerations. Figure 3d compares XGB-predicted equilibrium volumes against experimentally characterised values, using only nominal stoichiometries as input. Crucially, the model reproduces not only the absolute magnitudes but also the relative volume trends in response to compositional tuning across diverse MM’X families. As detailed in Table 2, the surrogate captures the experimentally reported lattice parameters (as well as in-house work) with high accuracy, despite a slight systematic underestimation of the unit-cell volume. This likely reflects the difference between the DFT-relaxed 0 K structures used for training and the finite-temperature conditions of experimental measurements. By correctly identifying both the direction and magnitude of unit-cell variations, the model demonstrates a robust validity in a regime directly relevant to magnetocaloric design.

**Table 2:** External validation against experimental and literature benchmarks

Composition	Phase	${V}_{{\mbox{lit}}}$ (Å³)	${V}_{{\mbox{XGB}}}$ (Å³)	Δ (%)
Mn_0.45_Fe_0.55_NiGe_0.30_Si_0.70_^68^	hex	71.7	69.5	3.138
	ort	146.5	144.6	1.313
Mn_0.74_Fe_0.26_NiGe_0.75_Si_0.25_^68^^,^	hex	75.4	73.5	2.464
	ort	155	151.4	2.352
Ni_0.66_Mn_0.68_Si_0.55_Fe_0.66_Ge_0.45_^69^,^a^	hex	72.0	69.7	3.159
	ort	148.3	148.0	0.187
MnNi_0.48_Si_0.48_Fe_0.52_Ge_0.52_^70^	hex	76.4	71.9	5.905
Mn_0.65_Ni_0.65_Si_0.65_Fe_0.7_Ge_0.35_^66^	hex	72.7	69.5	4.323
	ort	149.1	148.3	0.534
Mn_0.62_Ni_0.62_Si_0.62_Fe_0.38_Co_0.38_Ge_0.38_^71^	hex	72.0	70.7	1.821
	ort	148.6	146.2	1.641
Mn_0.68_Ni_0.68_Co_0.64_Si_0.68_Ge_0.32_^72^	hex	72.2	70.3	2.676
Mn_0.69_Ni_0.69_Co_0.62_Si_0.69_Ge_0.31_^72^	ort	147.5	145.1	1.641
Mn_0.85_Co_0.3_Ni_0.85_Si_0.83_Ga_0.17_^73^	hex	71.8	72.9	1.546
Mn_0.85_Co_0.3_Ni_0.85_Si_0.84_Ga_0.16_^73^	ort	146.9	146.8	0.090
Mn_0.85_Ni_0.85_Fe_0.30_Si_0.85_Ga_0.15_^74^	hex	72.3	72.1	0.328
Mn_0.86_Ni_0.86_Fe_0.28_Si_0.86_Ga_0.14_^74^	ort	148.7	147.2	1.022
Mn_0.6_Ni_0.6_Si_0.6_Fe_0.4_Co_0.4_Ge_0.4_^75^	hex	72.2	70.73	2.078
Mn_0.84_Ni_0.84_Si_0.84_Co_0.32_Ga_0.16_^76^	hex	72.1	72.4	0.544
	ort	146.6	146.8	0.168

Comparison between literature DFT equilibrium volumes and XGB-predicted volumes for selected HEA-MMX compositions. For each alloy and phase, the table lists the published volume ${V}_{{\mbox{lit}}}$, the XGB prediction ${V}_{{\mbox{XGB}}}$, their difference $\Delta={100\times |V}_{{\mbox{XGB}}}-{V}_{{\mbox{lit}}}|/{V}_{{\mbox{lit}}}$. Literature values are taken from independent studies^66,68–76^. Values from this work are marked with ^a^. This demonstrates the model’s transferability to unseen experimental systems.

As a secondary benchmark, we overlaid the XGB predictions (grey dashed lines) onto our internal dataset (coloured symbols; composition versus volume *V*, magnetic moment *m* and energy *E* as introduced in previous section) for MnNiSi_1−*x*_Ge_*x*_, Mn_*x*_Fe_1−*x*_NiSi and MnNi_*x*_Fe_1−*x*_Si series (with *x* = 0, 0.125, 0.25, 0.375, 0.5, 0.625, 0.75, 0.875, 1; see Supplementary Figs. 2–4). This dataset contains dense supercell enumerations that test the interpolation capabilities of the model. In nearly all instances, the ML-predicted curves closely follow the DFT results, falling well within the statistical variance of supercell calculations. This dual validation, against independent experiments and dense theoretical grids, confirms that the surrogate is not merely fitting numerical inputs but has successfully captured the underlying physics of magnetostructural behaviour. This establishes our XGB model as a robust engine for the global design space mapping and subsequent SHAP analysis discussed next.

### Elucidating the physicochemical drivers of magnetocaloric performance

To decouple the complex factors governing magnetocaloric performance, we analysed the XGB surrogate using SHAP. We restricted the analysis to the 22 most relevant Magpie descriptors^49^ retained by the model, as summarised in Table 3. This compact, physically meaningful subset was distilled from the original 132 composition-only descriptors (Supplementary Table 1) and captures three essential aspects of the alloys: (i) steric constraints defined by the atomic weights and covalent radii, (ii) electronic configuration governing bonding strength and stability via total and unfilled valence states and (iii) chemical and magnetic heterogeneity captured by the Mendeleev-number and, crucially, the magnetic-moment descriptors, which serve as the primary indicator of magnetostructural coupling. These are complemented by cohesive energy descriptors (melting temperature descriptors) and *s*-valence electrons. To extract clear design principles, we focused on six phase-resolved targets relevant to the magnetocaloric effect: the orthorhombic and hexagonal phase formation energies (${E}_{f}^{{{{\rm{ort}}}}}$ and ${E}_{f}^{{{{\rm{hex}}}}}$), the orthorhombic- and hexagonal-phase magnetic moments (${m}_{{{{\rm{ort}}}}}$ and ${m}_{{{{\rm{hex}}}}}$), the structural energy difference $\Delta {E}_{{str}}$ and the relative volume change $\Delta {V}_{{{{\rm{ratio}}}}}$. These targets were chosen because they capture complementary aspects of the magnetocaloric screening problem, namely low- and high-temperature phase stability, phase-resolved magnetic functionality, phase competition and transformation-related structural response. In particular, $\Delta {E}_{{str}}$ is treated here as a continuous descriptor of the energetic competition between the low-temperature orthorhombic phase and the high-temperature hexagonal phase, providing a basis for estimating or rationalising the martensitic transition temperature^55,56^, rather than as a strict ground-state classification label. The inclusion of both ${m}_{{{{\rm{ort}}}}}$ and ${m}_{{{{\rm{hex}}}}}$ enables a phase-resolved analysis of magnetic behaviour across the magnetostructural transition. For the MM’X systems considered here, the desired magnetocaloric response is associated with a transition from a ferromagnetic orthorhombic phase to a weakly magnetic or paramagnetic hexagonal phase; therefore, ${m}_{{{{\rm{ort}}}}}$ is particularly important for describing the ferromagnetic contribution to the magnetisation change, while ${m}_{{{{\rm{hex}}}}}$ provides a reference for the residual magnetism of the high-temperature phase.

**Table 3:** Physicochemical descriptors retained in the final surrogate

Physical category	Physical symbol	Description	Physical interpretation
Electronic/chemical configuration	${{MN}}_{\max }$	maximum Mendeleev number	Chemical boundary of the alloy system
	${{MN}}_{{{{\rm{range}}}}}$	Mendeleev number range	Breadth of chemical diversity
	${{MN}}_{{{\mathrm{ad}}}}$	Average deviation of Mendeleev Number	Chemical dispersion (Driver of ${m}_{{{{\rm{ort}}}}}$)
	${{MN}}_{{{{\rm{mode}}}}}$	mode Mendeleev Number	Most frequent chemical species
	${\bar{n}}_{s}$	mean *s* valence electrons	*s*-orbital filling baseline
	${n}_{d,{{\mathrm{ad}}}}$	average deviation of *d* valence electrons	*d*-orbital filling disorder (*d*-band)
	${n}_{v,{{\mathrm{ad}}}}$	average deviation of total valence electrons	Global valence electrons variance
	${u}_{d,{{{\rm{ad}}}}}$	average deviation of unfilled *d* states	*d*-band hole dispersion
	${u}_{{{{\rm{T}}}},\min }$	minimum total unfilled states	Lower bound of total unfilled states (Secondary hysteresis control)
	${\bar{u}}_{{{{\rm{T}}}}}$	mean total unfilled states	Baseline of total unfilled states (Driver of Stability & ${m}_{{{{\rm{hex}}}}}$)
	${u}_{{{{\rm{T}}}},{ad}}$	average deviation of total unfilled states	Dispersion of total unfilled states (hysteresis-selective knob)
	${u}_{{{{\rm{T}}}},{{{\rm{mode}}}}}$	mode total unfilled states	Dominant unfilled states configuration
Structural/steric	${\bar{A}}_{w}$	mean atomic weight	Mass baseline
	${A}_{w,{{\mathrm{ad}}}}$	average deviation of atomic weight	Mass dispersion
	${r}_{{{\mathrm{cov}}},{{{\rm{range}}}}}$	covalent radius range	Steric size limit spread
	${\bar{r}}_{{{\mathrm{cov}}}}$	mean covalent radius	Average atomic packing size
	${r}_{{{\mathrm{cov}}},{{{\rm{ad}}}}}$	average deviation of covalent radius	Atomic size distortion
	${r}_{{{\mathrm{cov}}},{{{\rm{mode}}}}}$	mode covalent radius	Dominant atomic size
Thermodynamic	${T}_{{{{\rm{melt}}}},{{{\rm{range}}}}}$	melting temperature range	Cohesive energy spread
	${\bar{T}}_{{{{\rm{melt}}}}}$	mean melting temperature	Cohesive energy baseline
	${T}_{{{{\rm{melt}}}},{{{\rm{mode}}}}}$	mode melting temperature	Dominant cohesive species
Magnetic	${\sigma }_{{{{\rm{MST}}}}}$	average deviation of magnetic moment (per atom, 0 K)	Magnetostructural coupling (The trade-off Driver)

The 22 features selected from the original set of 132 Magpie descriptors are grouped by physical category and mapped to the symbolic notation used throughout the text. To facilitate physical interpretation, descriptors are classified into two types: mean descriptor ($\bar{x}$), representing the compositional baseline and dispersion descriptor (${x}_{{{\mathrm{ad}}}}$), representing the local compositional disorder (calculated as the average absolute deviation). For magnetic properties, m denotes the magnetic moment, with ${\sigma }_{{{{\rm{MST}}}}}$ (F_21_5_) serving as the critical descriptor for magnetostructural coupling.

**Fig. 4:**
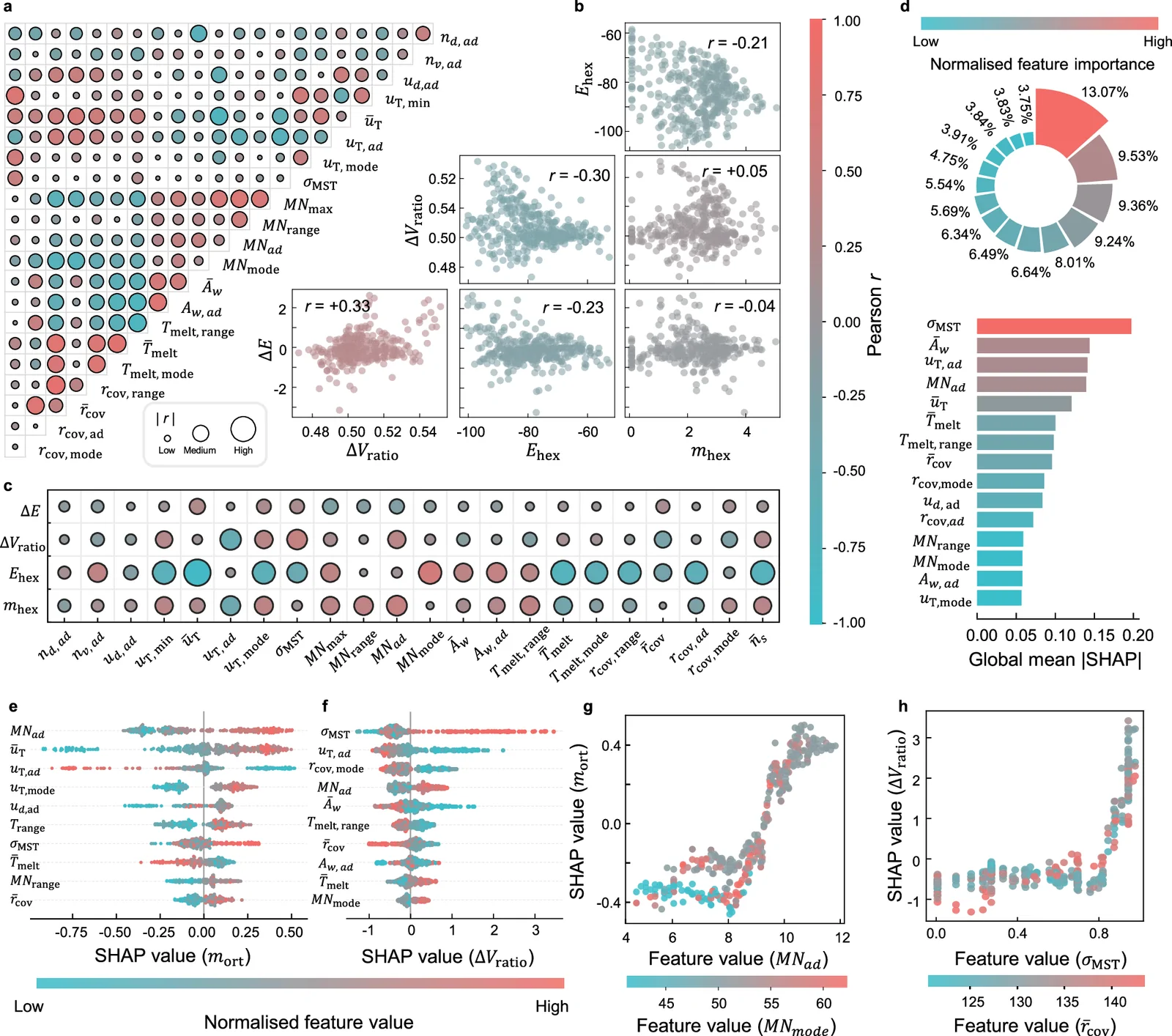
SHAP interpretation of the XGB surrogate. Pearson-correlation matrix between the selected Magpie descriptors and the key magnetocaloric proxies: **a** feature–feature correlations, **b** proxy–proxy correlations and **c** correlations between features and proxies. **d** Global SHAP feature importance averaged over six key targets (${E}_{f}^{{{{\rm{ort}}}}},\,{{E}_{f}^{{{{\rm{hex}}}}},{m}_{{{{\rm{ort}}}}},{m}}_{{{{\rm{hex}}}}},\,\Delta {E}_{{str}},\,\Delta {V}_{{{{\rm{ratio}}}}}$), (Upper: normalised feature-importance share (%). Lower: global mean |SHAP| values used to rank features), identifying atomic mass, covalent radius and unfilled states as dominant drivers. Colours in d indicate normalised feature importance. **e**, **f** SHAP summary plots for ${m}_{{{{\rm{ort}}}}}$ and $\Delta {V}_{{{{\rm{ratio}}}}}$, ranking features by their impact. Dot colours indicate min–max normalised feature values within each descriptor, because the descriptors have different units and numerical ranges. **g**, **h**, SHAP dependence plots illustrating the non-linear impact of the most influential descriptors on ${m}_{{{{\rm{ort}}}}}$ and $\Delta {V}_{{{{\rm{ratio}}}}}$, separate numerical colour bars indicate the values of the most corresponding interacting descriptors. Feature codes follow Table 3.

The global hierarchy of these drivers is summarised in Fig. 4. The correlation matrix in Fig. 4a–c provides a structural map of the dataset, highlighting expected physical covariances among the descriptors in Fig. 4a, while quantifying the overlap between the magnetocaloric proxies themselves in Fig. 4b. Crucially, the cross-correlations between descriptors and targets are weak (Fig. 4c, Pearson *r* ~ 0), confirming that the magnetocaloric responses arise from complex, non-linear physicochemical interactions rather than simple linear trends. The global SHAP feature importance plot in Fig. 4d cuts through this complexity by providing a global ranking of the descriptors across the six key targets, thereby highlighting which classes of features dominate the overall model response. The analysis reveals that the overall physics is governed by a fundamental competition between steric packing (mass/radius) and electronic structure (bonding/magnetism). The mass- and size-related descriptors, such as the mean atomic weight, melting temperature and covalent radius (e.g. ${\bar{A}}_{w}$, ${A}_{w,{ad}}$, ${T}_{{{{\rm{melt}}}},{{{\rm{range}}}}}$, ${\bar{T}}_{{{{\rm{melt}}}}}$, ${\bar{r}}_{{{\mathrm{cov}}}}$, ${r}_{{{\mathrm{cov}}},{{{\rm{range}}}}}$, ${r}_{{{\mathrm{cov}}},{{{\rm{mode}}}}}$), govern the overall lattice size and packing. The unfilled electronic states, especially the total unfilled states (${\bar{u}}_{{{{\rm{T}}}}}$, ${A}_{w,{{\mathrm{ad}}}}$, ${u}_{{{{\rm{T}}}},{{{\rm{mode}}}}}$), capture critical changes in bonding strength and electronic structure. The per-atom magnetic moments and Mendeleev numbers (${\sigma }_{{{{\rm{MST}}}}}$, ${{MN}}_{{{{\rm{range}}}}}$, ${u}_{{{{\rm{T}}}},{{\mathrm{ad}}}}$ and ${{MN}}_{{{{\rm{mode}}}}}$) quantify the degree of local magnetic and chemical variation.

To examine this global picture in more detail, the SHAP results can be interpreted at two complementary levels: the summary plots identify the most significant descriptors for each target, while the corresponding dependence plots reveal how these descriptors impact predictions. In this context, Fig. 4e, g describe the behaviour of ${m}_{{{{\rm{ort}}}}}$, whereas Fig. 4f, h emphasise the related trends for $\Delta {V}_{{{{\rm{ratio}}}}}$. This detailed analysis uncovers a refined design dichotomy: baseline-like phase-resolved properties are characterised by substantial mean-descriptor contributions and smooth, continuous descriptor responses, whereas magnetostructural transformation drivers exhibit stronger dispersion-controlled and threshold-like behaviour.

For instance, the formation-energy targets ${E}_{f}^{{{{\rm{ort}}}}}$ and ${E}_{f}^{{{{\rm{hex}}}}}$ are both dominated by mass- and size-related mean descriptors, such as the mean atomic weight (${\bar{A}}_{w}$) and mean covalent radius (${\bar{A}}_{{{\mathrm{cov}}}}$) (Supplementary Fig. 7a, b), and show highly consistent response trends (Supplementary Fig. 8a, b). Similarly, the hexagonal-phase magnetic moment ${m}_{{{{\rm{hex}}}}}$ is governed primarily by the baseline of unfilled total states (${\bar{u}}_{{{{\rm{T}}}}}$) (Supplementary Fig. 7c), with a largely smooth, nearly monotonic response shown in Supplementary Fig. 8c. Although ${m}_{{{{\rm{ort}}}}}$ is ranked highest with respect to the chemical dispersion (${{MN}}_{{ad}}$) (Fig. 4e), we classify it together with these baseline-like magnetic properties because ${\bar{u}}_{{{{\rm{T}}}}}$ still provides substantial contributions, and also the SHAP dependence to ${{MN}}_{{ad}}$ remains largely smooth and nearly monotonic (Fig. 4g). This suggests that ${m}_{{{{\rm{ort}}}}}$ behaves as a continuously tunable ferromagnetic baseline rather than a threshold-like transformation driver.

In contrast, the magnetostructural transformation drivers, specifically $\Delta {V}_{{{{\rm{ratio}}}}}$ and $\Delta {E}_{{{{\rm{str}}}}}$, are dominantly controlled by dispersion descriptors. The SHAP analysis reveals that $\Delta {V}_{{{{\rm{ratio}}}}}$ is governed principally by the magnetic-moment dispersion, ${\sigma }_{{{{\rm{MST}}}}}$, as shown in Fig. 4f. As illustrated in Fig. 4h, this dependence is distinctly non-linear: magnetic-moment dispersion acts as a gating mechanism. Below a critical threshold (≈0.8 in this dataset), its contribution to $\Delta {V}_{{{{\rm{ratio}}}}}$ is suppressive; beyond this point, the contribution rapidly shifts to a strong positive enhancement. Notably, ${\sigma }_{{{{\rm{MST}}}}}$ is also the dominant driver of $\Delta {E}_{{{{\rm{str}}}}}$, but with an opposing sign trend (Supplementary Figs. 7d and 8d). This indicates that the same dispersion knob acts in opposite directions with respect to thermodynamic stability and the hysteresis-related response (structural phase transition). This implies a critical design rule: while the stability of a magnetocaloric alloy depends on its average composition, its performance is governed by the variance of its local atomic environments.

### From SHAP to actionable knobs: a composition-tuning map

To translate these physical insights into a practical design strategy, we used the six-target, phase-resolved framework defined above, which includes both the orthorhombic and hexagonal phase formation energies (${E}_{f}^{{{{\rm{ort}}}}}$ and ${E}_{f}^{{{{\rm{hex}}}}}$) and their magnetic moments (${m}_{{{{\rm{ort}}}}}$ and ${m}_{{{{\rm{hex}}}}}$), together with the structural energy difference $\Delta {E}_{{{{\rm{str}}}}}$, and the relative volume change $\Delta {V}_{{{{\rm{ratio}}}}}$. Note that: since the orthorhombic and hexagonal phase formation energies, ${E}_{f}^{{{{\rm{ort}}}}}$ and ${E}_{f}^{{{{\rm{hex}}}}}$, share highly consistent drivers and response trends, we report the general formation-energy behaviour ${E}_{f}$ (using only ${E}_{f}^{{{{\rm{hex}}}}}$ as the representative stability metric).

**Fig. 5:**
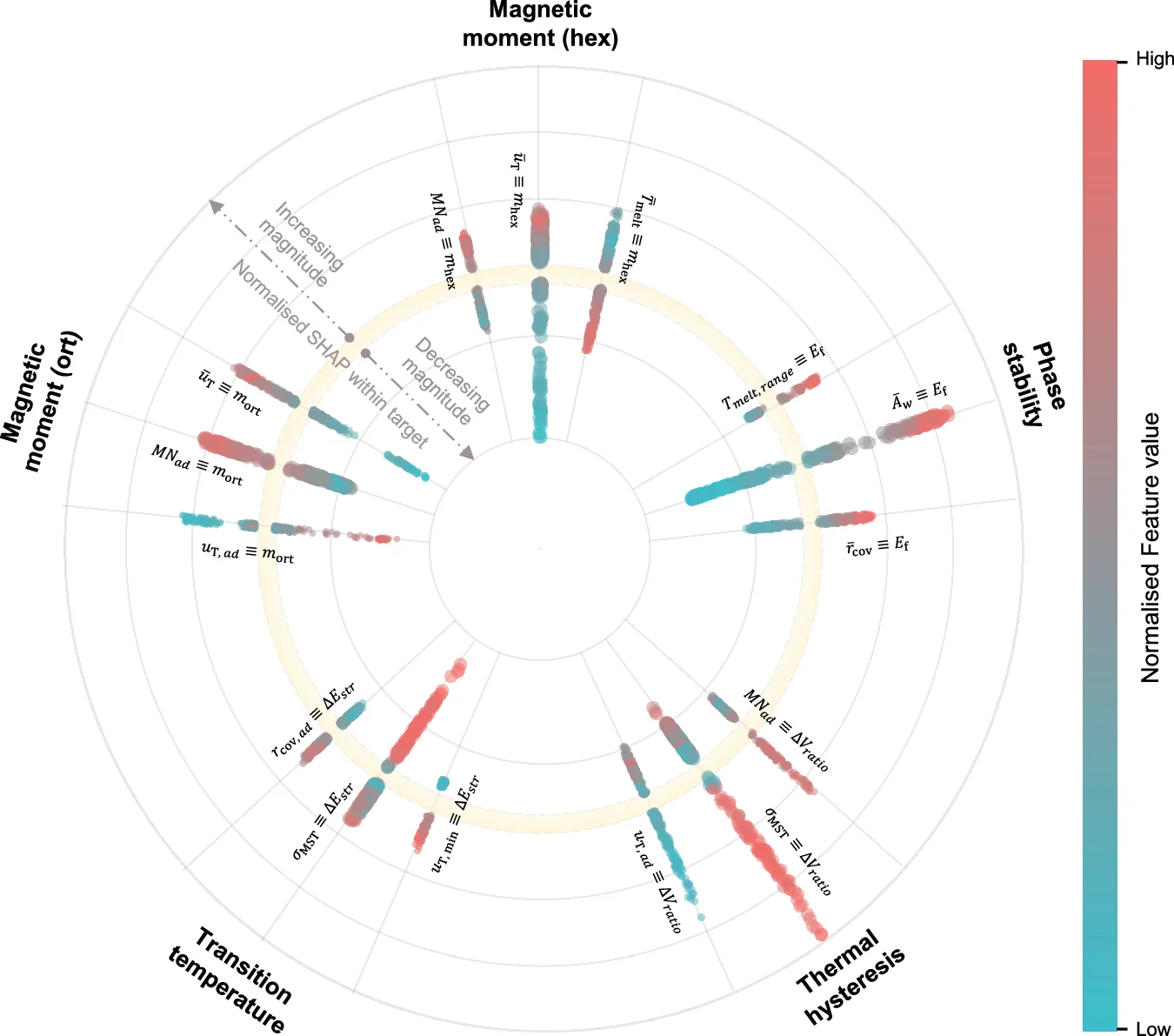
The stability-performance trade-off driven by synergistic disorder. Radial guide map connecting magnetocaloric-relevant performance metrics (outer labels) to the most actionable composition descriptors learned by the SHAP analysis. Each spoke corresponds to one experimentally relevant objective (e.g. magnetic moment of hex/ort phase, phase stability, transition temperature and thermal hysteresis). For each objective, the top-ranked descriptors are plotted as three jittered point clouds: the primary descriptor is centred on the spoke, while the descriptors (right: second; left: third) are shown with small angular offsets. Each dot represents one composition in the training set; the radial coordinate encodes the signed, target-normalised SHAP value, normalised separately within each target property (distance from the zero ring indicates contribution magnitude; inner/outer sides correspond to negative/positive contributions). Because SHAP magnitudes differ between target properties, radial positions should be interpreted as relative feature importance within each target rather than as a common absolute SHAP scale across all targets. Marker colour indicates the descriptor value (low to high, normalised per descriptor).

Figure 5 distils the SHAP interpretation into a radial guide map, visualising how specific descriptors serve as experimental levers for magnetocaloric optimisation. For the phase-resolved moments, the analysis reveals a distinct inversion in the hierarchy between ${\bar{u}}_{{{{\rm{T}}}}}$ and the chemical dispersion (${{MN}}_{{ad}}$). These two features jointly control both ${m}_{{{{\rm{hex}}}}}$ and ${m}_{{{{\rm{ort}}}}}$, but with a clear role swap in their relative importance across phases. Specifically, ${m}_{{{{\rm{hex}}}}}$ is dominated by the mean electronic descriptor ${\bar{u}}_{{{{\rm{T}}}}}$ (Top-1 predictor), with the dispersion descriptor ${{MN}}_{{{\mathrm{ad}}}}$ playing only a secondary role. In contrast, ${m}_{{{{\rm{ort}}}}}$ is dominated by ${{MN}}_{{{\mathrm{ad}}}}$ (Top-1 predictor), while ${\bar{u}}_{{{{\rm{T}}}}}$ becomes secondary. Importantly, the SHAP signs indicate a consistent *direction* of effect: both descriptors act with the same sign on ${m}_{{{{\rm{hex}}}}}$ and ${m}_{{{{\rm{ort}}}}}$, but their dominance swaps between phases. This implies a design rule: composition tuning can selectively target the magnetism of a specific phase by prioritising the corresponding top-ranked descriptor. One can tune the mean descriptor to shift the hexagonal baseline or tune the dispersion descriptor to manipulate the orthorhombic response, rather than relying on a single universal control parameter.

Crucially, magnetic moment dispersion (${\sigma }_{{{{\rm{MST}}}}}$) emerges as the primary driver for both the transformation driving force ΔE and the hysteresis-related $\Delta {V}_{{{{\rm{ratio}}}}}$, but with opposing consequences. In this context, ${\sigma }_{{{{\rm{MST}}}}}$ acts as a central dispersion guide; shifting it to enhance $\Delta {E}_{{{{\rm{str}}}}}$ tends to also decrease $\Delta {V}_{{{{\rm{ratio}}}}}$, thereby serving as the physical source of the trade-off between transformation sharpness and reversibility. These design directions are intrinsically antagonistic: $\Delta {E}_{{{{\rm{str}}}}}$ must be sufficiently large to ensure a robust driving force, yet not so large that it pushes the transition temperature to undesired impractical ranges, whereas $\Delta {V}_{{{{\rm{ratio}}}}}$ should be minimised to suppress hysteresis. To navigate this, SHAP identifies a second, hysteresis-selective handle: the electronic descriptor dispersion (specifically the dispersion of total unfilled states, ${u}_{{{{\rm{T}}}},{{\mathrm{ad}}}}$). Lower values of this descriptor are symmetrically associated with increased $\Delta {V}_{{{{\rm{ratio}}}}}$ (lowered reversibility), offering a secondary axis to decouple the trade-off.

From this analysis, a specific tuning protocol based on two rules emerges:

- Step 1: Set the transformation-hysteresis balance.
  Use ${\sigma }_{{{{\rm{MST}}}}}$ as the primary lever to establish the necessary driving force ($\Delta {E}_{{{{\rm{str}}}}}$). Since ${\sigma }_{{{{\rm{MST}}}}}$ acts as a central dispersion guide, increasing it also strengthens the magnetostructural coupling, but inevitably increases $\Delta {V}_{{{{\rm{ratio}}}}}$. Therefore, ${\sigma }_{{{{\rm{MST}}}}}$ should be tuned to the minimum level required to trigger the structural transformation, thereby avoiding excessive lattice distortion.
- Step 2: Mitigate hysteresis independently.
  Once the transformation is established, tune the electronic descriptor ${u}_{{{{\rm{T}}}},{{\mathrm{ad}}}}$. Our model indicates that their lower values are systematically associated with reduced hysteresis proxies ($\Delta {V}_{{{{\rm{ratio}}}}}$) without significantly weakening the driving force established in Step 1. Additionally, checking the lower bound of unfilled states (${u}_{{{{\rm{T}}}},\min }$) offers a secondary control to further suppress volume expansion without working against the intended $\Delta {E}_{{{{\rm{str}}}}}$ trend.

By following this sequence, researchers can utilise ${\sigma }_{{{{\rm{MST}}}}}$ to set the coarse performance baseline and then refine reversibility via ${u}_{{{{\rm{T}}}},{ad}}$ and ${u}_{{{{\rm{T}}}},\min }$, effectively decoupling the control of the transition temperature from the hysteresis loss. By selecting the specific spoke of the target property in Fig. 5, researchers can identify the top-ranked, sign-resolved descriptors as actionable knobs, transforming composition refinement from empirical trial-and-error into descriptor-driven directional optimisation.

## Discussion

This work addresses a central dilemma in the design of high-entropy and compositionally complex alloys: bridging the gap between rigorous first-principles accuracy with physically meaningful information, and the demand for computational efficiency when exploring the vast scale of configurational space. We have developed a physics-informed DFT-ML framework that inverts the conventional screening paradigm, moving from limited local identification to global design space mapping. By anchoring the search on the minimal-supercell principle, we demonstrate that streamlined inputs are sufficient to capture essential physicochemical trends. This efficient sampling strategy enables us to train a robust surrogate model that breaks through the conventional computational ceiling, accurately projecting the stability-performance Pareto frontier across multiple candidates. The predictive power of the model has been robustly validated against a diverse set of independent literature compositions and further substantiated by our experimental samples.

Beyond computational acceleration, this framework elucidates the physical origins of the giant magnetocaloric effect. Our SHAP-informed analysis establishes a coherent, descriptor-level design logic, revealing that high performance arises from the nonlinear coupling among steric packing, electronic structure and local magnetic moment dispersion. A fundamental design dichotomy emerges: mean descriptors (e.g. mean total unfilled states) predominantly set the baseline of static, phase-specific stability, whereas dispersion descriptors act as the triggers for transformation-related responses.

Crucially, we distilled these insights into a radial guide map for rational design, identifying that the transformation driving force ($\Delta {E}_{{{{\rm{str}}}}}$) and the hysteresis-related volume change ($\Delta {V}_{{{{\rm{ratio}}}}}$) are intrinsically antagonistic. The magnetic moment dispersion acts as the central coupling knob for this trade-off, driving both properties with opposing effects. This insight motivates a hierarchical two-step tuning strategy that can be translated into two rules: first, adjusting the magnetic moment dispersion to place the driving force within a sufficient-but-not-excessive window; and second, refining the reversibility using the hysteresis-selective electronic configuration dispersion handle, while monitoring the baseline electronic constraint. This approach transforms composition refinement from empirical trial-and-error into an interpretable, actionable pathway for navigating the feasible design space.

Finally, while this framework has been established to map the magnetocaloric limits of MM’X compositions, its logic is universal. By demonstrating that structural constraints and local electronic/magnetic dispersion can be treated as distinct, interpretable levers, we provide a streamlined blueprint to discover high-performance materials within the vast, unexplored regions of the HEA landscape. This logic can be seamlessly expanded to other functional or structural properties, leveraging the reliability of DFT to map complex manifolds that are otherwise inaccessible to experiment alone.

## Methods

### Statistics and reproducibility

No statistical method was used to predetermine sample size. No data were excluded from the analyses, except for explicitly stated non-comparable magnetic configurations discussed in the main text and Supplementary Information. The study is computational in nature; therefore, experimental randomisation and blinding were not applicable. All random sampling steps used in configuration creation and machine-learning procedures were performed with fixed random seeds to ensure reproducibility. Statistical analyses, including box-plot summaries and Kruskal–Wallis tests, were performed as described in the relevant ‘Methods’ sections, and the underlying data are provided in the Source Data.

### DFT computation

Spin-polarised density-functional theory (DFT) calculations were performed with the Vienna Ab initio Simulation Package (VASP, v6.4.2)^57,58^ using the Projected-Augmented Wave (PAW) method^59,60^. The exchange and correlation effects were treated under the generalised gradient approximation (GGA) according to the Perdew–Burke–Ernzerhof (PBE) functional^61^ with a plane-wave cutoff energy of 400 eV. For the three compositional series, MnNiSi_1−x_Ge_x_, Mn_*x*_ Fe_1−*x*_ NiSi and MnNi_x_Fe_1−x_Si, we sampled several compositions (*x* = 0, 0.125, 0.25, 0.375, 0.5, 0.625, 0.75, 0.875, 1) in both hexagonal and orthorhombic phases. A family of supercells (1 × 1 × 1 (12 atoms), 2 × 1 × 1, 1 × 2 × 1, 1 × 1 × 2 (24 atoms), 2 × 2 × 1, 1 × 2 × 2, 2 × 1 × 2 (48 atoms)) generated by *Phonopy* (v2.22.1)^62^ were used to realise partial substitutions; cells redundant in the hexagonal setting (since *a* = *b*) were omitted. For each combination of structure, supercell and *x*, site-ordered configurations on the substituted sublattices were generated and reduced to symmetry-inequivalent variants. When several variants remained (which occurs mainly for *x* = 0.375, 0.5 and 0.625), we labelled the lowest-energy variant as the representative configuration and treated the others as non-representative configurations. Initial magnetic moments corresponding to a ferromagnetic state were applied to all structures and compositions. Ionic and cell relaxations were carried out with collinear spin polarisation, using a Gaussian/Methfessel–Paxton smearing (*σ* = 0.10 eV) and Γ-centred k-point meshes targeting a reciprocal-space spacing of ~0.03 Å⁻¹ by VASPKIT (v1.5.1)^63^. Subsequent static single-point calculations on the relaxed geometries were carried out to determine local magnetic moments. Further details of the configuration enumeration and sampling strategy are provided in the Supplementary Methods 1.1–1.2. All optimised atomic coordinates are deposited in idUS (https://doi.org/10.12795/11441/188014).

### Machine-learning surrogate model: training, selection and interpretation

Using a custom-made HTP-DFT automation code, we built a MM’X reference dataset spanning ternary and quaternary stoichiometries, described by MnM’(X_1_X_2_), Mn(M’_1_ M’_2_)X and (MnM)M’X, which represented mixing on the X site, the M’ site and Mn–M-related sublattices, respectively. The elemental selection was M = Mn, Fe, Co, Ni, Cu; M’ = Fe, Co, Ni, Cu; X = Si, Ge, Al, Ti, Sn. To maintain a uniform high-throughput computational protocol, all DFT calculations used for constructing the machine-learning dataset were initialised with ferromagnetic magnetic moments. The final self-consistent solutions were not strictly constrained to retain purely ferromagnetic properties, and a limited number of structures converged to ferrimagnetic or weakly magnetic states. Given that the surrogate models were trained to predict continuous DFT-derived properties, including magnetic moment, energy and volume, rather than magnetic-order classes, these cases were retained in the machine-learning dataset. The versions of all software packages used in this work, beyond those explicitly stated in the main text, are documented in the GitHub repository (Code availability).

We trained supervised multi-output regressors to predict six DFT-level magnitudes per composition: unit-cell volume, *V*, magnetic moment, *m* and energy, *E*, for both orthorhombic and hexagonal structures. The dataset was randomly split into 80% training and 20% test, and all model selection and hyperparameter tuning were carried out only on the training data; the held-out test set was used once for the final performance assessment (see Supplementary Methods 2.1).

For each composition, we computed a set of 132 Magpie^49^ composition descriptors using the ElementProperty featurizer from matminer (v0.9.3), built from 22 elemental properties and six summary statistics per property. On the training set, we applied a layered, label-aware feature selection pipeline^50^ that combined simple variance and mutual-information filters with correlation-based redundancy pruning and a final SHAP (SHapley Additive exPlanations, v0.47.2)^40^-based selection step, yielding a compact and informative descriptor subset for learning. The full descriptor list and filtering procedure are provided in Supplementary Methods 2.2.

We benchmarked a panel of regression algorithms suitable for tabular data, including linear, kernel-based and tree-ensemble models, all implemented in a multi-output framework. Multi-objective hyperparameter optimisation with Optuna (v4.3.0)^64^ (using the NSGA-II sampler^65^) was used to minimise both the mean and the worst-case range-normalised MAE (nMAE) across the six targets, and the best-performing XGBoost ensemble (v3.0.2)^53^ was adopted as the final surrogate model. We reported standard regression metrics (*R*^2^, MAE, RMSE and nMAE); explicit metric definitions, the list of tested models and optimisation settings are provided in Supplementary Methods 2.3–2.5.

Post-hoc interpretability focused on this final XGBoost surrogate trained with the reduced feature set. From the six direct DFT-level outputs we constructed a small set of derived proxies relevant for magnetocaloric design, the hexagonal/orthorhombic formation energy ${E}_{f}^{{{{\rm{hex}}}}}{/E}_{f}^{{{{\rm{ort}}}}}$, the hexagonal/orthorhombic total magnetic moment ${m}_{{{{\rm{hex}}}}}{/m}_{{{{\rm{ort}}}}}$, the structural energy difference $\Delta E={E}_{{{{\rm{hex}}}}}-{E}_{{{{\rm{ort}}}}}$ and the relative volume change $\Delta {V}_{{{{\rm{ratio}}}}}=({V}_{{{{\rm{ort}}}}}-{V}_{{{{\rm{hex}}}}})/{V}_{{{{\rm{ort}}}}}$. The feature importance was then quantified using SHAP values averaged over these targets. The SHAP protocol and the resulting feature-ranking analysis, together with additional dependence plots, are described in Supplementary Methods 2.6.

### Experimental

Polycrystalline HEAs with nominal compositions (FeMnNi)_66.7_(Ge_*x*_Si_1-*x*_)_33.3_ (*x* = 0.45 and 0.50) were synthesised via arc melting high-purity elemental constituents (≥99.9 wt%) under a high-purity argon atmosphere. The ingots were turned over and melted four times to ensure uniformity. While detailed characterisation information (structural, bulk composition and magnetocaloric effect) can be found in our previous work^46^, the lattice parameters used in this study rely on newly acquired neutron diffraction data and are used solely to validate the computational results. Neutron diffraction measurements were performed on the bulk samples at Oak Ridge National Laboratory using the Wide-Angle Neutron Diffractometer (WAND²) diffractometer with a wavelength of 1.488676 Å and within the temperature range of 75–250 K. The data refinement was performed with the FullProf software to extract the lattice parameters. The full neutron diffraction dataset and detailed analysis are currently being prepared for a separate publication.

## Supplementary information

Supplementary Information

Transparent Peer Review File

## Source data

Source data

## Data Availability

The curated dataset generated and analysed in this study has been deposited in the idUS repository under https://doi.org/10.12795/11441/186086. The optimised atomic coordinates of the DFT computational models generated in this study have also been deposited as an idUS database under https://doi.org/10.12795/11441/188014. The structure database includes the optimised hexagonal and orthorhombic models used for the HTP-DFT/ML database, as well as the optimised representative and non-representative supercell structures used for the supercell-consistency analysis. The Source Data generated in this study are provided with this paper in the Source Data file. Additional supporting data generated in this study are provided in the Supplementary Information. No restricted-access data were used in this study. Source data are provided with this paper.

## Code availability

The custom code used in this study is available in the GitHub repository at https://github.com/zhecui592/DFT-ML-Magnetocaloric-HEAs and has been deposited in the idUS repository under https://doi.org/10.12795/11441/186089.
