# Supplementary material for: DFT-based machine-learning for the rational design of magnetocaloric high-entropy alloys
Source: Nat Commun. 2026 Aug 25;17:10685. doi: 10.1038/s41467-026-77123-w (PMC13654988; doi:10.1038/s41467-026-77123-w)
Supplement: Supplementary file 1 [file 41467_2026_77123_MOESM1_ESM.pdf]

Supplementary Information

DFT-based Machine-Learning for the Rational Design of Magnetocaloric High-Entropy Alloys

Zhe Cui (崔哲)[1], Carlos Romero-Muñiz[1], Jia Yan Law[1], Victorino Franco[1*]

[1]Multidisciplinary Unit for Energy Science, Department of Condensed Matter Physics, University of Seville, 41012-Seville, Spain. [*]email: vfranco@us.es.

Supplementary Figures

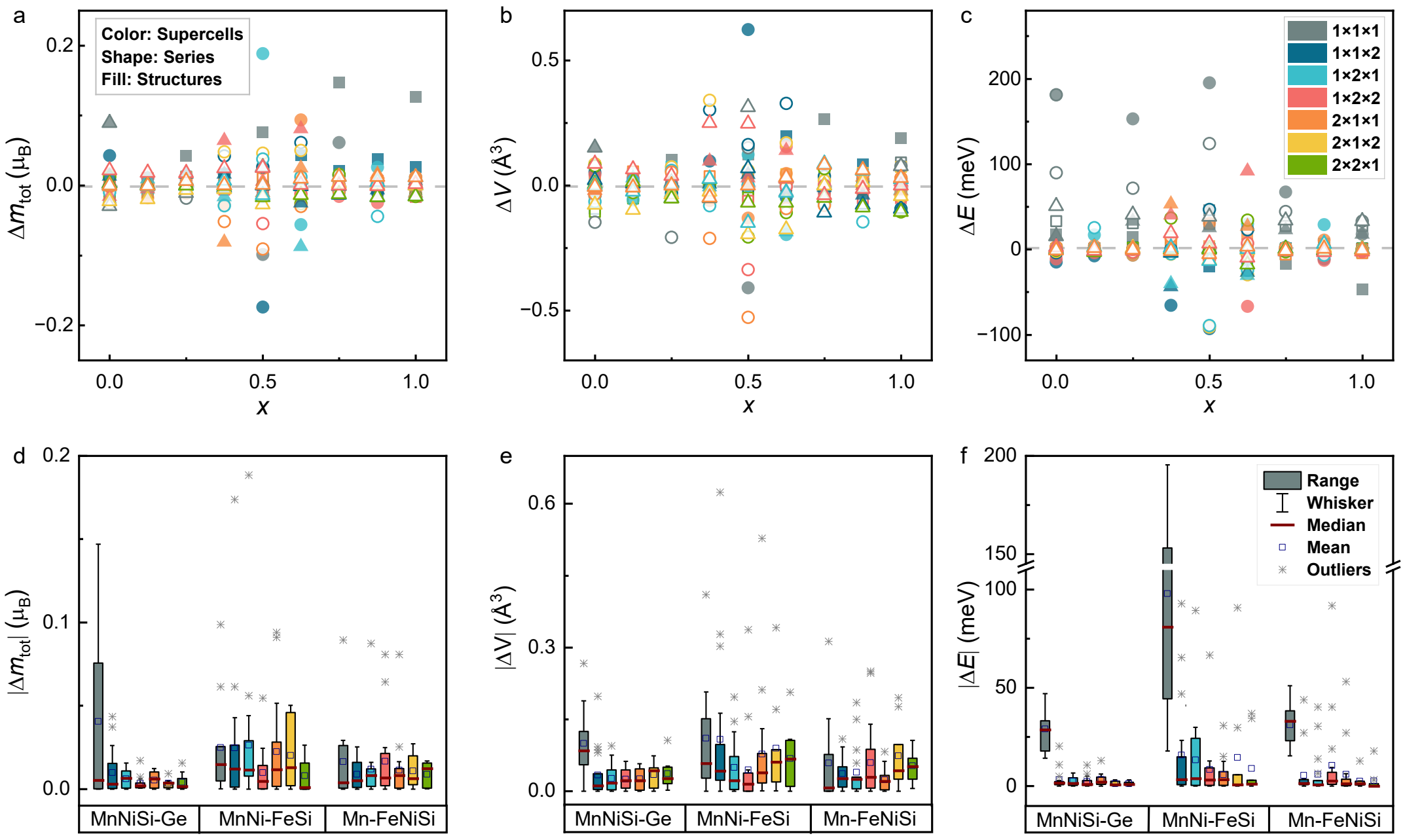

Supplementary Figure 1. Detailed residual and supercell-resolved deviation distributions supporting Fig. 2. Top panels show residuals $|\Delta P| = |P_{rep} - P_{ref}|$ as a function of composition x for magnetic moment, volume and energy. Bottom panels show the corresponding absolute deviation magnitudes, $|\Delta P|$, grouped by substitution series and supercell size. Colours denote supercell size, symbol shapes denote substitution series, and filled/open symbols distinguish crystal structures. Boxes indicate the interquartile range (IQR), centre lines indicate medians, whiskers extend to 1.5×IQR, and points outside the whiskers indicate outliers. For each box-plot group, n denotes the number of independent computational entries used to calculate the distribution, with exact values provided in the Source Data.

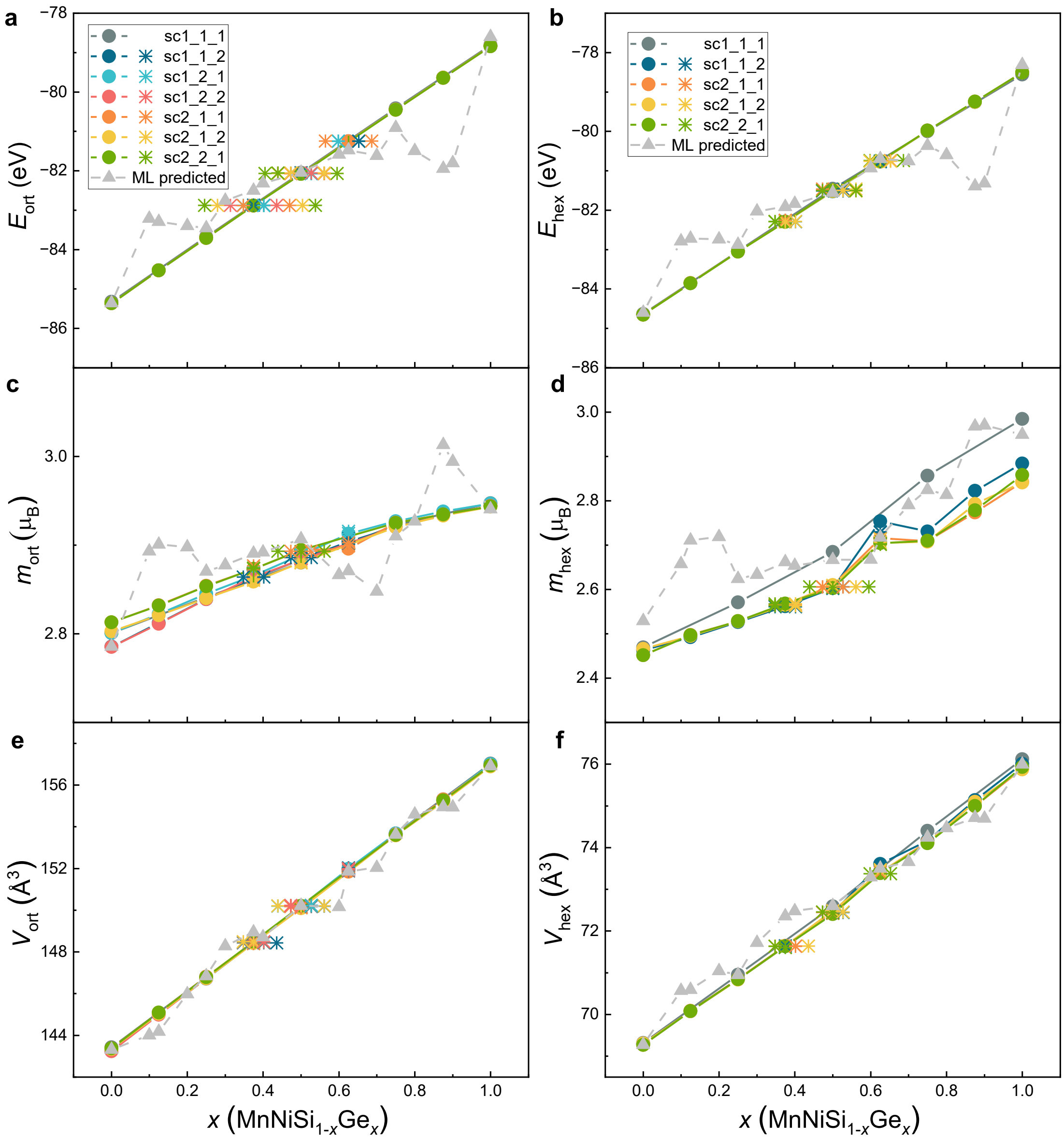

Supplementary Figure 2. DFT results (supercells and enumerated variants) and their comparison with XGB predictions for the MnNiSi–Ge composition series. **a**, Orthorhombic and **b**, hexagonal energies as a function of Ge content; **c–d**, magnetic moments; **e–f**, volumes. Coloured symbols represent individual DFT calculations for different supercells and configurational variants; the supercell label "sc_*" in the legend denotes the corresponding supercell set, solid lines connect the representative configurations (circles), whereas asterisk symbol shapes indicate additional non-representative configurations or alternative symmetry-compatible supercells at the same composition *x*. A tiny x horizontal jitter displacement is applied for visual separation of overlapping points. The dashed grey line denotes the XGB surrogate prediction evaluated at the same range of nominal compositions.

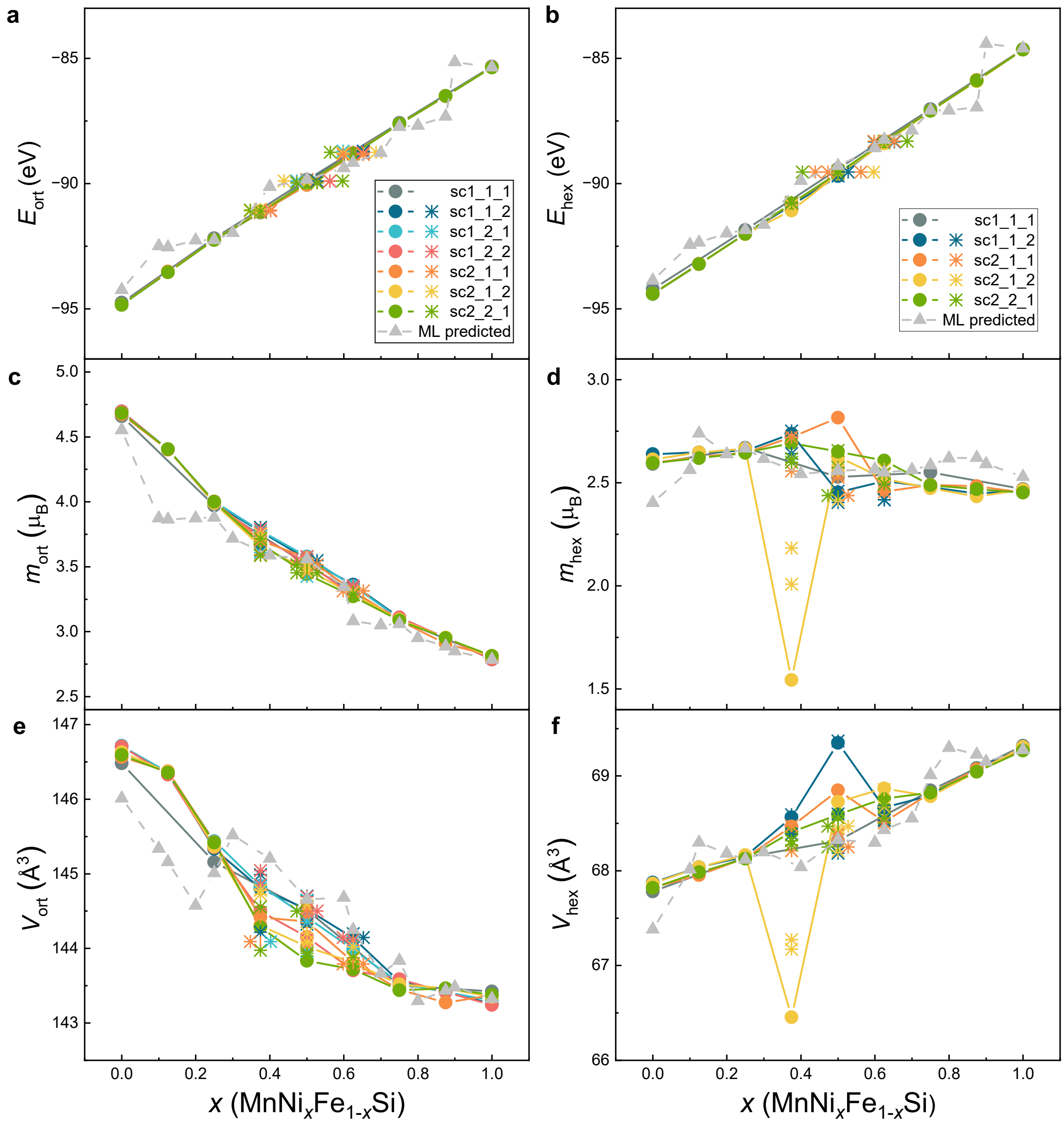

Supplementary Figure 3. DFT results (supercells and enumerated variants) and their comparison with XGB predictions for the Mn–FeNiSi composition series. **a**, Orthorhombic and **b**, hexagonal energies as a function of Ge content; **c–d**, magnetic moments; **e–f**, volumes. Coloured symbols represent individual DFT calculations for different supercells and configurational variants; the supercell label "sc_*" in the legend denotes the corresponding supercell set, solid lines connect the representative configurations (circles), whereas asterisk symbol shapes indicate additional non-representative configurations or alternative symmetry-compatible supercells at the same composition x. A tiny x horizontal jitter displacement is applied for visual separation of overlapping points. The dashed grey line denotes the XGB surrogate prediction evaluated at the same range of nominal compositions.

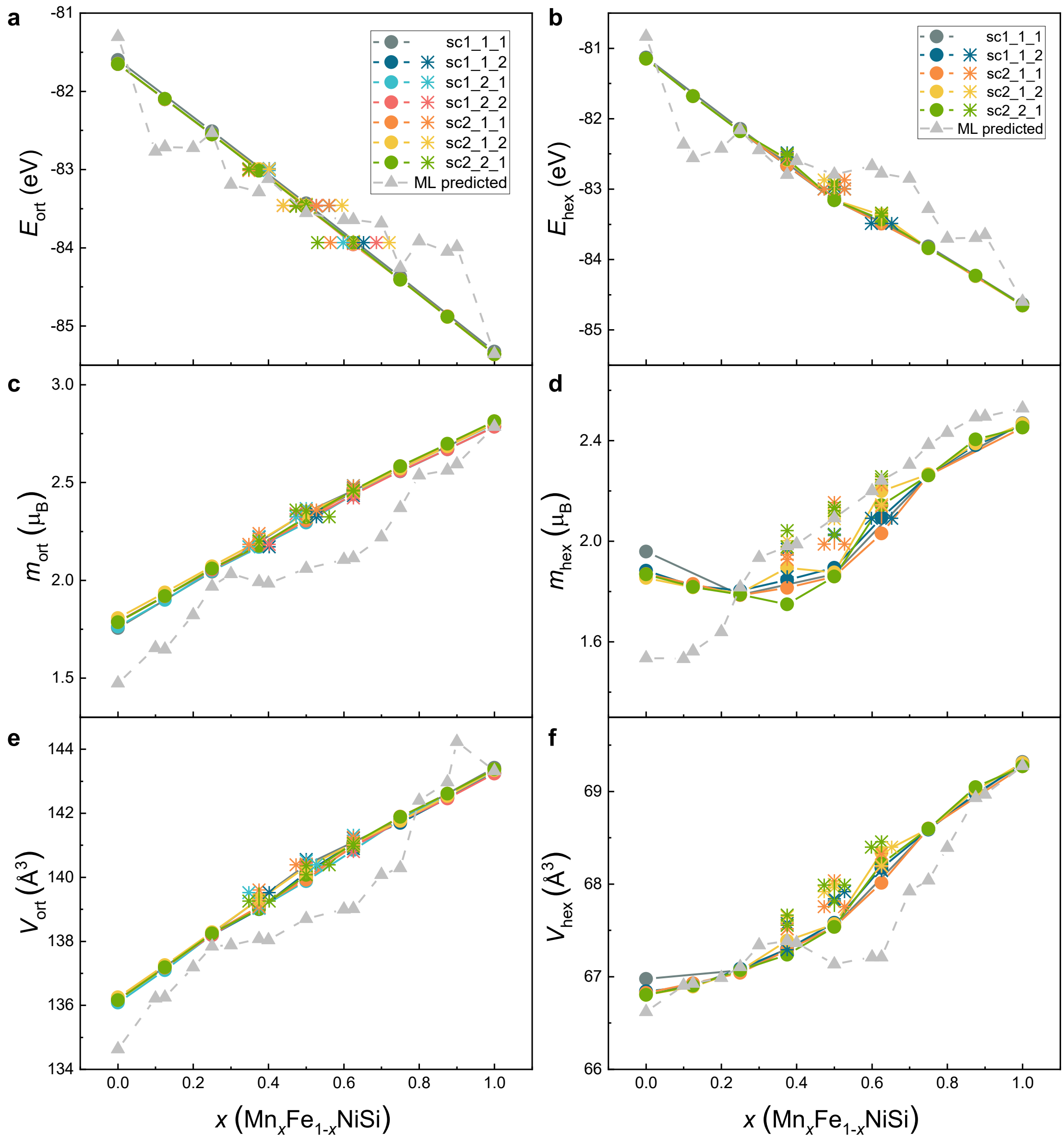

Supplementary Figure 4. DFT results (supercells and enumerated variants) and their comparison with XGB predictions for the MnNi–FeSi composition series. **a**, Orthorhombic and **b**, hexagonal energies as a function of Ge content; **c–d**, magnetic moments; **e–f**, volumes. Coloured symbols represent individual DFT calculations for different supercells and configurational variants; the supercell label "sc_*" in the legend denotes the corresponding supercell set, solid lines connect the representative configurations (circles), whereas asterisk symbol shapes indicate additional non-representative configurations or alternative symmetry-compatible supercells at the same composition *x*. A tiny *x* horizontal jitter displacement is applied for visual separation of overlapping points. The dashed grey line denotes the XGB surrogate prediction evaluated at the same range of nominal compositions.

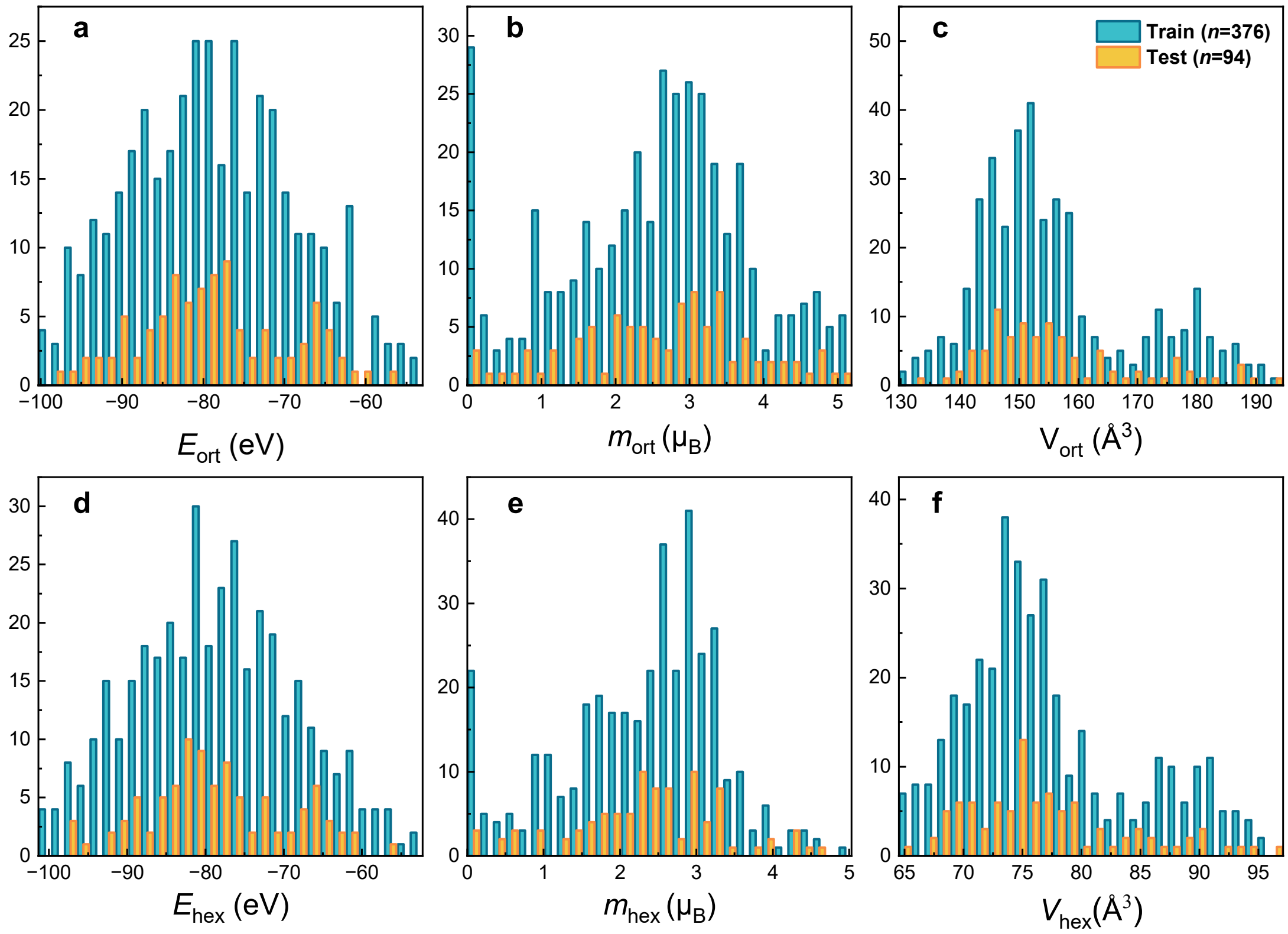

Supplementary Figure 5. Distributions of the six DFT targets and their train–test split. Panels **a–c** show the orthorhombic energy E_{ort} ,volume V_{ort} and magnetic moment m_{ort} ;panels **d–f** show the corresponding hexagonal quantities E_{hex} ,$V_{hex}$and m_{hex} .Blue bars indicate training data (*n* = 376), and orange bars indicate test data (*n* = 94), illustrating that both sets sample similar regions of the target space.

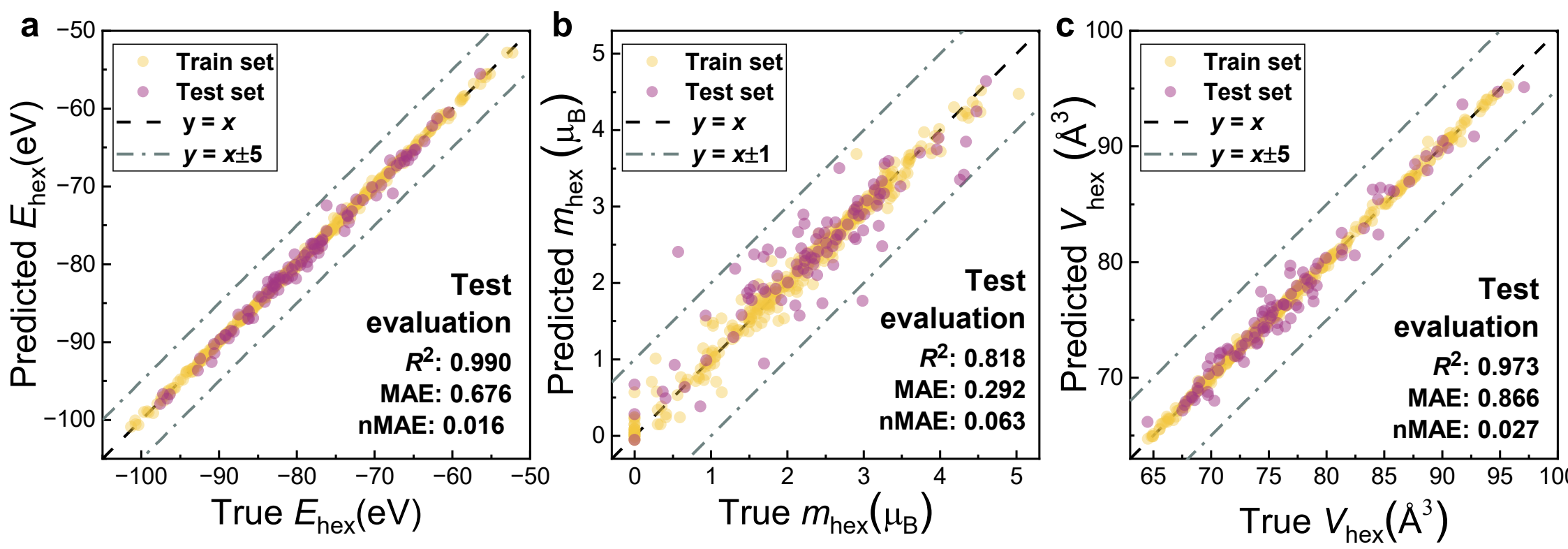

Supplementary Figure 6. Parity plots for the final XGB surrogate. Predicted versus DFT values are shown for **a**, hexagonal total energy (E_{hex}), **b**, hexagonal magnetic moment (m_{hex}), and **c**, hexagonal volume (V_{hex}). symbols show test-set predictions versus the corresponding DFT values, the dashed line indicates perfect agreement (*y* = *x*), and the dash-dotted lines mark an absolute-error band of ±nMAE.

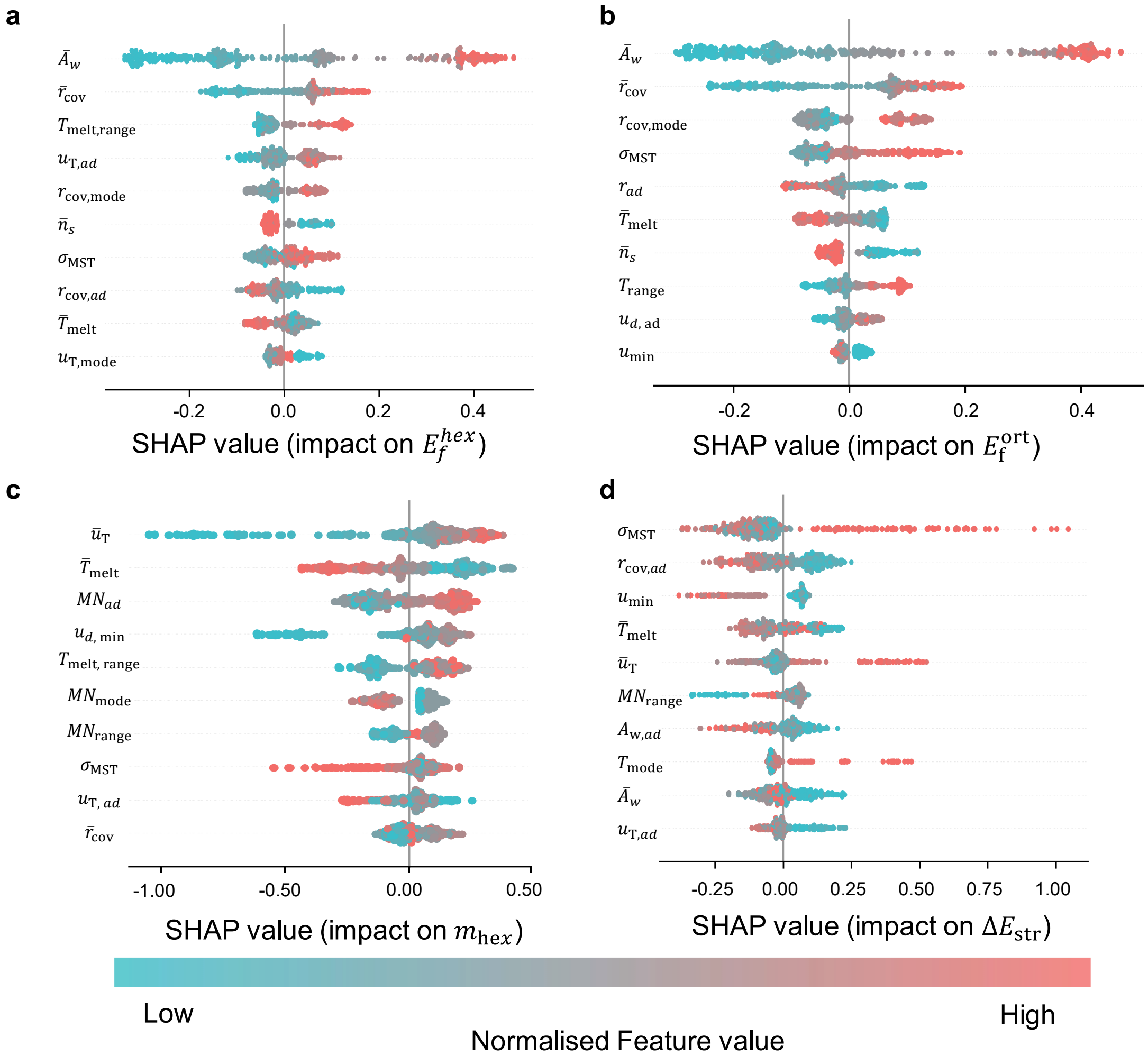

Supplementary Figure 7. SHAP interpretation of the XGB surrogate. SHAP summary plots for (a) $E_{\mathrm{f}}^{\mathrm{hex}}$, (b) $E_{\mathrm{f}}^{\mathrm{ort}}$, (c) m_{hex}. and (d) ΔE_{str}. Features are ordered by decreasing importance (mean | SHAP |); each point corresponds to one sample, and its colour encodes the feature value (blue: low, red: high). Feature codes follow **Table 3**.

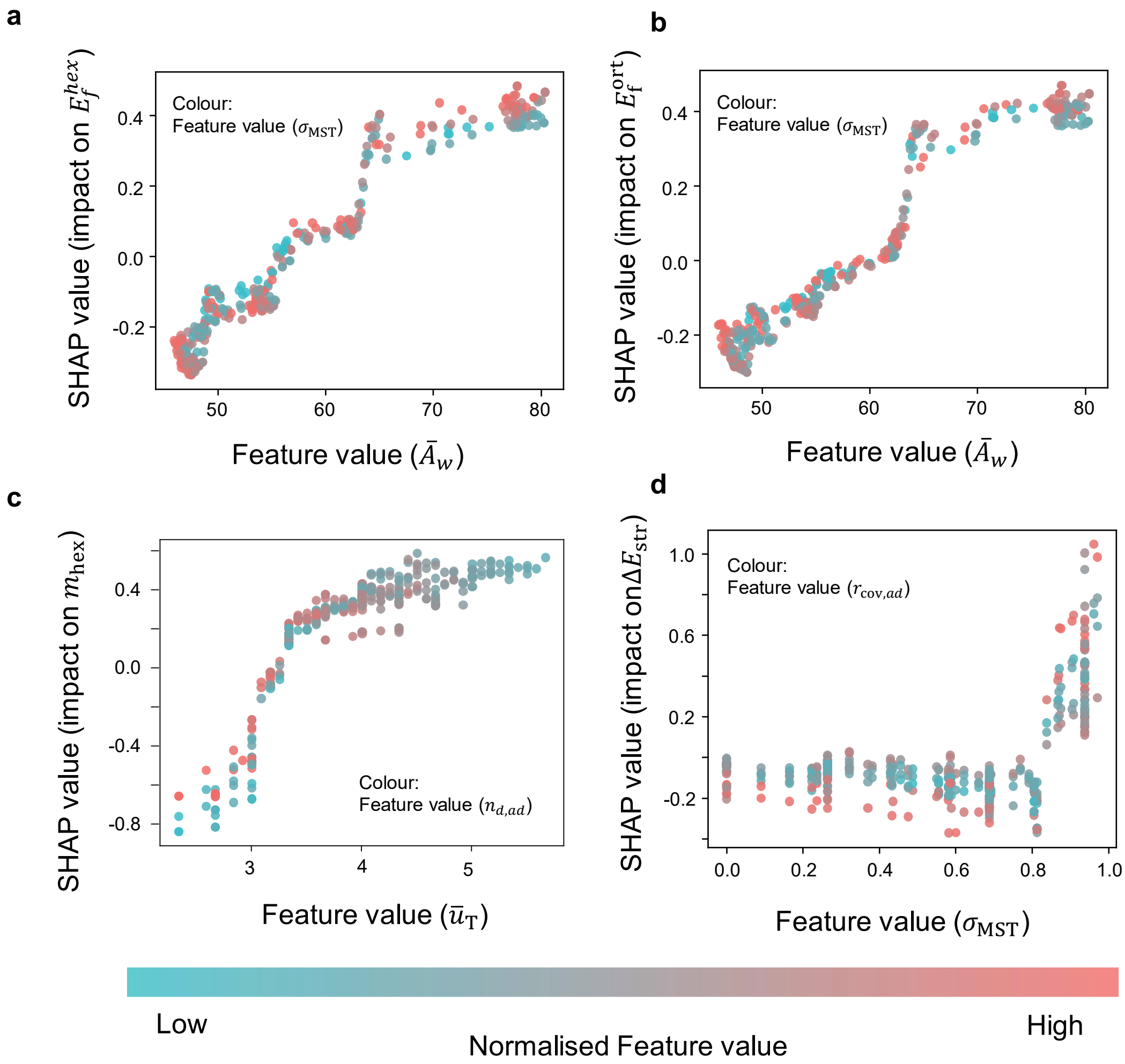

Supplementary Figure 8. SHAP interpretation of the XGB surrogate. SHAP dependence plots for key descriptors: (a) $\bar{A}_w$ versus E_f^{hex}, (b) $\bar{A}_w$ versus E_f^{ort}, (d) $\bar{u}_T$ versus m_{hex} and (d) σ_{MST} versus ΔE_{str}. Feature codes follow **Table 3**.

Supplementary Tables

Supplementary Table 1. List of features generated by the Magpie method and their assigned codes

Code	Property	Code	Property
F_1	Atomic Number	F_12	*f* Valence Electrons
F_2	Mendeleev Number	F_13	Total Valence Electrons
F_3	Atomic Weight	F_14	Unfilled s States
F_4	Melting Temperature	F_15	Unfilled p States
F_5	Column (Group)	F_16	Unfilled d States
F_6	Row (Period)	F_17	Unfilled f States
F_7	Covalent Radius	F_18	Total Unfilled States
F_8	Electronegativity	F_19	Specific Volume (0 K ground state)
F_9	*s* Valence Electrons	F_20	Band-gap Energy (0 K ground state)
F_10	*p* Valence Electrons	F_21	Magnetic Moment (per atom, 0 K)
F_11	*d* Valence Electrons	F_22	Space-Group Number (0 K ground state)

Supplementary Table 2. Dispersion statistics for three properties (energy *E*, volume *V*, and magnetic moment *m*). For each property, the "Pooled" rows (all three series, both hexagonal (hex)/orthorhombic (ort) phase) report the median, 95th percentile (P95), and maximum (Max.) of the absolute deviation $|\Delta P| = |P_{rep} - P_{ref}|$, together with the Kruskal–Wallis test statistic (KW_H) and *p* value (KW_p) across supercells. Here P_{ref} is the per-x median of representative values across supercells within the same series and structure. "Per-series" rows list Median/P95/Max per series (KW is computed on pooled data only). n denotes the number of independent computational entries used to calculate each distribution.

Property	Series	n	Median	P95	Max.	KW_*H*	KW_*p*
	Pooled	294	2.172	48.461	195.451	70.429	3.34×10^{-13}
ΔE	$Mn_xFe_{1-x}NiSi$	99	1.843	28.081	47.105	-	-
[meV/f.u.]	$MnNi_xFe_{1-x}Si$	98	3.513	91.147	195.451	-	-
	$MnNiSi_{1-x}Ge_x$	97	1.699	40.270	91.791	-	-
	Pooled	294	0.027	0.197	0.624	12.669	0.049
ΔV	$Mn_xFe_{1-x}NiSi$	99	0.024	0.103	0.267	-	-
[Å^3/f.u.]	$MnNi_xFe_{1-x}Si$	98	0.034	0.330	0.624	-	-
	$MnNiSi_{1-x}Ge_x$	97	0.027	0.178	0.313	-	-
	Pooled	294	0.007	0.058	0.188	4.096	0.664
Δm	$Mn_xFe_{1-x}NiSi$	99	0.003	0.038	0.147	-	-
[μB/f.u.]	$MnNi_xFe_{1-x}Si$	98	0.011	0.066	0.188	-	-
	$MnNiSi_{1-x}Ge_x$	97	0.007	0.033	0.089	-	-

Supplementary Table 3. Transposed comparison of computational costs between minimal (24-atom) and enlarged (48-atom) supercells for the $Mn_xFe_{1-x}SiGe$ series. hex and ort denote calculations in the hexagonal and orthorhombic phases; opt and scf denote geometry optimizations and single-point self-consistent calculations. For each phase–stage condition, we report mean total wall time (⟨wall⟩; extracted from OUTCAR as Elapsed time (sec)), wall time per ionic step ⟨wall/ion⟩, and wall time per electronic SCF cycle ⟨wall/scf⟩, together with speedup ratios (Speedup = t_{48}/t_{24}) and the fractional time savings obtained by using the 24-atom supercell instead of the 48-atom supercell (Time saved = $1 - t_{24}/t_{48}$). N(24)/N(48) indicates the numbers of timing records used for 24-atom and 48-atom supercells in each condition.

Metric	hex–opt	hex–scf	ort–opt	ort–scf
Phase	hex	hex	ort	ort
Stage	opt	scf	opt	scf
N(24)	32	28	48	42
N(48)	32	28	48	42
⟨wall⟩_24 (s)	4985.6	644.9	3098.2	450.0
⟨wall⟩_48 (s)	13281.5	1432.1	7660.2	1022.9
⟨wall/ion⟩_24 (s)	235.1	644.9	178.6	450.0
⟨wall/ion⟩_48 (s)	558.4	1432.1	366.1	1022.9
⟨wall/scf⟩_24 (s)	16.7	17.9	13.6	14.5
⟨wall/scf⟩_48 (s)	30.7	33.3	26.4	28.1
Speedup (wall)	2.66	2.22	2.47	2.27
Speedup (wall/ion)	2.38	2.22	2.05	2.27
Speedup (wall/scf)	1.85	1.86	1.95	1.94
Time saved (%)	62.5	55.0	59.6	56.0

Supplementary Table 4. Transposed comparison of computational costs between minimal (24-atom) and enlarged (48-atom) supercells for the $MnNi_xFe_{1-x}Si$ series. hex and ort denote calculations in the hexagonal and orthorhombic phases; opt and scf denote geometry optimizations and single-point self-consistent calculations. For each phase–stage condition, we report mean total wall time (⟨wall⟩; extracted from OUTCAR as Elapsed time (sec)), wall time per ionic step ⟨wall/ion⟩, and wall time per electronic SCF cycle ⟨wall/scf⟩, together with speedup ratios (Speedup = t_{48}/t_{24}) and the fractional time savings obtained by using the 24-atom supercell instead of the 48-atom supercell (Time saved = $1 - t_{24}/t_{48}$). N(24)/N(48) indicates the numbers of timing records used for 24-atom and 48-atom supercells in each condition.

Metric	hex–opt	hex–scf	ort–opt	ort–scf
Phase	hex	hex	ort	ort
Stage	opt	scf	opt	scf
N(24)	28	28	42	42
N(48)	28	28	42	42
⟨wall⟩_24 (s)	5234.6	595.7	4614.3	457.4
⟨wall⟩_48 (s)	12639.3	1302.3	9242.7	1032.4
⟨wall/ion⟩_24 (s)	205.2	595.7	160.4	457.4
⟨wall/ion⟩_48 (s)	431.8	1302.3	368.9	1032.4
⟨wall/scf⟩_24 (s)	15.7	16.9	13.5	14.6
⟨wall/scf⟩_48 (s)	29.3	31.7	26.5	28.4
Speedup (wall)	2.41	2.19	2.00	2.26
Speedup (wall/ion)	2.10	2.19	2.30	2.26
Speedup (wall/scf)	1.86	1.87	1.97	1.95
Time saved (%)	58.6	54.3	50.1	55.7

Supplementary Table 5. Transposed comparison of computational costs between minimal (24-atom) and enlarged (48-atom) supercells for the $MnNiSi_{1-x}Ge_x$ series. hex and ort denote calculations in the hexagonal and orthorhombic phases; opt and scf denote geometry optimizations and single-point self-consistent calculations. For each phase–stage condition, we report mean total wall time (⟨wall⟩; extracted from OUTCAR as Elapsed time (sec)), wall time per ionic step ⟨wall/ion⟩, and wall time per electronic SCF cycle ⟨wall/scf⟩, together with speedup ratios (Speedup = t_{48}/t_{24}) and the fractional time savings obtained by using the 24-atom supercell instead of the 48-atom supercell (Time saved = $1 - t_{24}/t_{48}$). N(24)/N(48) indicates the numbers of timing records used for 24-atom and 48-atom supercells in each condition.

Metric	hex–opt	hex–scf	ort–opt	ort–scf
Phase	hex	hex	ort	ort
Stage	opt	scf	opt	scf
N(24)	30	27	45	42
N(48)	30	28	44	33
⟨wall⟩_24 (s)	3088.4	472.1	3206.3	427.2
⟨wall⟩_48 (s)	8222.3	1157.5	9112.1	928.2
⟨wall/ion⟩_24 (s)	172.6	472.1	151.7	427.2
⟨wall/ion⟩_48 (s)	428.7	1157.5	399.9	928.2
⟨wall/scf⟩_24 (s)	15.5	16.4	14.7	15.5
⟨wall/scf⟩_48 (s)	33.2	35.6	34.4	30.5
Speedup (wall)	2.66	2.45	2.84	2.17
Speedup (wall/ion)	2.48	2.45	2.64	2.17
Speedup (wall/scf)	2.15	2.16	2.34	1.97
Time saved (%)	62.4	59.2	64.8	54.0

Supplementary Table 6. Ten-fold cross-validation performance of all candidate surrogate models. For each model we report the coefficient of determination (R^2) and the normalised mean absolute error (nMAE) for the six DFT-level targets (hexagonal (hex)/orthorhombic (ort) energies E, magnetic moments m, and volumes V). The last column ("Avg.") gives the average value over all targets.

model	metric	E		m		V		Avg.
		hex	ort	hex	ort	hex	ort	
RF	R^2	0.983	0.984	0.833	0.907	0.964	0.965	0.939
	nMAE	0.024	0.025	0.063	0.054	0.035	0.034	0.039
XGBoost	R^2	0.991	0.992	0.852	0.913	0.975	0.974	0.950
	nMAE	0.017	0.016	0.06	0.05	0.029	0.029	0.034
GBR	R^2	0.975	0.976	0.799	0.888	0.958	0.954	0.925
	nMAE	0.03	0.03	0.076	0.063	0.041	0.041	0.047
KNN	R^2	0.96	0.961	0.761	0.852	0.941	0.928	0.901
	nMAE	0.037	0.037	0.079	0.07	0.045	0.048	0.053
SVR (rbf)	R^2	0.993	0.995	0.771	0.875	0.982	0.974	0.932
	nMAE	0.015	0.013	0.086	0.071	0.025	0.029	0.04
SVR (lin)	R^2	0.992	0.993	0.706	0.853	0.972	0.954	0.912
	nMAE	0.016	0.016	0.095	0.077	0.032	0.041	0.046
GPR	R^2	0.994	0.998	0.758	0.859	0.947	0.952	0.918
	nMAE	0.012	0.008	0.081	0.062	0.036	0.035	0.039
LinReg	R^2	0.995	0.996	0.748	0.86	0.972	0.954	0.921
	nMAE	0.013	0.011	0.087	0.074	0.032	0.041	0.043
Ridge	R^2	0.993	0.994	0.728	0.86	0.973	0.954	0.917
	nMAE	0.016	0.015	0.091	0.073	0.032	0.041	0.045
Lasso	R^2	0.995	0.996	0.746	0.861	0.973	0.954	0.921
	nMAE	0.013	0.011	0.087	0.073	0.032	0.041	0.043
ElasticNet	R^2	0.995	0.996	0.744	0.861	0.973	0.954	0.92
	nMAE	0.013	0.012	0.088	0.073	0.032	0.041	0.043
BayesRidge	R^2	0.995	0.996	0.724	0.859	0.973	0.954	0.917
	nMAE	0.013	0.011	0.092	0.074	0.032	0.041	0.044
Huber	R^2	0.995	0.996	0.747	0.859	0.972	0.954	0.92
	nMAE	0.013	0.011	0.087	0.071	0.033	0.04	0.043
PLS	R^2	0.986	0.987	0.682	0.852	0.973	0.952	0.905
	nMAE	0.021	0.022	0.1	0.075	0.032	0.041	0.049
MLP	R^2	0.978	0.98	0.803	0.899	0.951	0.94	0.925
	nMAE	0.024	0.023	0.072	0.057	0.038	0.041	0.042

Supplementary Table 7. Performance of the optimised RF, XGB, and RF+XGB stacking models on the held-out test set. The coefficient of determination (R^2) and normalised mean absolute error (nMAE) are reported for the six DFT-level targets (hexagonal (hex)/orthorhombic (ort) energies *E*, magnetic moments *m*, and volumes *V*). “Avg.” denotes the average over all targets.

Model	Metric		*E*		*m*		*V*		Avg.
			hex	ort	hex	ort	hex	ort	
XGBoost	train	R^2	0.999	1.000	0.982	0.992	0.999	0.999	0.995
		nMAE	0.003	0.003	0.017	0.014	0.005	0.005	0.008
	test	R^2	0.990	0.988	0.818	0.883	0.973	0.962	0.936
		nMAE	0.016	0.016	0.064	0.052	0.027	0.029	0.034
RF	train	R^2	0.992	0.992	0.931	0.961	0.986	0.986	0.975
		nMAE	0.014	0.015	0.035	0.033	0.020	0.019	0.023
	test	R^2	0.981	0.975	0.825	0.866	0.955	0.944	0.924
		nMAE	0.023	0.025	0.066	0.060	0.034	0.036	0.041
Stacking (RF+XGB)	train	R^2	0.999	1.000	0.984	0.993	0.999	0.999	0.996
		nMAE	0.003	0.003	0.016	0.014	0.005	0.005	0.008
	test	R^2	0.990	0.989	0.808	0.881	0.972	0.962	0.934
		nMAE	0.016	0.016	0.065	0.053	0.027	0.029	0.034

Supplementary Methods

1 DFT calculations and configuration generation

1.1 Configuration enumeration and selection

For each of the three series $MnNiSi_{1-x}Ge_x$, $Mn_xFe_{1-x}NiSi$, and $MnNi_xFe_{1-x}Si$, we used the same composition grid as described in the main text (from x = 0 to x = 1in steps of 0.125) in both hexagonal and orthorhombic structures. Substitutions were realised using the following family of supercells by Phonopy [1]: 1×1×1 (12 atoms), 2×1×1, 1×2×1, 1×1×2 (24 atoms), 2×2×1, 1×2×2, 2×1×2 (48 atoms). In the hexagonal setting with a = b, supercells that are symmetry-equivalent to 1×2×1 and 1×2×2 are redundant and were therefore omitted.

For each combination of crystal structure, supercell, and composition x, we first generated site-ordered configurations on the substituted sublattice(s).

- For x = 0.5, all occupations compatible with the supercell size and stoichiometry were explicitly enumerated.
- For x = 0.375 and x = 0.625, where the number of distinct occupations grows combinatorially, configurations were generated by random sampling under the same stoichiometric constraint (3 for each).
- For four-fold-volume supercells (e.g. 2×2×1, 1×2×2, 2×1×2), we applied fixed-seed random sampling to pre-select a manageable pool of candidates before applying the selection rules described below.

In all cases, symmetry-equivalent duplicates were removed using a standard structure-matching filter implemented in Python with the pymatgen library [2–4]. Random sampling was performed with fixed-seed NumPy random generators to ensure full reproducibility.

From the remaining symmetry-inequivalent configurations for each (structure, supercell, x) set, we selected a small number of ordered variants based on simple geometric descriptors:

1. Clustered – configurations in which like species tend to occupy neighbouring sites and the substituent atoms form compact clusters. These were identified by minimising the average like–like nearest-neighbour distance on the substituted sublattice(s).
2. Layered – configurations in which the substituent atoms are concentrated into planes perpendicular to a lattice axis, giving a compositionally layered arrangement rather than a uniform distribution. These were identified by maximising a layering/segregation metric along a chosen lattice direction.
3. Dispersed – configurations in which substituent atoms are distributed as uniformly as possible, avoiding both clustering and layering. These were identified by maximising like–like separation while keeping the layering metric low (near-random distribution).

In most of the compositions the underlying crystallographic symmetry yields a single symmetry-inequivalent configuration per structure, supercell. In these cases, we simply took this configuration as the representative variant. For compositions with multiple candidates remaining after symmetry filtering (which occurs for x = 0.375, 0.5 and 0.625), we performed spin-polarised DFT relaxations for all selected clustered, layered and dispersed variants. For a given (structure, supercell, x) set, the variant with the lowest per-

atom total energy was labelled as the representative configuration, whereas the remaining variants were treated as non-representative configurations and used only to assess the sensitivity to supercell choice.

1.2 DFT calculation details

All DFT calculations were carried out with VASP [5,6] using the PAW–PBE [7,8] (GGA–PBE [9]) exchange–correlation functional and a plane-wave cutoff energy of 400 eV. Calculations were spin-polarised and collinear (ISPIN = 2) and started from a ferromagnetic initial state specified via the MAGMOM tag, with initial moments of $4\mu_B$ on Mn, $3.5\mu_B$ on Ni/Fe, and $0\,\mu_B$ on Si/Ge. The same initial magnetic configuration was used for all structures and compositions.

Geometry optimisation followed a two-stage protocol. First, ionic positions and cell parameters were relaxed with ISIF = 3 until the total-energy change and maximum residual forces were below EDIFF = 1×10^{-6} eV and EDIFFG = −0.005 eV $Å^{-1}$, respectively (maximum residual force ≤ 0.005 eV $Å^{-1}$). Gaussian smearing was employed with ISMEAR = 0 and SIGMA = 0.10 eV during these relaxations. In the second stage, static single-point calculations were performed at the relaxed geometries to obtain accurate total energies, volumes and magnetic moments.

Γ-centred Monkhorst–Pack k-point meshes were generated with VASPKIT[10], targeting an approximately constant reciprocal-space spacing of =0.03 $Å^{-1}$. The k-point density was thus automatically scaled with supercell size so that all calculations used a comparable k-point sampling. All configuration generation, symmetry filtering and metric evaluation were implemented in Python using pymatgen[2–4] and custom scripts.

2 Machine-learning surrogate model: additional methodological details

This section provides additional methodological details for the machine-learning surrogate model described in the main text, including the definition of the targets and data split (2.1), feature construction and filtering (2.2), the list of candidate algorithms and training settings (2.3), the evaluation metrics (2.4), the multi-objective hyperparameter optimisation and stacking strategy (2.5), and the SHAP-based interpretability protocol (2.6).

2.1 Targets and dataset split

For each composition we consider six DFT-level target quantities:

- orthorhombic unit-cell volume V_{ort},
- hexagonal unit-cell volume V_{hex},
- orthorhombic magnetic moment m_{ort},
- hexagonal magnetic moment m_{hex},
- orthorhombic total energy E_{ort},
- hexagonal total energy E_{hex}.

The full dataset is randomly split into 80% training and 20% test compositions. All model selection, feature filtering and hyperparameter tuning are performed exclusively on the training split. The test split is never used during model development and is evaluated once at the end to obtain the reported test-set metrics.

2.2 Feature construction and filtering pipeline

For each composition, we construct a Magpie[11] descriptor vector using 22 elemental properties, including:

- atomic number, Mendeleev number, atomic weight, melting point,
- period and group in the periodic table,
- covalent radius and electronegativity,
- s/p/d/f valence electron counts and corresponding numbers of unfilled valence states,
- total valence electron count and total unfilled valence states,
- ground-state atomic volume, band gap, magnetic moment, and space-group number.

For each property, we compute six statistics over the composition: minimum, maximum, range, mean, average absolute deviation and mode. This yields $22 \times 6 = 132$ composition-only features. Feature names follow F_i_j where $i \in [1,22]$ indexes the elemental property and $j \in [1,6]$ the statistic. The full mapping between (i, j) and physical properties/statistics is provided in Supplementary Table 1.

On the training split we apply a layered, label-aware feature filtering pipeline implemented in *scikit-learn*[12] and *TreeSHAP*[13]:

a) Low-variance filtering.

Features whose variance on the training set falls below a small threshold are removed as effectively constant and uninformative.

b) Mutual-information (MI) screening[14,15].

For each target we compute the mutual information between each feature and the target. Features whose MI exceeds a small per-target threshold are retained, and we take the union of retained features across all six targets to preserve features that are informative for at least one label.

c) Correlation-based redundancy pruning.

On the union set, we compute pairwise Pearson correlations and apply a greedy max-relevance min-redundancy (mRMR-like[17]) pruning framework that (i) maximises the absolute feature–label correlation and (ii) minimises residual feature–feature redundancy. Highly collinear features above a prescribed correlation threshold are removed, retaining only one representative from each correlated group.

d) SHAP-based pruning[16].

A strong baseline tree model is fitted on the remaining feature set. SHAP values are computed across the six targets, and the mean absolute SHAP value is used as a feature-importance score. We retain features that either belong to the global top-k by mean | SHAP| or exceed a small SHAP-importance floor τ. The values of $k$and τ are chosen based on validation performance.

This pipeline yields a compact, label-aware subset of composition-only descriptors that is used in all subsequent model comparisons and hyperparameter studies.

2.3 Candidate algorithms and baseline training

We benchmark a diverse set of regression algorithms suitable for small- to medium-sized tabular datasets, implemented using *scikit-learn*[12] and XGBoost[18] library:

- Tree-based ensembles: Random Forest (RF), Gradient Boosting Regressor (GBR) and XGBoost (XGB).
- Distance- and kernel-based methods: k-nearest neighbours (k-NN), support-vector regression (SVR; linear and radial basis function (RBF) kernels), and Gaussian process regression (GPR).
- Linear and robust models: ordinary least squares (OLS; LinearRegression), Ridge, Lasso, ElasticNet, Bayesian ridge regression (BRR), and Huber regression.
- Other baselines: partial least squares (PLS) regression and a shallow multilayer perceptron (MLP).

All models are wrapped in scikit-learn's MultiOutputRegressor to jointly predict the six targets. For non-tree models we employ a Pipeline of the form StandardScaler to regressor to ensure proper feature scaling.

During baseline screening, we do not tune any hyperparameters: each algorithm is trained with its library defaults on the training split and compared on an internal validation split derived from the training data. This baseline comparison is used to identify the most promising model families for multi-objective hyperparameter optimisation (Supplementary Methods 2.5).

2.4 Evaluation metrics

For each target we report coefficient of determination (R^2), mean absolute error (MAE), root-mean-square error (RMSE), and range-normalised MAE (nMAE).

For a given target with true values $\mathrm{y_i}$, predictions $\hat{\mathrm{y}}_\mathrm{i} (\mathrm{i} = 1, \ldots, \mathrm{N})$, and $\bar{\mathrm{y}} = \frac{1}{\mathrm{N}} \sum_\mathrm{i} \mathrm{y_i}$, these metrics are defined as:

$$R^2 = 1 - \frac{\sum_{\mathrm{i}=1}^{\mathrm{N}} (\mathrm{y_i} - \hat{\mathrm{y}}_\mathrm{i})^2}{\sum_{\mathrm{i}=1}^{\mathrm{N}} (\mathrm{y_i} - \bar{\mathrm{y}})^2} \quad (1)$$

$$\mathrm{MAE} = \frac{1}{N} \sum_{i=1}^{N} \mid y_i - \hat{y}_i \mid \quad (2)$$

$$\mathrm{RMSE} = \sqrt{\frac{1}{N} \sum_{i=1}^{N} (y_i - \hat{y}_i)^2} \quad (3)$$

$$\mathrm{nMAE} = \frac{\mathrm{MAE}}{\max_\mathrm{i}(\mathrm{y_i}) - \min_\mathrm{i}(\mathrm{y_i})} \quad (4)$$

For each model, we report these metrics per target. Aggregate performance across the six targets is summarised by the mean nMAE and mean R^2, obtained by averaging the per-target nMAE and R^2 values.

2.5 Multi-objective hyperparameter optimisation and stacking

Based on the baseline screening (Supplementary Methods 2.3), tree-based ensembles emerge as the most promising model family. For each selected family, we perform multi-objective hyperparameter optimisation using Optuna[19] with the NSGA-II algorithm[20]:

- Optimisation is carried out on the training split using 10-fold cross-validation.
- The filtered feature set from Supplementary Methods 2.2 is kept fixed within each Optuna study.
- Two scalar objectives are minimised simultaneously:
  a) the mean nMAE across the six targets (mean-nMAE objective);
  b) the worst-case (maximum) nMAE across the six targets (max-nMAE objective).

Each study produces a set of Pareto-optimal solutions in the (mean-nMAE, max-nMAE) plane. Selected Pareto solutions are retrained on the full training set and then evaluated once on the held-out test set.

We further refine the feature set using SHAP importance scores. For the candidate tree models, SHAP values are computed on the training split and aggregated across the targets to obtain a global importance ranking. We retain the global top-k features by mean | SHAP | and take the union with the top-10 features for each model, producing a compact high-importance feature subset. The NSGA-II optimisation described above is then repeated on this final feature set, and new Pareto solutions are selected under the same mean-nMAE and max-nMAE criteria.

In addition, we explored a two-level stacking scheme using a Ridge meta-learner[21]. For each base model, out-of-fold (OOF) predictions are generated on the training split and stacked as meta-features to train the Ridge regressor. At test time, base-model predictions are combined by the meta-learner. In our case, this stacking did not provide systematic improvements over the best single XGBoost model, which is therefore retained as the final surrogate.

2.6 SHAP-based interpretability analysis

The final surrogate is an XGBoost regressor wrapped in a MultiOutputRegressor, trained on the training split with the final reduced feature set. The model directly predicts the six DFT-level quantities per composition: $V_{\mathrm{ort/hex}}$, $m_{\mathrm{ort/hex}}$, $E_{\mathrm{ort/hex}}$. From these outputs we construct six derived targets of direct relevance to magnetocaloric performance:

- the hexagonal formation energy $E_{\mathrm{f}}^{\mathrm{hex}}$;
- the orthorhombic formation energy $E_{\mathrm{f}}^{\mathrm{ort}}$;
- the hexagonal magnetic moment m_{hex};
- the orthorhombic magnetic moment m_{ort};
- the structural energy difference $\Delta E_{\mathrm{str}} = E_{\mathrm{hex}} - E_{\mathrm{ort}}$;
- the relative volume change $\Delta V_{\mathrm{ratio}} = (V_{\mathrm{ort}} - V_{\mathrm{hex}})/V_{\mathrm{ort}}$.

SHAP values are computed on the training split using the exact tree-based explainer (TreeExplainer). For each target we obtain a SHAP value matrix of shape $(N_{\mathrm{train}}, N_{\mathrm{feat}})$. Global feature importance for that target is quantified by the mean absolute SHAP value per feature.

To derive a cross-target importance ranking, we average the mean$|\mathrm{SHAP}|$ scores over a selected subset of outputs (the most relevant DFT-level quantities and derived proxies),

yielding a single, target-agnostic ordering of the descriptors. This ranking underpins the global interpretation in the main text (Fig. 4b), while the underlying per-target SHAP distributions and feature–target dependence plots are provided in Fig. 4, Supplementary Figs. 7–8.

Supplementary Notes

1 Supplementary note on supercell consistency and DFT–ML comparison across three composition series

Supplementary Figs. 2–4 complement the main-text discussion of composition–property relationships and supercell consistency for the three series. By pooling results from multiple supercells and local configurations at the same nominal compositions, these figures provide a compact view of how supercell/configurational choices contribute to the dispersion of energetic, magnetic, and structural-related quantities, helping to contextualize the magnitude of this spread across composition and supporting the error assessment and trend interpretation in the main text.

Building on this, Supplementary Figs. 2–4 include a comparison with the XGB surrogate to highlight that a composition-based ML model can robustly extract the dominant composition-dependent trends even in the presence of DFT scatter. This indicates that the pointwise dispersion primarily reflects secondary effects associated with local-order and supercell approximations, while the surrogate offers a practical continuous reference for rapid screening and for prioritizing follow-up calculations or experiments, thereby improving exploration efficiency across all three series and broader compositional spaces.

2 Performance of the optimised RF, XGB, and RF+XGB stacking models

To push the limits of predictive accuracy, we evaluated a two-level stacking ensemble, utilizing a Ridge meta-learner to combine RF and XGB predictions. However, this added complexity yields no statistical gains over the gradient-boosted baseline. As shown in Fig. 3c, the comparison of predictive errors shows that the ensemble is nearly indistinguishable from the single XGB surrogate, with differences remaining below 0.01 in R^2 and below 0.001 in nMAE across all targets. This saturation in predictive performance suggests that the XGBoost architecture has effectively captured the learnable physics of the dataset, indicating that the remaining error likely stems from intrinsic variability in the underlying DFT data rather than model incapacity. Consequently, we retain the robust, interpretable XGBoost surrogate for all subsequent Shapley additive explanations (SHAP) analyses and predictions.

3 Additional SHAP interpretation of the XGB surrogate.

We provide the full target-level SHAP rankings [13,16], additional dependence/interaction plots, descriptor definitions, and robustness checks to support the main-text interpretation.

Fig. 4e–h and Supplementary Figs.7–8 provide target-resolved SHAP explanations for the XGBoost surrogate, combining SHAP summary plots that rank features by mean |SHAP| with SHAP dependence plots for the dominant descriptors (feature codes follow Table 3). Overall, phase-specific baseline properties (formation energies (E_f^{ort}, E_f^{hex}) and within-phase moments ($m_{\mathrm{ort}}, m_{\mathrm{hex}}$)) are largely governed by a small set of mean-type descriptors (F_{*_4}),

whereas inter-phase responses (ΔE_{str} and $\Delta V_{\mathrm{ratio}}$) are activated by dispersion-type descriptors (F_{*_5}) and exhibit pronounced non-linear, threshold-like behaviour.

Orthorhombic total moment (m_{ort})

For the orthorhombic moment m_{ort} (Fig. 4e), the most influential descriptor is MN_{ad} (a dispersion/inhomogeneity-type chemical descriptor), followed by the mean electronic descriptor $\bar{u}_T$, while $u_{T,ad}$ also ranks highly with an opposite tendency (high $u_{T,ad}$ generally contributing negatively). The dependence plot (Fig. 4g) shows a distinctly non-linear turn-on of SHAP (m_{ort}) with MN_{ad}, with a rapid rise around MN_{ad} ≈ 9–10 and a tendency to saturate at higher values; the colour feature MN_{mode} mainly modulates the amplitude.

Hexagonal total moment (m_{hex})

For m_{hex} (Supplementary Fig. 7c), the most influential descriptor is the mean number of unfilled total states ($\bar{u}_{\mathrm{T}}$, F_{18_4}), followed by the mean melting temperature ($\bar{T}_{\mathrm{melt}}$, F_{4_4}). The summary distribution indicates opposite directional effects: high $\bar{u}_{\mathrm{T}}$ yields predominantly positive SHAP contributions (enhancing m_{hex}), whereas high $\bar{T}_{\mathrm{melt}}$ tends to produce negative contributions (suppressing m_{hex}). The dependence plot (Supplementary Fig. 8c) reveals a largely smooth, nearly monotonic increase of SHAP (m_{hex}) with $\bar{u}_{\mathrm{T}}$, with a rapid transition from large negative to positive contributions and a tendency towards saturation at high $\bar{u}_{\mathrm{T}}$. The colour-coded feature $n_{d,ad}$ mainly modulates the spread but does not change the overall monotonic rise.

Formation energies (E_f^{ort} and E_f^{hex})

For the formation energy of the hexagonal phase E_f^{hex} (Supplementary Fig. 7a), the dominant descriptors are the mean atomic weight ($\bar{A}_w$, F_{3_4}) and the mean covalent radius ($\bar{r}_{cov}$, F_{7_4}). A consistent ordering is obtained for the orthorhombic formation energy E_f^{ort} (Supplementary Fig. 7b), again led by $\bar{A}_w$ and $\bar{r}_{cov}$. The dependence plots (Supplementary Fig. 8a, b) show an overall monotonic increase of SHAP with $\bar{A}_w$, featuring a noticeable "kink/step" across the mid-range of $\bar{A}_w$. Colour coding by σ_{MST} indicates that magnetic-moment dispersion mainly modulates the scatter rather than the monotonic direction. These trends suggest that phase formation energies are primarily controlled by mean chemistry/size/mass descriptors, with dispersion features acting as secondary modifiers.

Volume change ($\Delta V_{\mathrm{ratio}}$)

For $\Delta V_{\mathrm{ratio}}$ (Fig. 4f), the leading descriptor is the dispersion of per-atom magnetic moments (σ_{MST}), while the dispersion of total unfilled states ($u_{\mathrm{T},\ ad}$) ranks second. The summary plot indicates that high σ_{MST} is associated with positive SHAP values (increasing $\Delta V_{\mathrm{ratio}}$), whereas high $u_{\mathrm{T},ad}$ contributes mainly negative SHAP values (reducing $\Delta V_{\mathrm{ratio}}$), implying that the volume response is favoured by magnetic inhomogeneity (large σ_{mst}) combined with electronic homogeneity (small $u_{\mathrm{T},ad}$). The dependence plot (Fig. 4h) shows a clear threshold-like activation: SHAP ($\Delta V_{\mathrm{ratio}}$) remains weak up to σ_{MST} ≈ 0.75–0.8 and rises sharply once σ_{MST} exceeds this value. The colour coding by $\bar{r}_{\mathrm{cov}}$ primarily tunes the magnitude at high σ_{MST} without reversing the sign.

Structural energy difference (ΔE_{str})

For the inter-phase structural energy difference ΔE_{str} (Supplementary Fig. 7d), the top-ranked descriptor is again σ_{MST}, followed by covalent-radius dispersion (σ_{MST}). The dependence plot (Supplementary Fig. 8d) demonstrates a pronounced threshold: SHAP (ΔE_{str}) remains near zero at low σ_{MST} but increases rapidly once σ_{MST} exceeds ~0.8, indicating that large inter-phase energetic separation is activated only when the alloy exhibits sufficiently strong magnetic-moment mismatch.

Taken together, the SHAP analysis identifies two interpretable levers: mean descriptors (F_{*_4}) set phase-specific baselines (E_f and m within each phase), whereas dispersion descriptors (F_{*_5}) govern inter-phase contrasts and trigger threshold-like amplification of ΔE_{str} and $\Delta V_{\mathrm{ratio}}$.

Supplementary References

1. Togo, A., Chaput, L., Tadano, T. & Tanaka, I. Implementation strategies in phonopy and phono3py. *J. Phys. Condens. Matter* **35**, 353001 (2023).
2. Ong, S. P. *et al.* Python Materials Genomics (pymatgen): A robust, open-source python library for materials analysis. *Comput. Mater. Sci.* **68**, 314–319 (2013).
3. Togo, A., Shinohara, K. & Tanaka, I. Spglib: a software library for crystal symmetry search. *Sci. Technol. Adv. Mater. Methods* **4**, 2384822 (2024).
4. Shinohara, K., Togo, A. & Tanaka, I. Algorithms for magnetic symmetry operation search and identification of magnetic space group from magnetic crystal structure. *Acta Crystallogr. Sect. Found. Adv.* **79**, 390–398 (2023).
5. Kresse, G. & Hafner, J. Ab initio molecular dynamics for liquid metals. *Phys. Rev. B* **47**, 558–561 (1993).
6. Kresse, G. & Furthmüller, J. Efficient iterative schemes for ab initio total-energy calculations using a plane-wave basis set. *Phys. Rev. B* **54**, 11169–11186 (1996).
7. Blöchl, P. E. Projector augmented-wave method. *Phys. Rev. B* **50**, 17953–17979 (1994).
8. Kresse, G. & Joubert, D. From ultrasoft pseudopotentials to the projector augmented-wave method. *Phys. Rev. B* **59**, 1758–1775 (1999).

9. Perdew, J. P., Burke, K. & Ernzerhof, M. Generalized Gradient Approximation Made Simple. *Phys. Rev. Lett.* **77**, 3865–3868 (1996).

10. Wang, V., Xu, N., Liu, J.-C., Tang, G. & Geng, W.-T. VASPKIT: A user-friendly interface facilitating high-throughput computing and analysis using VASP code. *Comput. Phys. Commun.* **267**, 108033 (2021).

11. Ward, L. A general-purpose machine learning framework for predicting. *Npj Comput. Mater.* (2016).

12. Pedregosa, F. *et al.* Scikit-learn: Machine Learning in Python. *J Mach Learn Res* **12**, 2825–2830 (2011).

13. Lundberg, S. M. *et al.* From local explanations to global understanding with explainable AI for trees. *Nat. Mach. Intell.* **2**, 56–67 (2020).

14. Li, W. Mutual information functions versus correlation functions. *J. Stat. Phys.* **60**, 823–837 (1990).

15. Ross B. C. Mutual Information between Discrete and Continuous Data Sets. *PLOS ONE* **9**, e87357 (2014).

16. Lundberg, S. M. & Lee, S.-I. A unified approach to interpreting model predictions. in *Proceedings of the 31st International Conference on Neural Information Processing Systems* 4768–4777 (Curran Associates Inc., Red Hook, NY, USA, 2017).

17. Peng, H., Long, F. & Ding, C. Feature Selection Based on Mutual Information: Criteria of Max-Dependency, Max-Relevance, and Min-Redundancy. *IEEE Trans Pattern Anal Mach Intell* **27**, 1226–1238 (2005).

18. Chen, T. & Guestrin, C. XGBoost: A Scalable Tree Boosting System. in *Proceedings of the 22nd ACM SIGKDD International Conference on Knowledge Discovery and Data*

Mining 785–794 (ACM, San Francisco California USA, 2016). doi:10.1145/2939672.2939785.

19. Akiba, T., Sano, S., Yanase, T., Ohta, T. & Koyama, M. Optuna: A Next-generation Hyperparameter Optimization Framework. in *Proceedings of the 25th ACM SIGKDD International Conference on Knowledge Discovery & Data Mining* 2623–2631 (Association for Computing Machinery, New York, NY, USA, 2019). doi:10.1145/3292500.3330701.
20. Deb, K., Pratap, A., Agarwal, S. & Meyarivan, T. A fast and elitist multiobjective genetic algorithm: NSGA-II. *IEEE Trans. Evol. Comput.* **6**, 182–197 (2002).
21. Wolpert, D. H. Stacked generalization. *Neural Netw.* **5**, 241–259 (1992).
