# Supplementary material for: DFT-based machine-learning for the rational design of magnetocaloric high-entropy alloys
Source: Nat Commun. 2026 Aug 25;17:10685. doi: 10.1038/s41467-026-77123-w (PMC13654988; doi:10.1038/s41467-026-77123-w)
Supplement: Supplementary file 2 [file 41467_2026_77123_MOESM2_ESM.pdf]

nature portfolio

# Peer Review File

# DFT-based Machine-Learning for the Rational Design of Magnetocaloric High-Entropy Alloys

Corresponding Author: Professor Victorino Franco

**This file contains all reviewer reports in order by version, followed by all author rebuttals in order by version.**

Version 0:

Reviewer comments:

Reviewer #1

(Remarks to the Author)
In this manuscript, the authors create a computationally efficient framework for screening and predicting magnetocaloric high entropy alloys which also results in physically meaningful design criteria for new materials. By and large, this manuscript is well written, flows logically, has impactful graphics, and conveys very interesting and important results for this field. I believe it is almost publication-ready as-is, but have a few minor comments about things to address or fix that will fully polish this manuscript.

- Typo line 65--VCAs supposed to be VLAs?--this term also doesn't appear again so doesn't need to be abbreviated
- Fig. 1 "bottom" labelling--needs to be reworded to make it clear the second sentence is contrasting with first sentence, right now is like an incomplete statement
- This is just a comment based on personal preference, but I think a more logical abbreviation for SHapley Additive exPlanations would be "SHapley ADditive Explanations" or SHADE, but perhaps there is a reason why the authors chose SHAP instead, like avoiding overlap with other acronyms with which I'm not familiar
- Figure 3 appears a little low res compared to the others--is it possible to improve this?
- Fig3c--text says this is a plot showing the error profile for each term; can this be made more clear in the caption? From the caption it seems like those are predictions of the values themselves in which case the smaller polygon wouldn't be the "better" one
- Some labels and axis numbering in figures is a bit small, particularly the legends in Fig. 2c and I, legends corresponding to Fig. 3b-d, variable labels throughout Fig. 4 and 5, and S6 and S7
- Something is compiling oddly for the captions of S6 and S7 in the supplemental file--there is a vertical caption for each figure separate from the text caption corresponding to both figures
- Can the authors speak to why the models might be fairly consistently under-predicting the unit cell volume compared to the experimental values (from Fig. 3d/table 2) Though it's within a fairly close margin the fact that almost all of the data falls below the prediction line suggests this isn't just due to statistical variation

Reviewer #3

(Remarks to the Author)
The subject of the work and the approach taken as well as the results present, are very interesting and innovative. I can say that it definitely captured my interest, and for that I apologize in advance to the authors by my enthusiasm in making suggestions.

In this work, the authors started by determining, through a selection of compounds, the smallest unit – the minimal supercell - that could be used to describe successfully main physical properties of the MM’X materials family. The conclusion, that the HEA’s macroscocopic properties are not defined by the long-range configurational randomness aligns with the perception that disorder (chemical, structural, magnetic…) often imposes strong limitations in effects from long-range interactions. Defined the minimal supercell, the basis for a balanced resources-accuracy throughput calculations (in another work from the authors) is established, and the results used to train different surrogate models. The authors highlight the performance of model trained with the XGBoost algorithm, and show its success in predict accurately the electronic total energies, volumes

and predict adequately the average magnetic moment. To my knowledge, the approach sounds sensible, and from similar works on machine learning methods and HEA by the inclusion of the magnetic moments as a feature in the model, and by the use of supercells for the descriptions the systems' disorder over the use of CPA. After verification of the reliability of the model the authors proceed to what I consider the most significant and innovative component of the work, the extraction of physical information from the surrogate model, to identify which physical descriptor play an actual role in enhancing/(limiting) the magnetocaloric performance of the MM'X alloys. These approach and respective outcomes are groundbreaking in the sense of guiding the search for new magnetocaloric materials of this class, and in the sense of inspiring the application of a similar approach in other class of magnetocaloric materials (e.g Heusler alloys).

Overall in manuscript: the results and conclusions presented in the manuscript are relevant for the field of magnetocaloric materials and material science. The authors do an extensive statistical analysis at each one of the stages, before drawing observations of the results, providing them an adequate support for the conclusions. Moreover, the methodology followed is solid, well-founded and has a very appreciated concern for the reproducibility of the results. A small note on the later can be done – the authors rely on a curated dataset from a previously published study (Reference 52), which is currently unpublished. While the use of such data is not inherently prohibitive, its lack of public availability at the time of submission limits the ability to scrutinize or replicate the results. It is hoped that the reference can be updated once the dataset is published, ensuring full transparency and reproducibility.

I have just a couple of minor observations to mention:

a) In the main paragraph of the introduction, the omission of other promising families of magnetocaloric materials —such as Heusler alloys , $La_{0.7}Ca_{0.3}MnO_3$, or $LaFe_{13-x}Si_x$—may suggest to the reader that only HEAs and rare-earth-based compounds are potential candidates for real world applications. A brief mention or recognition of these additional classes (as discussed, for example, in Gottschall et al., Making a Cool Choice: The Materials Library of Magnetic Refrigeration) could provide useful context for the general audience . This would also allow for a clearer motivation of HEAs' advantages, particularly in terms of their potential mechanical properties (e.g., hardness, corrosion and oxidation resistance), which enhance their practical applicability."

b) I found the treatment of ferrimagnetic solutions in the DFT calculations to be a bit unclear. While the caption of Figure 2 mentions that such cases were excluded from the subsequent analysis, it is not explicitly stated whether this exclusion applies to the entire study (e.g., whether only ferromagnetic solutions were used to train the surrogate model) or if it is limited to the analysis shown in Figures 2a–l. Given the critical role that magnetic order plays in magnetocaloric materials, I strongly recommend clarifying this point in the main text – or even extending the discussion.

c) The manuscript faces the inherent challenge of balancing depth and accessibility. Given the volume of work and information presented, the text is dense, making it difficult for a general audience to engage with the content. I must admit that, at times, the text—and even the analysis of the figures—was not immediately clear to me. While the technical approach, including the SHAP analysis, is justified, it may reduce accessibility for non-expert readers. I believe this can be improved to ensure the article is understandable to a broader audience, enabling them to fully appreciate the results and methodology. Below, I list a few suggestions:
> In the section titled "Efficient DFT Methodology for HEAs: Supercell Selection and Atomic Ordering," integrating the first paragraph of the supplement could improve clarity. Additionally, providing a brief description (e.g., the number of atoms per cell) or an illustration of the primitive cells would be helpful. It might also be useful to include a note—even if it can be inferred—that the 1×1×1 "supercell" could not accommodate all the compositions examined in the study.

> I believe the discussion of the Random Forest algorithm, its comparison with XGBoost, and the performance results (lines 237–283, including Figures 3b and 3c) deviates slightly from the manuscript's main narrative. These details could be moved to the supplement, which would help streamline the main text and reduce its technical density.

> In the discussion of the SHAP analysis results (Figures 4d–h), a more guided explanation of how to interpret these results and their significance would greatly benefit the reader.

Besides this, driven by interest of the results I suggest the following questions to be discussed/extended in the manuscript if the authors see pertinent:

> Why the results for the MnNi-FeSi series, using the 1x1x1 "supercell", has such dispersion in comparison with the other results in in Figure 2.g-l ?

> Does the surrogate model's predicted total energy have sufficient accuracy (within the established 1–2 meV/atom uncertainty) to reliably compare the orthorhombic and hexagonal phases and allow to identify compositions where the hexagonal phase is the ground state and excluding them as candidates for magnetostructural transitions. It would be insightful to include a confusion matrix in the supplement to address this question.

> Could you please elaborate on why the magnetic moment of the hexagonal phase was chosen as the critical target for the magnetocaloric effect, rather than the magnetic moment of the orthorhombic phase? More specifically, why not focus on the

difference between the two?

Minor Suggestions for Clarity and Presentation:

>There appears to be a small typo on line 194: "Figure 2j–l" should likely be "Figure 2g–l."

>The parity plots in Figure 2d–f and Figure 3d–g would benefit from a square aspect ratio between the x- and y-axes for better visual consistency.

>In Figure 2, the colour legend is repeated in subplots a and b. Given that the legend is consistent across all subplots, a single, unified legend above or below the figure could improve cohesion.

>In Figure 1, adding a downward-pointing arrow in the subset illustration would better highlight the flow of the traditional screening process, clarifying how the authors' approach differs. Moreover, the text boxes in Figure 1 could be repositioned, and the blue text colour reconsidered for improved readability.

>In Figure 3b, the bright yellow legend text is difficult to discern.

>The Index column and Feature code in Table 3 may not be essential for the main text. Consider moving the current version to the supplement and simplifying the table in the main text.

I hope these suggestions are received as constructive points to further strengthen the authors' already impressive and innovative work. I look forward to seeing the revised manuscript.

Version 1:

Reviewer comments:

Reviewer #3

(Remarks to the Author)
The authors have addressed the reviewers' comments and suggestions, implementing changes that streamline the article and expand its reach. They have satisfactorily answered the questions raised and expanded the text so that readers with similar questions can find the answers directly. Additionally, the authors have broadened their discussion on the analysis of both structural phases, completing their study and aligning it with common practices in the field of magnetocaloric materials. Overall, I believe the article will be of great interest to the scientific community, particularly in the caloric materials field, and is ready for publication.

A few minor typos and suggestions that the authors might want to consider:
• Figure 2: While it is true that the structures are not symmetrically equivalent and may show different trends, the main text groups the supercells into 2x and 4x categories. To streamline the figure (while retaining the current version in the supplement), the authors could group the data for the 1×1×2, 1×2×1, and 2×1×1 supercells under "2x supercell," and similarly for the 4x case.
• Line 163 (page 5): For readers unfamiliar with these structures, it would be helpful to specify the corresponding supercell size, e.g., change "(24 atoms)" to "(24 atoms, 2x supercell)" or provide this information earlier, such as: "supercells (1×1×1 (12 atoms), 2×1×1, 1×2×1, 1×1×2 (24 atoms), 2×2×1, 1×2×2, 2×1×2 (48 atoms))".
• Table 3: For consistency, consider using the full name for "avg_dev."
• Line 426 (page 15): Replace "Note:" with "Note that" to improve text flow.

In the supplement:
• Supplementary lines 223 and 225 (page 8): Change "hexagonal" to "orthorhombic."

(Remarks on code availability)

**Open Access** This Peer Review File is licensed under a Creative Commons Attribution 4.0 International License, which permits use, sharing, adaptation, distribution and reproduction in any medium or format, as long as you give appropriate credit to the original author(s) and the source, provide a link to the Creative Commons license, and indicate if changes were made.
In cases where reviewers are anonymous, credit should be given to 'Anonymous Referee' and the source.
The images or other third party material in this Peer Review File are included in the article's Creative Commons license, unless indicated otherwise in a credit line to the material. If material is not included in the article's Creative Commons license and your intended use is not permitted by statutory regulation or exceeds the permitted use, you will need to obtain permission directly from the copyright holder.
To view a copy of this license, visit https://creativecommons.org/licenses/by/4.0/

# Response Letter

**Dear Editor and Reviewers,**

We thank the Editor and the Reviewers for their constructive comments. We have revised the manuscript accordingly, and all changes are highlighted in yellow in the revised manuscript. Below, we provide a point-by-point response to each comment.

## Reviewer #1

**In this manuscript, the authors create a computationally efficient framework for screening and predicting magnetocaloric high entropy alloys which also results in physically meaningful design criteria for new materials. By and large, this manuscript is well written, flows logically, has impactful graphics, and conveys very interesting and important results for this field. I believe it is almost publication-ready as-is, but have a few minor comments about things to address or fix that will fully polish this manuscript.**

### Comment 1. Typo line 65--VCAs supposed to be VLAs?--this term also does not appear again so does not need to be abbreviated."

**Response:** Thank you for raising this point. We have made sure to use the corrected virtual crystal approximation (VCA) consistently throughout the revised manuscript as it is recognised as the standard abbreviation accepted in the literature:

*Main text, Introduction, line 69:*

To model chemically disordered, nearly random atomic distributions in HEAs within periodic boundary conditions, researchers usually turn to approximate methods, such as special quasirandom structures (SQS) [24–26], the coherent potential approximation (CPA) [27–29], virtual crystal approximation (VCA) [30,31] as well as statistical averaging approaches [32,33].

### Comment 2. "Fig. 1 "bottom" labelling--needs to be reworded to make it clear the second sentence is contrasting with first sentence, right now is like an incomplete statement"

**Response:** We have revised the text labels in **Figure 1** to clearly differentiate between the inset and main panels, as well as updated the accompanying text for clarity, following the suggestions from all reviewers.

*Main text, **Figure 1**, line 107:*

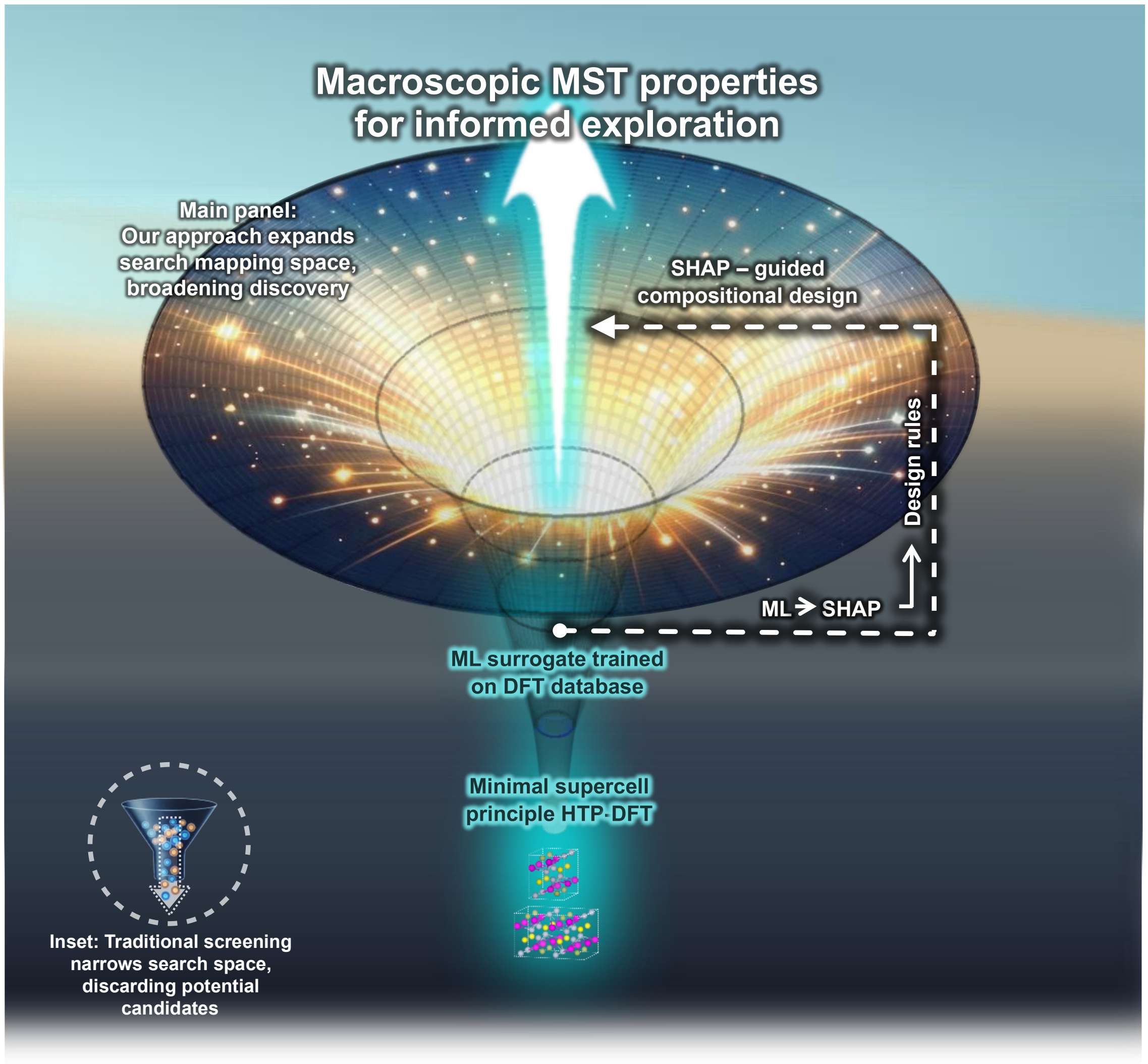


**Figure 1. Inverting the screening paradigm: from limited local identification to global design space mapping. Main panel:** The schematic illustrates the workflow effectively inverting the conventional screening funnel. The process begins at the narrow base, where the "minimal-supercell principle" anchors the search of our work, identifying a wider scope of promising compositions, more fully utilising the vast HEA space. Unlike traditional screening methods, computationally confined to identifying a narrow slice of the configurational space for promising compositions (represented in the **inset panel**). **Middle:** High-throughput DFT (HTP-DFT) calculations on the "minimal-supercell principle" capture essential local atomic environments for training dataset with first-principles accuracy, which feeds a physics-based Machine Learning (ML) surrogate. This engine acts as a "lens" to project local insights upward, breaking through the computational ceiling of standard techniques. In the expanded upper region ("the sky"), the model extrapolates to map the stability-performance Pareto frontier across the vast, previously inaccessible compositional landscape. Outcome: SHapley Additive exPlanations (SHAP) analysis decodes this map, extracting interpretable design rules, such as magnetic moment dispersion, to guide the rational discovery of optimised magnetocaloric materials with macroscopic magnetostructural (MST) behaviour.

**Comment 3. "This is just a comment based on personal preference, but I think a more logical abbreviation for SHapley Additive exPlanations would be "SHapley ADditive Explanations" or SHADE, but perhaps there is a reason why the authors chose SHAP instead, like avoiding overlap with other acronyms with which I'm not familiar."**

**Response:** We appreciate the reviewer for bringing this up and sharing this point of view. Given that SHapley Additive exPlanations is commonly referred to as SHAP in both the literature and the machine-learning community, we opted for this familiar abbreviation to ensure consistency with established norms.

**Comment 4. "Figure 3 appears a little low res compared to the others--is it possible to improve this?"**

**Response:** We have replaced a higher-resolution version of **Figure 3** in the revised manuscript. In addition, we have provided SVG versions of all figures to the editor to ensure high-quality rendering.

*Main text, **Figure 3**, line 262:*

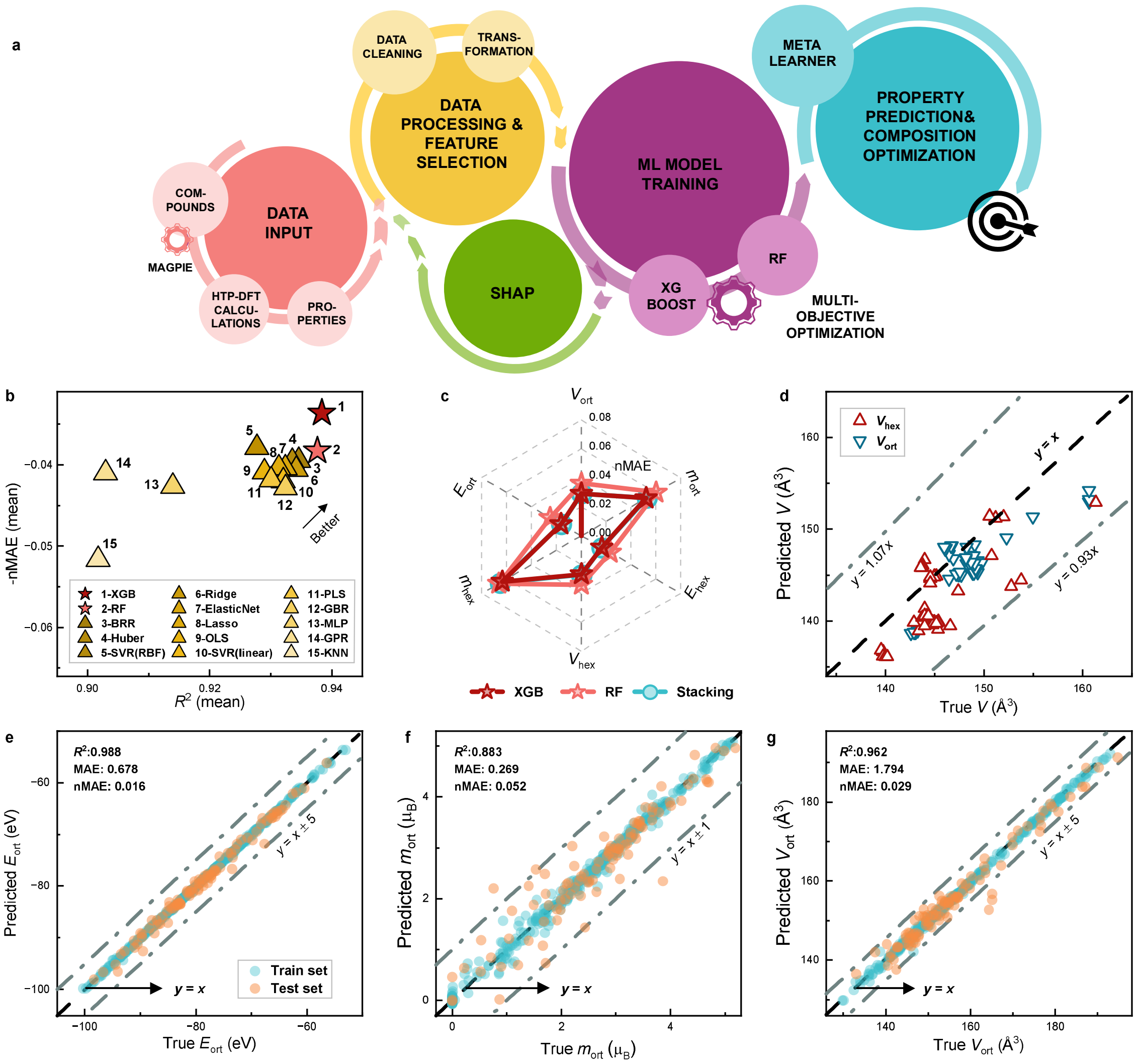


**Figure 3. Machine-Learning Surrogate Performance and Validation. a,** Schematic diagram of the ML workflow linking the HTP-DFT dataset to the final multi-output surrogate model. **b,** Model screening: trade-off between nMAE and mean $R^2$ for various ML algorithms. It highlights XGB and RF

as the best performers (star symbols). **c,** Radar plot of the test nMAE for the six target properties (*E*, *V*, and *m* for both structures) across different models. Smaller polygons indicate lower overall error and thus better predictive performance, with XGB showing the best overall performance. **d,** Comparison between ML-predicted vs. experimental/literature unit-cell volumes for various hexagonal (hex) and orthorhombic (ort) MM'X. The solid black line (*y* = *x*) represents perfect agreement, and dashed lines indicate a ±7% error band. Experimental values are taken from in-house work and literature [12,51,52,60,61,49,62–72]. **e–g,** Detailed parity plots between ML predictions and DFT calculations for $E_{\mathrm{ort}}$, $m_{\mathrm{ort}}$ and $V_{\mathrm{ort}}$, respectively. The tight grouping around *y* = *x* (black dashed line) demonstrates high accuracy.

**Comment 5. "Fig3c--text says this is a plot showing the error profile for each term; can this be made more clear in the caption? From the caption it seems like those are predictions of the values themselves in which case the smaller polygon wouldn't be the "better" one."**

**Response:** We have revised the caption to clarify that the radar plot presents the test nMAE across the six target properties, so that a smaller polygon indicates lower overall error and better predictive performance. The revised **Figure 3** and caption incorporating all reviewers' comments can be referred to our response to *Reviewer 1, comment 4*.

**Comment 6. "Some labels and axis numbering in figures is a bit small, particularly the legends in Fig. 2c and l, legends corresponding to Fig. 3b-d, variable labels throughout Fig. 4 and 5, and S6 and S7."**

**Response:** Adhering to the journal's requirements, we have standardised the figure formats, which include the font size, tick labels, and variable labels throughout the main text and Supplementary Information.

*Main text, **Figure 2**, line 162:*

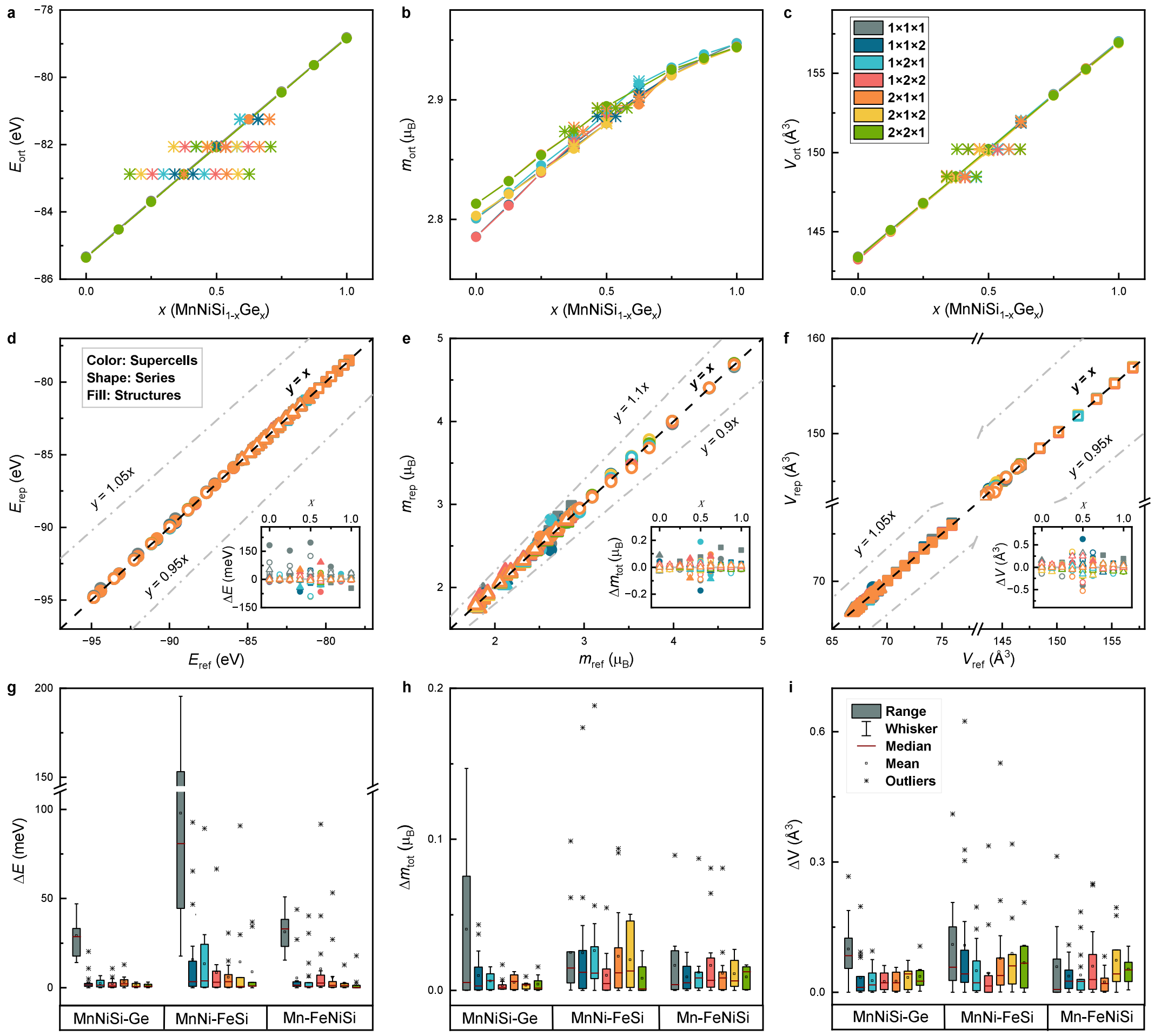


**Figure 2. Composition–property relationships and supercell consistency. a,** Compositional dependence of energy, *E*, **b,** magnetic moment, *m*, and **c,** volume, *V*, for the orthorhombic structure in $MnNiSi_{1-x}Ge_x$. Colours distinguish supercells; solid lines connect representative configurations, while symbols indicate non-representative configurations at the same x; a small horizontal displacement is added solely for visual separation. Occasional spread observed near intermediate Mn–Fe substitutions, consistent with variations of Mn/Fe moments. *m* and V display a somewhat broader yet still acceptable spread. Notably, the $MnNi_{0.375}Fe_{0.625}Si$ composition in the 2×1×2 hexagonal supercell (**Figure S3d, f** green line/points) stabilises in a ferrimagnetic configuration. This behaviour can occasionally arise in DFT calculations, resulting in *m* and *V* values that may not be appropriate for that specific point[33]. Therefore, we exclude these from the supercell-consistency analysis to ensure reliability of our results. **d–f,** Parity plots comparing representative values ($P_{rep}$) against the per-composition reference values ($P_{ref}$) for energy, magnetic moment, and volume. The tight clustering along the 1:1 line confirms that minimal supercells introduce negligible systematic bias. **g–i,** Box plots summarising the deviation magnitude, $\Delta P = P_{rep} - P_{ref}$, across all series, demonstrating that property predictions are robust against supercell choice. Boxes show interquartile range (IQR), centre line is the median, whiskers are 1.5×IQR, and outliers are represented by scatter points.

The updated **Figure 3** is provided above in *Reviewer 1, comment 4*.

Main text, **Figure 4**, line 350-359:

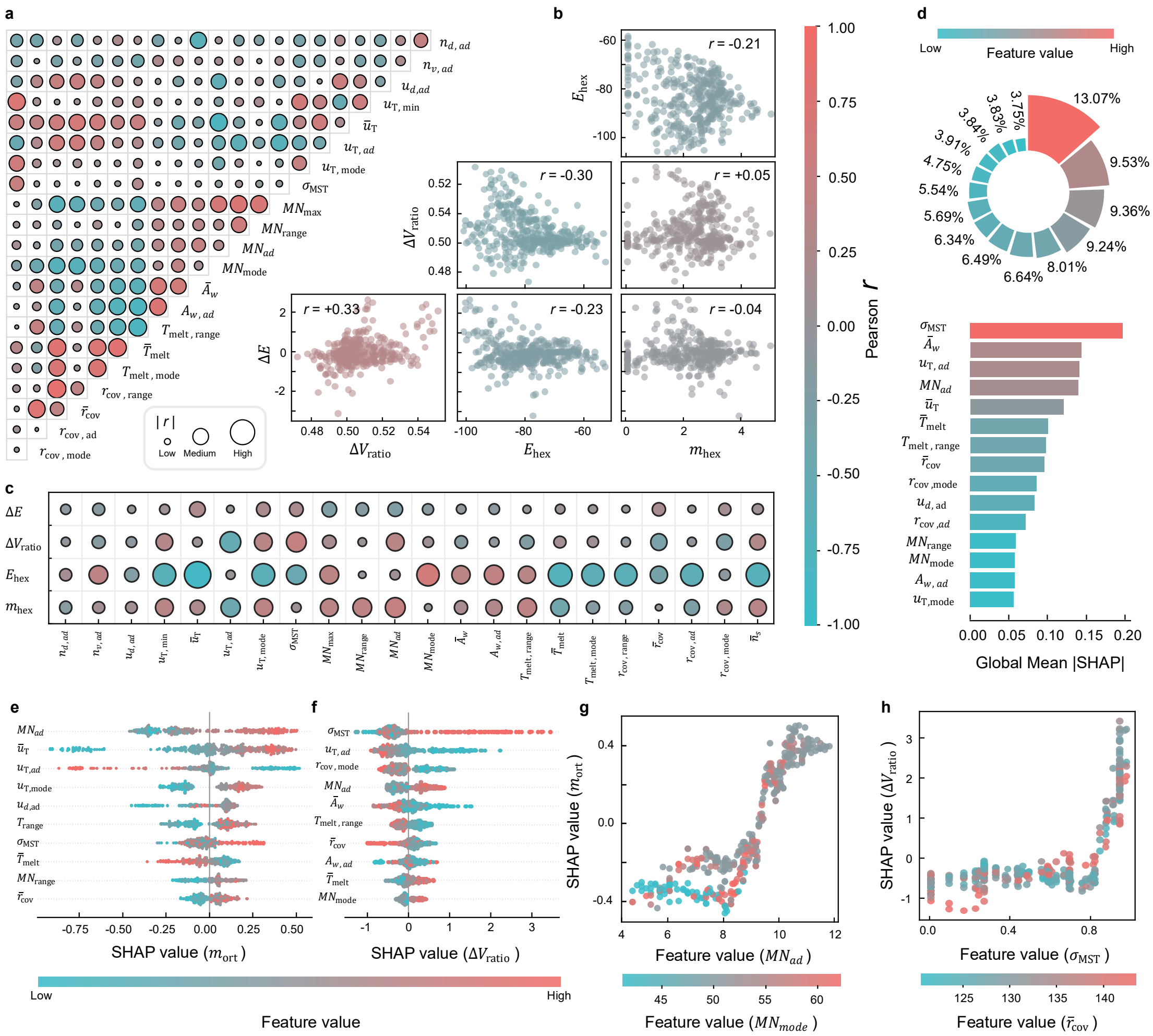


**Figure 4. SHAP interpretation of the XGB surrogate.** Pearson-correlation matrix between the selected Magpie descriptors and the key magnetocaloric proxies: **a**, feature–feature correlations, **b,** proxy–proxy correlations, and **c,** correlations between features and proxies. **d,** Global SHAP feature importance averaged over six key targets ($E_f^{ort}$, $E_f^{hex}$, $m_{ort}$, $m_{hex}$, $\Delta E_{str}$, $\Delta V_{ratio}$), (Upper: normalised feature-importance share (%). Lower: global mean |SHAP| values used to rank features), identifying atomic mass, covalent radius, and unfilled states as dominant drivers. **e–f,** SHAP summary plots for $m_{ort}$ and $\Delta V_{ratio}$, ranking features by their impact. Dot colours indicate the feature value (blue: low, red: high). **g–h,** SHAP dependence plots illustrating the non-linear impact of the most influential descriptors on $m_{ort}$ and $\Delta V_{ratio}$. Feature codes follow **Table 3**.

Main text, **Figure 5**, line 422:

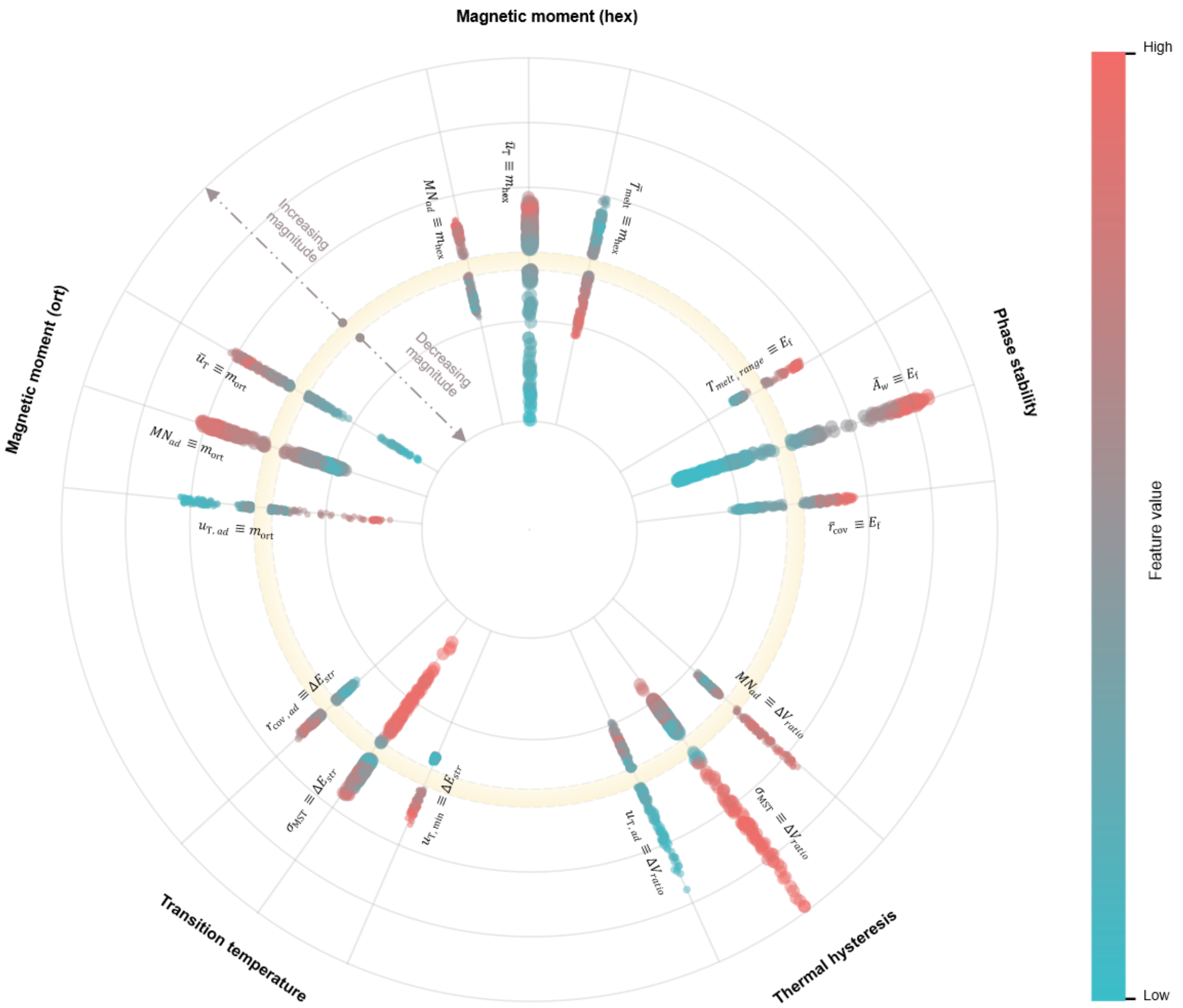


**Figure 5. The stability-performance trade-off driven by synergistic disorder.** Radial "guide map" connecting magnetocaloric-relevant performance metrics (outer labels) to the most actionable composition descriptors learned by the SHAP analysis. Each spoke corresponds to one experimentally relevant objective (e.g., magnetic moment of hex/ort phase, phase stability, transition temperature, and thermal hysteresis). For each objective, the top-ranked descriptors are plotted as three jittered point clouds: the primary descriptor is centred on the spoke, while the descriptors (right: second; left: third) are shown with small angular offsets. Each dot represents one composition in the training set; the radial coordinate encodes the signed, target-normalised SHAP value (distance from the zero ring indicates contribution magnitude; inner/outer sides correspond to negative/positive contributions). Marker colour indicates the descriptor value (low to high, normalised per descriptor).

Supplementary Information, **Figure S6**, line 377-381:

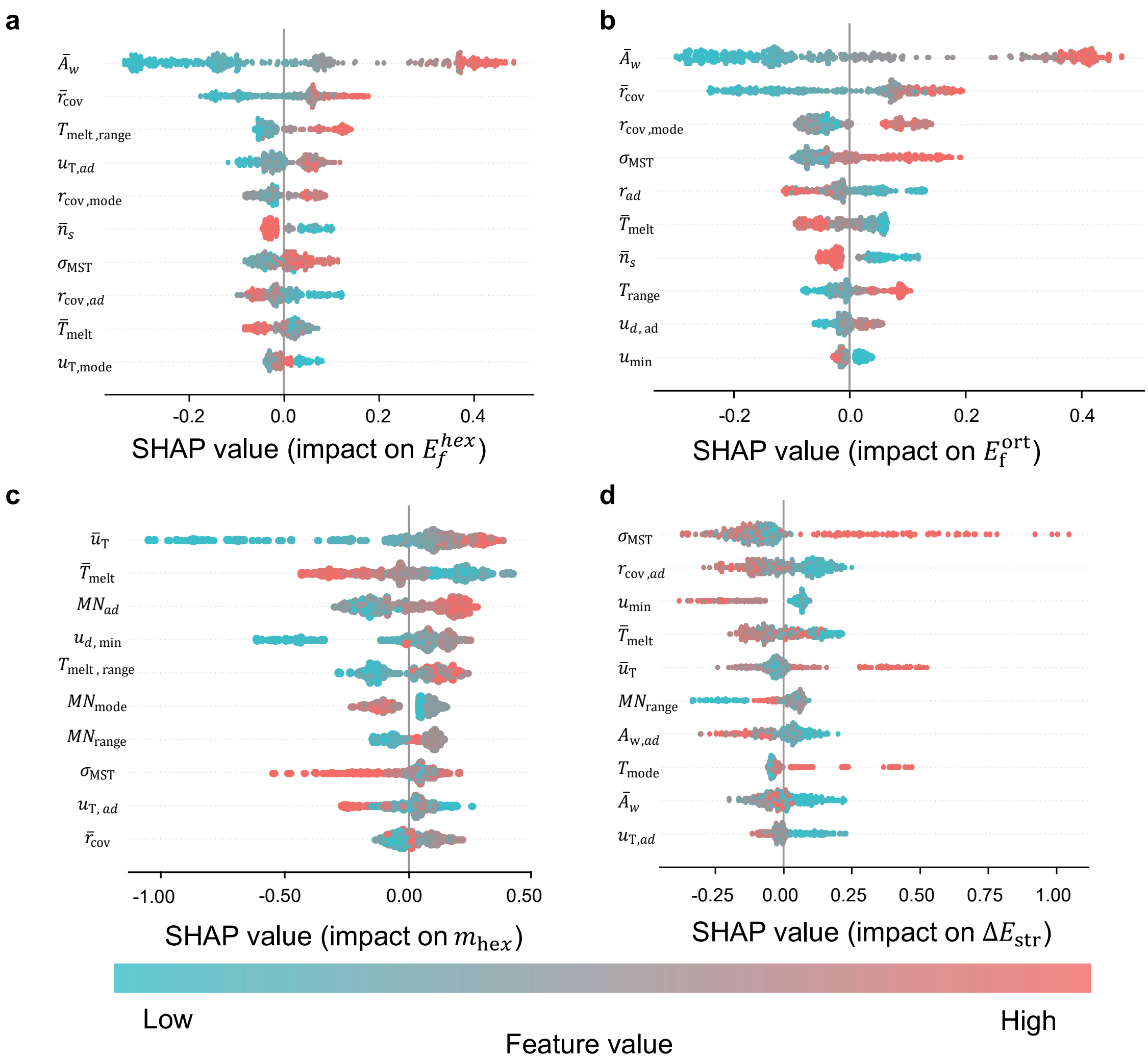


**Figure S6.** SHAP interpretation of the XGB surrogate. SHAP summary plots for (a) $E_f^{hex}$, (b) $E_f^{ort}$, (c) $m_{hex}$. and (d) $\Delta E_{str}$. Features are ordered by decreasing importance (mean | SHAP |); each point corresponds to one sample, and its colour encodes the feature value (blue: low, red: high). Feature codes follow **Table 3**.

Supplementary Information, **Figure S7**, line 382-385:

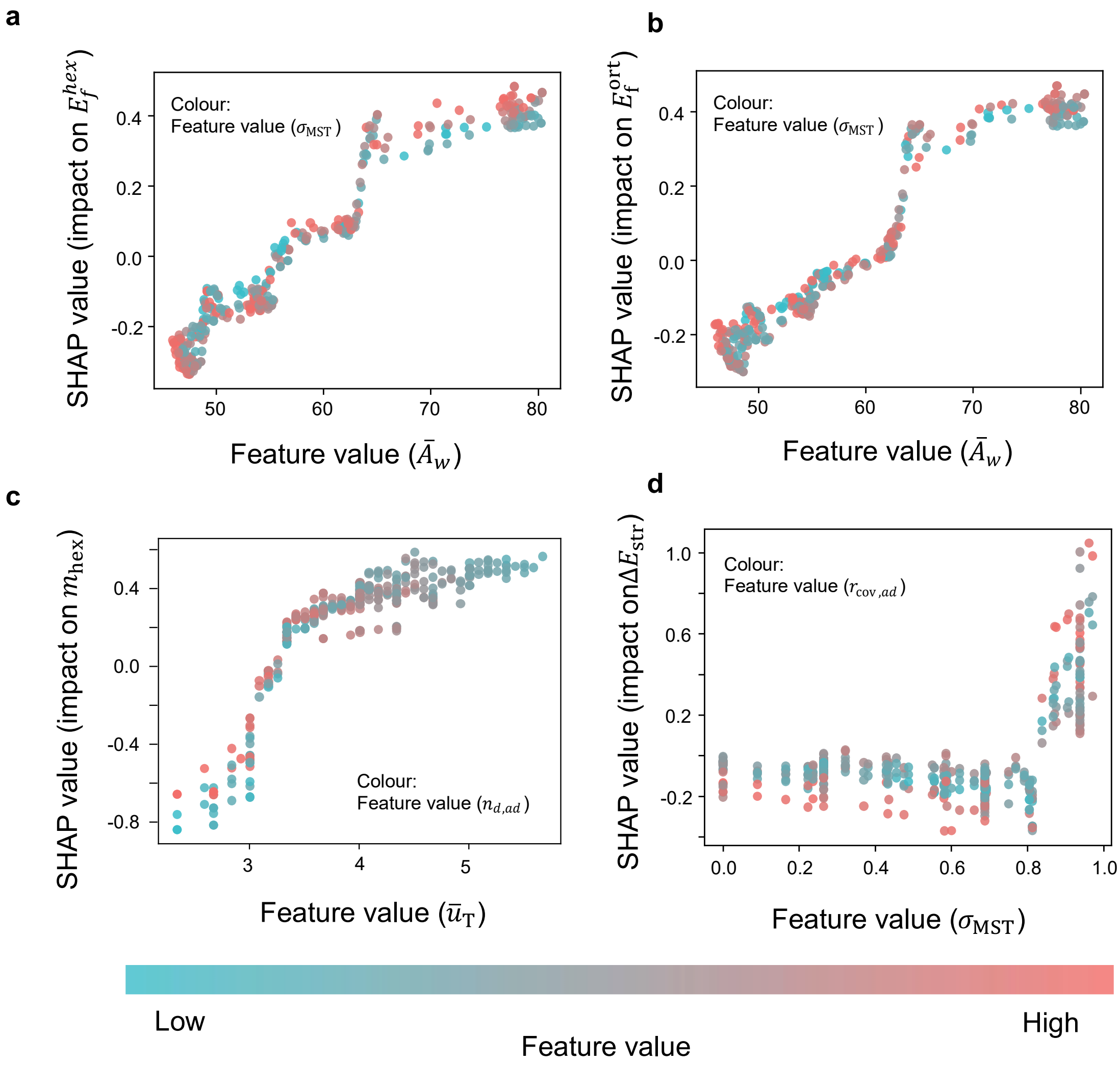


**Figure S7.** SHAP interpretation of the XGB surrogate. SHAP dependence plots for key descriptors: (a) $\bar{A}_w$ versus $E_f^{hex}$, (b) $\bar{A}_w$ versus $E_f^{ort}$, (d) $\bar{u}_T$ versus $m_{hex}$ and (d) $\sigma_{MST}$ versus $\Delta E_{str}$. Feature codes follow **Table 3**.

**Comment 7. "Something is compiling oddly for the captions of S6 and S7 in the supplemental file--there is a vertical caption for each figure separate from the text caption corresponding to both figures."**

**Response:** We have corrected the panel labels a and b in **Figures S6** and **S7** in the revised Supplementary Information. Please refer to our response to *Reviewer 1, comment 6,* for the revised **Figures S6** and **S7** incorporating all comments from reviewers.

**Comment 8. “Can the authors speak to why the models might be fairly consistently under-predicting the unit cell volume compared to the experimental values (from Fig. 3d/table 2) Though it's within a fairly close margin the fact that almost all of the data falls below the prediction line suggests this isn't just due to statistical variation.”**

**Response:** In the revised manuscript, we have added a brief discussion to address this point and explain that the observed bias likely reflects the difference between the DFT-relaxed 0 K structures used for model training and the finite-temperature conditions of experimental measurements.

*Main text, line 289-293:*

“As detailed in Table 2, the surrogate captures the experimentally reported lattice parameters (as well as in-house work) with high accuracy, despite a slight systematic underestimation of the unit-cell volume. This likely reflects the difference between the DFT-relaxed 0 K structures used for training and the finite-temperature conditions of experimental measurements.”

## Reviewer #3

**The subject of the work and the approach taken as well as the results present, are very interesting and innovative. I can say that it definitely captured my interest, and for that I apologize in advance to the authors by my enthusiasm in making suggestions.**

**In this work, the authors started by determining, through a selection of compounds, the smallest unit – the minimal supercell - that could be used to describe successfully main physical properties of the MM'X materials family. The conclusion, that the HEA's macroscocopic properties are not defined by the long-range configurational randomness aligns with the perception that disorder (chemical, structural, magnetic...) often imposes strong limitations in effects from long-range interactions. Defined the minimal supercell, the basis for a balanced resources-accuracy throughput calculations (in another work from the authors) is established, and the results used to train different surrogate models. The authors highlight the performance of model trained with the XGBoost algorithm, and show its success in predict accurately the electronic total energies, volumes and predict adequately the average magnetic moment. To my knowledge, the approach sounds sensible, and from similar works on machine learning methods and HEA by the inclusion of the magnetic moments as a feature in the model, and by the use of supercells for the descriptions the systems' disorder over the use of CPA. After verification of the reliability of the model the authors proceed to what I consider the most significant and innovative component of the work, the extraction of physical information from the surrogate model, to identify which physical descriptor play an actual role in enhancing/(limiting) the magnetocaloric performance of the MM'X alloys. These approach and respective outcomes are groundbreaking in the sense of guiding the search for new magnetocaloric materials of this class, and in the sense of inspiring the application of a similar approach in other class of magnetocaloric materials (e.g Heusler alloys).**

**Overall in manuscript: the results and conclusions presented in the manuscript are relevant for the field of magnetocaloric materials and material science. The authors do an extensive statistical analysis at each one of the stages, before drawing observations of the results, providing them an adequate support for the conclusions. Moreover, the methodology followed is solid, well-founded and has a very appreciated concern for the reproducibility of the results. A small note on the later can be done – the authors rely on a curated dataset from a previously published study (Reference 52), which is currently unpublished. While the use of such data is not inherently prohibitive, its lack of public availability at the time of submission limits the ability to scrutinize or replicate the results. It is hoped that the reference can be updated once the dataset is published, ensuring full transparency and reproducibility.**

### Comment 1. (From "Overall in manuscript") "A small note on the later can be done – the authors rely on a curated dataset from a previously published study (Reference 52), which is currently unpublished. While the use of such data is not inherently prohibitive, its lack of public availability at the time of submission limits the ability to scrutinize or replicate the results. It is hoped that the reference can be updated once the dataset is published, ensuring full transparency and reproducibility."

**Response:** We have addressed this concern by making the curated dataset available through idUS. The revised manuscript has been updated to include the corresponding data availability

information, so that the dataset used in this work can be accessed. We have also revised the relevant reference/data statement accordingly to ensure transparency and reproducibility:

*Main text, **Data Availability**, line 593-597:*

The curated dataset generated and analysed in this study was derived from high-throughput DFT calculations performed by the authors as part of a related study in preparation and has been deposited in idUS at https://doi.org/10.12795/11441/186086. Source Data are provided with this paper.

*Main text, **Code Availability**, line 598-603:*

The custom code used for data processing, machine-learning model training, model evaluation, SHAP analysis and figure generation is available on GitHub at https://github.com/zhecui592/DFT-ML-Magnetocaloric-HEAs and has been archived in idUS at https://doi.org/10.12795/11441/186089. The code includes documentation and example input/output files to support reproducibility.

**Comment 2. "In the main paragraph of the introduction, the omission of other promising families of magnetocaloric materials —such as Heusler alloys, La0.7Ca0.3MnO3, or LaFe13-xSix—may suggest to the reader that only HEAs and rare-earth-based compounds are potential candidates for real world applications. A brief mention or recognition of these additional classes (as discussed, for example, in Gottschall et al., Making a Cool Choice: The Materials Library of Magnetic Refrigeration) could provide useful context for the general audience. This would also allow for a clearer motivation of HEAs' advantages, particularly in terms of their potential mechanical properties (e.g., hardness, corrosion and oxidation resistance), which enhance their practical applicability."**

**Response:** In the revised manuscript, we have included a sentence acknowledging several key magnetocaloric families, in line with the broader materials perspective by *Gottschall et al*. In addition, we have refined the manuscript text to effectively highlight the motivation behind studying HEAs. We emphasize not only their potential as RE-free magnetocaloric materials, but also their possible benefits in terms of mechanical robustness and corrosion/oxidation resistance, which may further enhance their practical applicability.

*Main text, **Introduction**, line 37-38:*

Several magnetocaloric material families, such as Gd-based systems, Heusler alloys, MM'X alloys and $LaFe_{13-x}Si_x$-based systems, have also attracted considerable interest [5].

5. Gottschall, T. *et al*. Making a Cool Choice: The Materials Library of Magnetic Refrigeration. *Adv. Energy Mater*. 9, 1901322 (2019).

*Main text, **Introduction**, line 42-44:*

This challenge has shifted the frontier of materials science toward high-entropy alloys (HEAs), which not only offer a route towards RE-free magnetocaloric materials, but also present potential benefits in terms of mechanical robustness and corrosion/oxidation resistance, thereby further enhancing their practical applicability.

**Comment 3. "I found the treatment of ferrimagnetic solutions in the DFT calculations to be a bit unclear. While the caption of Figure 2 mentions that such cases were excluded from the subsequent analysis, it is not explicitly stated whether this exclusion applies to the entire study (e.g., whether only ferromagnetic solutions were used to train the surrogate model) or if it is limited to the analysis shown in Figures 2a–l. Given the critical role that magnetic order plays in magnetocaloric materials, I strongly recommend clarifying this point in the main text – or even extending the discussion."**

**Response:** We have made clarifications regarding the treatment of ferrimagnetic solutions in the revised manuscript. The exclusion noted in **Figure 2** captions pertains specifically to the subsequent supercell-based analysis and visualisation. In this section, ferrimagnetic solutions were set aside due to the potential for their antiparallel local moments to create anomalies and inconsistencies when comparing site-resolved magnetic moments and structural characteristics across different supercells.

For the machine-learning dataset, all DFT calculations commenced with ferromagnetic magnetic moments, and the final self-consistent solutions were not strictly constrained to retain purely ferromagnetic properties. Given that the surrogate-model labels consist of continuous DFT-derived values, such as magnetic moment, energy, and volume, we opted to include the limited number of ferrimagnetic or weakly magnetic converged results from this process in the dataset, rather than excluding them entirely.

While we conducted a systematic investigation of alternative magnetic initialisations for the database, those results were not incorporated into the machine-learning dataset. For consistency and minimal variability arising from diverse magnetic initialisation approaches, the surrogate model was trained exclusively on the DFT results derived from a single, coherent ferromagnetic-initialised high-throughput workflow. We have revised the main text to clearly articulate this distinction.

The revised **Figure 2** and caption incorporating all reviewers' comments can be referred to our response to *Reviewer 1, comment 6*.

*Main text, **Methods**, line 547-554:*

To maintain a uniform high-throughput computational protocol, all DFT calculations used for constructing the machine-learning dataset were initialised with ferromagnetic magnetic moments. The final self-consistent solutions were not strictly constrained to retain purely ferromagnetic properties, and a limited number of structures converged to ferrimagnetic or weakly magnetic states. Given that the surrogate models were trained to predict continuous DFT-derived properties, including magnetic moment, energy, and volume, rather than magnetic-order classes, these cases were retained in the machine-learning dataset.

**Comment 4. “The manuscript faces the inherent challenge of balancing depth and accessibility. Given the volume of work and information presented, the text is dense, making it difficult for a general audience to engage with the content. I must admit that, at times, the text—and even the analysis of the figures—was not immediately clear to me. While the technical approach, including the SHAP analysis, is justified, it may reduce accessibility for non-expert readers. I believe this can be improved to ensure the article is understandable to a broader audience, enabling them to fully appreciate the results and methodology.”**

**Response:** In the revised manuscript, we have made several changes to streamline the presentation, clarify key methodological points, and provide more guided explanations of the main results. In particular, we have revised the relevant sections on supercell selection and atomic ordering, condensed the benchmarking discussion to better align with the main narrative, and improved the explanation of the SHAP analysis to make the interpretation of the figures more accessible. Specific changes are described in the sub-comments below.

**Comment 4.1. “In the section titled "Efficient DFT Methodology for HEAs: Supercell Selection and Atomic Ordering," integrating the first paragraph of the supplement could improve clarity. Additionally, providing a brief description (e.g., the number of atoms per cell) or an illustration of the primitive cells would be helpful. It might also be useful to include a note—even if it can be inferred—that the 1×1×1 "supercell" could not accommodate all the compositions examined in the study.**

**Response:** We have incorporated additional explanation to the section “Efficient DFT Methodology for HEAs: Supercell Selection and Atomic Ordering” in the main text of the revised manuscript from the first paragraph of the *Supplementary Information*, including the composition grid, the family of supercells considered, and an explicit note that the 1×1×1 supercell could not accommodate all compositions examined in this work:

*Main text, line 139-143:*

For each series, compositions from $x = 0$ to 1 were sampled in steps of 0.125, using a family of compact supercells (1×1×1, 2×1×1, 1×2×1, 1×1×2, 2×2×1, 1×2×2, 2×1×2). Notably, the 1×1×1 supercell could not accommodate all compositions considered in this work, which motivated the use of larger supercells where required.

**Comment 4.2. “I believe the discussion of the Random Forest algorithm, its comparison with XGBoost, and the performance results (lines 237–283, including Figures 3b and 3c) deviates slightly from the manuscript’s main narrative. These details could be moved to the supplement, which would help streamline the main text and reduce its technical density.”**

**Response:** We have simplified and shortened this discussion, retaining only the essential information needed to justify the choice of XGBoost. Further details on analysis of the Random Forest, XGBoost and stacking ensemble comparison have been moved to *Supplementary Information*. We retain **Figures 3b** and **3c** in the main text to visually summarise the results from the model selection.

*Main text, line 259-261:*

A stacking ensemble [59] was also tested (See **Figure 3c**), but it did not yield meaningful improvement over XGB, which was therefore retained for all subsequent analyses.

**Comment 4.3. "In the discussion of the SHAP analysis results (Figures 4d–h), a more guided explanation of how to interpret these results and their significance would greatly benefit the reader."**

**Response:** Following the reviewer's recommendations, we have revised the related discussion to provide a more guided explanation of how the SHAP panels in **Figures 4d–h** should be interpreted and how they connect to the resulting physical design rules. Specifically, we now clarify that **Figure 4d** provides a global ranking of descriptor importance across the key targets, whereas the subsequent SHAP summary plots (**Figures 4e–f**) and dependence plots (**Figures 4g–h**) offer a more detailed, target-specific view of how the dominant descriptors influence the predictions.

*Main text, line 366-368:*

The global SHAP feature importance plot in **Figure 4d** cuts through this complexity by providing a global ranking of the descriptors across the four key targets, thereby highlighting which classes of features dominate the overall model response.

*Main text, line 378-383:*

To examine this global picture in more detail, the SHAP results can be interpreted at two complementary levels: the summary plots identify the most important descriptors for each target, while the corresponding dependence plots reveal how these descriptors impact the predictions. Following this logic, **Figure 4e** and **Figure 4g** describe the behaviour of $m_{\mathrm{ort}}$, whereas **Figure 4f** and **Figure 4h** highlight the related trends for $\Delta V_{\mathrm{ratio}}$.

**Comment 5. "Why the results for the MnNi-FeSi series, using the 1x1x1 "supercell", has such dispersion in comparison with the other results in in Figure 2.g-l ?"**

**Response:** The MnNi–FeSi series displays a relatively larger dispersion in the energy-related panels for the 1×1×1 representation. We interpret this behaviour as a configuration-dependent sensitivity of the minimal cell to local ordered arrangements, rather than a sign of numerical instability. Such variations, influenced by different ordered atomic configurations within a finite supercell, are quite common in first-principles calculations of substitutional alloys, particularly regarding total or relative energies, as they can reflect different local chemical environments [1]. This interpretation is further supported by the consistency observed in the corresponding volume and magnetization results, which demonstrate stable relaxed structures and magnetic solutions. Additionally, utilizing larger supercells or selecting lowest-energy ordered variant for each composition leads to more coherent compositional trends. Therefore, this observed dispersion does not undermine the overall conclusion that minimal supercells effectively capture the main compositional trends. Instead, it highlights the importance of explicitly evaluating supercell size and configuration effects when applying the smallest-cell model. Although the MnNi–FeSi series appears to have more scatter in **Figure 2g–i**, it is important to note that the absolute energy variation remains around 0.1 eV, which does not significantly alter the qualitative trend of the comparison.

[1] Rowlands, D. A., Ernst, A., Györffy, B. L. & Staunton, J. B. Density functional theory for disordered alloys with short-range order: Systematic inclusion of charge-correlation effects. *Phys. Rev. B* 73, 165122 (2006).

**Comment 6. "Does the surrogate model's predicted total energy have sufficient accuracy (within the established 1–2 meV/atom uncertainty) to reliably compare the orthorhombic and hexagonal phases and allow to identify compositions where the hexagonal phase is the ground state and excluding them as candidates for magnetostructural transitions. It would be insightful to include a confusion matrix in the supplement to address this question."**

**Response:** The accuracy of the predicted total energies play a crucial role when evaluating the orthorhombic and hexagonal phases, especiallly given the typical uncertainty range of 1–2 meV atom$^{-1}$. It is important to clarify that, in this work, the surrogate model is designed not to deliver definitive ground-state classifications at this precision level. Instead, the ML-predicted total energies are used to explore compositions that might exhibit a hexagonal ground state, while also allowing for the possibility of identifying candidates for magnetostructural transitions.

Key to this analysis is the orthorhombic–hexagonal energy difference, $\Delta E_{\mathrm{str}}$, which serves as a valuable descriptor of composition-dependent trends in relative structural stability across the compositional space. Moreover, $\Delta E_{\mathrm{str}}$ is significant as it connects to the structural transition temperature, a notion supported by prior research on magnetocaloric materials (e.g., MM'X alloys) that indicates a correlation between the energy difference of competing phases and their transition behaviour [73,74].

From a physical standpoint, the challenge is inherently continuous rather than discrete. As $|\Delta E_{\mathrm{str}}|$ approaches the intrinsic uncertainty inherent in DFT/ML methods, the assignment of a strict ground-state label becomes less clear. This perspective aligns with earlier findings on Heusler alloys and related compounds [2], which suggest that accurately identifying the ground-state structure can be influenced by nuanced electronic, magnetic, and distortion-related factors, in addition to varying atomic arrangements and supercell configurations. Therefore, it is essential to approach small predicted energy differences with caution and not interpret them as definitive phase assignments.

Another aspect to consider is that the existing experimental phase information presents asymmetric constraints rather than complete ground-state labels. In the MM'X-type systems examined here, the orthorhombic phase typically corresponds to the low-temperature phase, while the hexagonal phase aligns with the high-temperature phase. When the low-temperature orthorhombic phase is experimentally observed, it is reasonable to regard it as the relevant stable phase at lower temperatures. However, it is important to note that the mere observation of the high-temperature hexagonal phase does not necessarily confirm it as the ground state at 0 K, as the orthorhombic phase might still emerge at lower temperatures outside of the reported experimental range. Consequently, the available literature does not furnish a complete and unambiguous set of composition-specific 0 K labels required for a confusion-matrix analysis. For these reasons, a confusion matrix, which relies on clearly defined and unambiguous class labels, has not been implemented. Instead, this model is used to examine trneds in composition-dependent stability and their relationship to transition behaviour. The manuscript has been revised to explicitly state that $\Delta E_{\mathrm{str}}$ is used as a descriptor of relative structural stability and transition-related behaviour, rather than as a strict criterion for ground-state classification.

[2] Faleev, S. V.; Ferrante, Y.; Jeong, J.; Samant, M. G.; Jones, B.; Parkin, S. S. P. Origin of the Tetragonal Ground State of Heusler Compounds. *Phys. Rev. Appl.* **2017**, *7*, 034022.

*Main text, line 328-335:*

These targets were chosen because they capture complementary aspects of the magnetocaloric screening problem, namely low- and high-temperature phase stability, phase-resolved magnetic functionality, phase competition, and transformation-related structural response. In particular, $\Delta E_{str}$ is treated here as a continuous descriptor of the energetic competition between the low-temperature orthorhombic phase and the high-temperature hexagonal phase, providing a basis for estimating or rationalising the martensitic transition temperature[73,74], rather than as a strict ground-state classification label.

73. Fortunato, N. M. *et al.* High-Throughput Design of Magnetocaloric Materials for Energy Applications: MM´X alloys. *Adv. Sci.* **10**, 2206772 (2023).
74. Wang, Y. *et al.* Theoretical and Experimental Insights into the Effects of Zn Doping on the Magnetic and Magnetocaloric Properties of MnCoGe. *Chem. Mater.* **32**, 6721–6729 (2020).

**Comment 7. "Could you please elaborate on why the magnetic moment of the hexagonal phase was chosen as the critical target for the magnetocaloric effect, rather than the magnetic moment of the orthorhombic phase? More specifically, why not focus on the difference between the two?"**

**Response:** For the MM'X systems considered here, the desired magnetocaloric response is associated with a magnetostructural transition from a ferromagnetic low-temperature orthorhombic phase to a weakly magnetic or paramagnetic high-temperature hexagonal phase. In the original version, we were assuming that the magnetic moment of the orthorhombic phase ($m_{\mathrm{ort}}$) was consistently large, and the goal was to minimise the contribution from the hexagonal phase. However, upon further review, we acknowledge the feedback from the reviewer regarding the necessity of this assumption and we can consider the variations of $m_{\mathrm{ort}}$ as a direct descriptor of the ferromagnetic contribution to the magnetisation changes across the transition.

Accordingly, we have revised the analysis framework to incorporate both $m_{\mathrm{ort}}$ and $m_{\mathrm{hex}}$ in a new six-target, phase-resolved SHAP analysis, specifically integrating contributions from both the orthorhombic and hexagonal phases ($m_{\mathrm{ort}}$ and $E_f^{\mathrm{ort}}$ added). This updated approach emphasises the significance of $m_{\mathrm{ort}}$ in characterising the ferromagnetic low-temperature phase, while $m_{\mathrm{hex}}$ provides complementary information on the residual magnetic contributions at high temperatures. By adopting this phase-resolved methodology, we can now more effectively compare the responses of the two competing phases relative to mean and dispersion descriptors, avoiding the potential oversimplification associated with a single magnetic descriptor.

We opted out in using the simple difference between the orthorhombic and hexagonal magnetic moments as the primary descriptor. While this approach could appear straightforward, it tends to compress the distinct magnetic states of the two phases into a single scalar quantity. This could lead to ambiguity since the same moment difference might emerge from varied physical circumstances, whether through an enhanced orthorhombic-phase moment, a diminished hexagonal-phase moment, or concurrent changes in both. Consequently, directly using the moment difference would limit the interpretability of the SHAP analysis, making it challenging to

derive phase-specific design rules. Instead, our phase-resolved strategy allows for a thorough examination of both $m_{\text{ort}}$ and $m_{\text{hex}}$.

The corresponding text and SHAP results have been revised accordingly. Importantly, this revision maintains the core conclusions of our work, while enhancing the alignment of our selected magnetic targets with the underlying physical principles.

The revised **Figure 4**, **Figure S6** and **Figure S7**(combining all changes) have been included in our response to *Reviewer 1, comment 6*.

*Main text, line 324-328:*

To extract clear design principles, we focused on six phase-resolved targets relevant to the magnetocaloric effect: the orthorhombic and hexagonal phase formation energies ($E_f^{\text{ort}}$ and $E_f^{\text{hex}}$), the orthorhombic- and hexagonal-phase magnetic moments ($m_{\text{ort}}$ and $m_{\text{hex}}$), the structural energy difference $\Delta E_{str}$, and the relative volume change $\Delta V_{\text{ratio}}$.

*Main text, line 335-341:*

The inclusion of both $m_{\text{ort}}$ and $m_{\text{hex}}$ enables a phase-resolved analysis of magnetic behaviour across the magnetostructural transition. For the MM'X systems considered here, the desired magnetocaloric response is associated with a transition from a ferromagnetic orthorhombic phase to a weakly magnetic or paramagnetic hexagonal phase; therefore, $m_{\text{ort}}$ is particularly important for describing the ferromagnetic contribution to the magnetisation change, while $m_{\text{hex}}$ provides a reference for the residual magnetism of the high-temperature phase.

*Main text, line 378-397:*

To examine this global picture in more detail, the SHAP results can be interpreted at two complementary levels: the summary plots identify the most significant descriptors for each target, while the corresponding dependence plots reveal how these descriptors impact the predictions. In this context, **Figure 4e** and **Figure 4g** describe the behaviour of $m_{\text{ort}}$, whereas **Figure 4f** and **Figure 4h** emphasise the related trends for $\Delta V_{\text{ratio}}$. This detailed analysis uncovers a refined design dichotomy: baseline-like phase-resolved properties are characterised by substantial mean-descriptor contributions and smooth, continuous descriptor responses, whereas magnetostructural transformation drivers exhibit stronger dispersion-controlled and threshold-like behaviour.

For instance, the formation-energy targets $E_f^{\text{ort}}$ and $E_f^{\text{hex}}$ are both dominated by mass- and size-related mean descriptors, such as the mean atomic weight ($\bar{A}_w$) and mean covalent radius ($\bar{A}_{\text{cov}}$) (**Figure S6a, b**), and show highly consistent response trends (**Figure S7a, b**). Similarly, the hexagonal-phase magnetic moment $m_{\text{hex}}$ is governed primarily by the baseline of unfilled total states ($\bar{u}_{\text{T}}$) (**Figure S6c**), with a largely smooth, nearly monotonic response shown in **Figure S7c**. Although $m_{\text{ort}}$ is ranked highest with respect to the chemical dispersion ($MN_{ad}$) (**Figure 4e**), we classify it together with these baseline-like magnetic properties because $\bar{u}_{\text{T}}$ still provides substantial contributions, and also the SHAP dependence to $MN_{ad}$ remains largely smooth and nearly monotonic (**Figure 4g**). This suggests that $m_{\text{ort}}$ behaves as a continuously tunable ferromagnetic baseline rather than a threshold-like transformation driver.

## Comment 8. Minor suggestions for clarity and presentation

**Comment 8.1. "There appears to be a small typo on line 194: "Figure 2j–l" should likely be "Figure 2g–l.""**

**Response:** We have rectified this typo error (corrected to "**Figure 2g–i**" in *main text, lines 184 and 198*) and also checked throughout the revised manuscript.

**Comment 8.2. "The parity plots in Figure 2d–f and Figure 3d–g would benefit from a square aspect ratio between the x- and y-axes for better visual consistency."**

**Response:** We have adjusted the corresponding panels for a square ratio between the x- and y-axes in **Figure 2d–f** and **Figure 3d–g**.

The revised **Figure 2** and **Figure 3** incorporating all reviewers' comments can be referred to our response to *Reviewer 1, comment 6 and Reviewer 1, comment 4*.

**Comment 8.3. "In Figure 2, the colour legend is repeated in subplots a and b. Given that the legend is consistent across all subplots, a single, unified legend above or below the figure could improve cohesion."**

**Response:** In the revised manuscript, we have replaced the repeated legends in **Figure 2** with a single shared unified legend in the revised figure. Please refer to the updated **Figure 2** provided in our response to *Reviewer 1, comment 6*.

**Comment 8.4. "In Figure 1, adding a downward-pointing arrow in the subset illustration would better highlight the flow of the traditional screening process, clarifying how the authors' approach differs. Moreover, the text boxes in Figure 1 could be repositioned, and the blue text colour reconsidered for improved readability."**

**Response:** We have included the downward-pointing arrow in the subset illustration, made revisions to the text and improved the readability in revised **Figure 1**, following the reviewers' comments and suggestions. The revised **Figure 1** (combining all changes) has been included in our response to *Reviewer 1, comment 2*.

**Comment 8.5. "In Figure 3b, the bright yellow legend text is difficult to discern."**

**Response:** The legend styling in **Figure 3b** has been revised to improve readability. Please refer to the updated **Figure 3** provided in *Reviewer 1, comment 6*.

**Comment 8.6. "The Index column and Feature code in Table 3 may not be essential for the main text. Consider moving the current version to the supplement and simplifying the table in the main text."**

**Response:** We have simplified **Table 3** in the revised manuscript by removing the Index column and Feature code column, while the corresponding descriptor-code information remains available in the *Supplementary Information* **Table S1**.

*Main text, **Table 3**, line 349:*

| Physical category | Physical symbol | Description | Physical interpretation |
| --- | --- | --- | --- |
| Electronic / chemical configuration | $MN_{\mathrm{max}}$ | maximum Mendeleev number | Chemical boundary of the alloy system |
| | $MN_{\mathrm{range}}$ | Mendeleev number range | Breadth of chemical diversity |
| | $MN_{ad}$ | avg_dev Mendeleev Number | Chemical dispersion (Driver of $m_{\mathrm{ort}}$) |
| | $MN_{\mathrm{mode}}$ | mode Mendeleev Number | Most frequent chemical species |
| | $\bar{n}_s$ | mean *s* valence electrons | *s*-orbital filling baseline |
| | $n_{d,\ ad}$ | avg_dev *d* valence electrons | *d*-orbital filling disorder (*d*-band) |
| | $n_{v,\ ad}$ | avg_dev total valence electrons | Global valence electrons variance |
| | $u_{d,\ \mathrm{ad}}$ | avg_dev unfilled *d* states | *d*-band hole dispersion |
| | $u_{\mathrm{T},\ \mathrm{min}}$ | min total unfilled states | Lower bound of total unfilled states (Secondary hysteresis control) |
| | $\bar{u}_{\mathrm{T}}$ | mean total unfilled states | Baseline of total unfilled states (Driver of Stability & $m_{\mathrm{hex}}$) |
| | $u_{\mathrm{T},\ ad}$ | avg_dev total unfilled states | Dispersion of total unfilled states (hysteresis-selective knob) |
| | $u_{\mathrm{T},\ \mathrm{mode}}$ | mode total unfilled states | Dominant unfilled states configuration |
| Structural / steric | $\bar{A}_w$ | mean atomic weight | Mass baseline |
| | $A_{w,\ ad}$ | avg_dev atomic weight | Mass dispersion |
| | $r_{\mathrm{cov},\ \mathrm{range}}$ | range covalent radius | Steric size limit spread |
| | $\bar{r}_{\mathrm{cov}}$ | mean covalent radius | Average atomic packing size |
| | $r_{\mathrm{cov},\ \mathrm{ad}}$ | avg_dev covalent radius | Atomic size distortion |
| | $r_{\mathrm{cov},\ \mathrm{mode}}$ | mode covalent radius | Dominant atomic size |
| Thermodynamic | $T_{\mathrm{melt},\ \mathrm{range}}$ | range melting temperature | Cohesive energy spread |
| | $\bar{T}_{\mathrm{melt}}$ | mean melting temperature | Cohesive energy baseline |
| | $T_{\mathrm{melt},\ \mathrm{mode}}$ | mode melting temperature | Dominant cohesive species |
| Magnetic | $\sigma_{\mathrm{MST}}$ | avg_dev magnetic moment (per atom, 0 K) | Magnetostructural Coupling (The "Trade-off" Driver) |

# Response Letter to Reviewers

We thank the Editor and the Reviewers for their constructive comments. We have revised the manuscript accordingly, and all changes are highlighted in yellow in the revised manuscript. Below, we provide a point-by-point response to each comment.

## Reviewer #3

**The authors have addressed the reviewers' comments and suggestions, implementing changes that streamline the article and expand its reach. They have satisfactorily answered the questions raised and expanded the text so that readers with similar questions can find the answers directly. Additionally, the authors have broadened their discussion on the analysis of both structural phases, completing their study and aligning it with common practices in the field of magnetocaloric materials.**

**Overall, I believe the article will be of great interest to the scientific community, particularly in the caloric materials field, and is ready for publication.**

**Comment 1. "Figure 2: While it is true that the structures are not symmetrically equivalent and may show different trends, the main text groups the supercells into 2x and 4x categories. To streamline the figure (while retaining the current version in the supplement), the authors could group the data for the 1×1×2, 1×2×1, and 2×1×1 supercells under "2x supercell," and similarly for the 4x case."**

**Response:** We have revised **Figure 2** to streamline the main presentation by grouping the supercells into 1×, 2× and 4× categories, consistent with the discussion in the main text. The detailed supercell-resolved version of the figure has been retained in **Supplementary Figure 1**.

**Figure 2:**

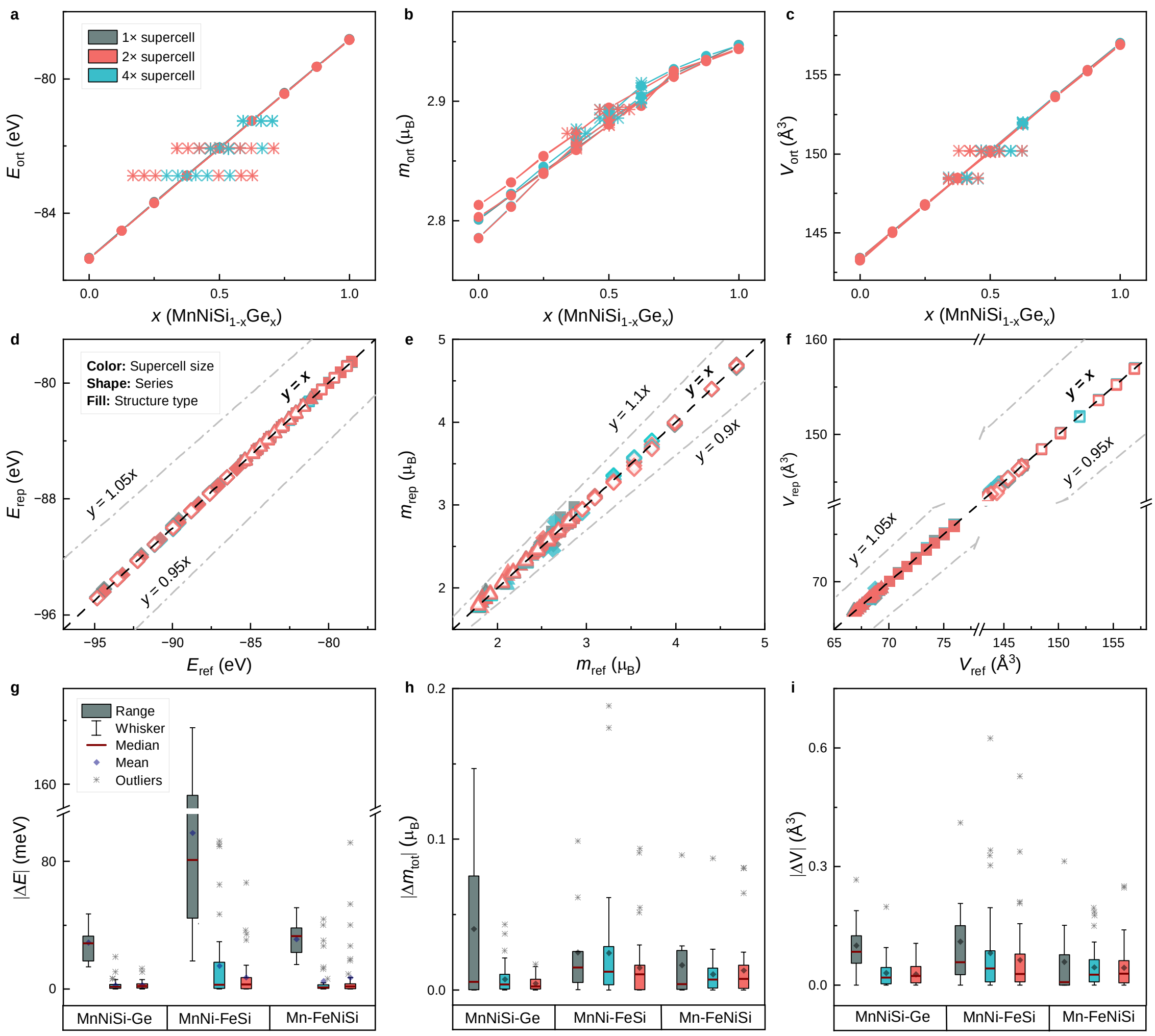


Figure 2. Composition–property relationships and supercell consistency. a, Compositional dependence of energy, *E*, b, magnetic moment, *m*, and c, volume, *V*, for the orthorhombic structure in $MnNiSi_{1-x}Ge_x$. Colours denote supercell size throughout; solid lines connect representative configurations, while symbols indicate non-representative configurations at the same x; a small horizontal displacement is added solely for visual separation. d–f, Parity plots comparing representative values ($P_{rep}$) against the per-composition reference values ($P_{ref}$) for energy, magnetic moment, and volume. The tight clustering along the 1:1 line confirms that minimal supercells introduce negligible systematic bias. Here, symbol shapes distinguish substitution series (squares, MnNiSi-Ge; diamonds, MnNi-FeSi; upward triangles, Mn-FeNiSi) and filled/open symbols distinguish hexagonal and orthorhombic structures. g–i, Box plots summarising the deviation magnitude, $|\Delta P| = |P_{rep} - P_{ref}|$, across all series, demonstrating that property predictions are robust against supercell choice. Boxes show interquartile range (IQR), centre line is the median, whiskers are 1.5×IQR, and outliers are represented by scatter points. Exact n values and underlying data for each box-plot group are provided in Source Data. Detailed residual plots and full error-distribution analyses are provided in Supplementary Fig. 1.

**Supplementary Figure 1:**

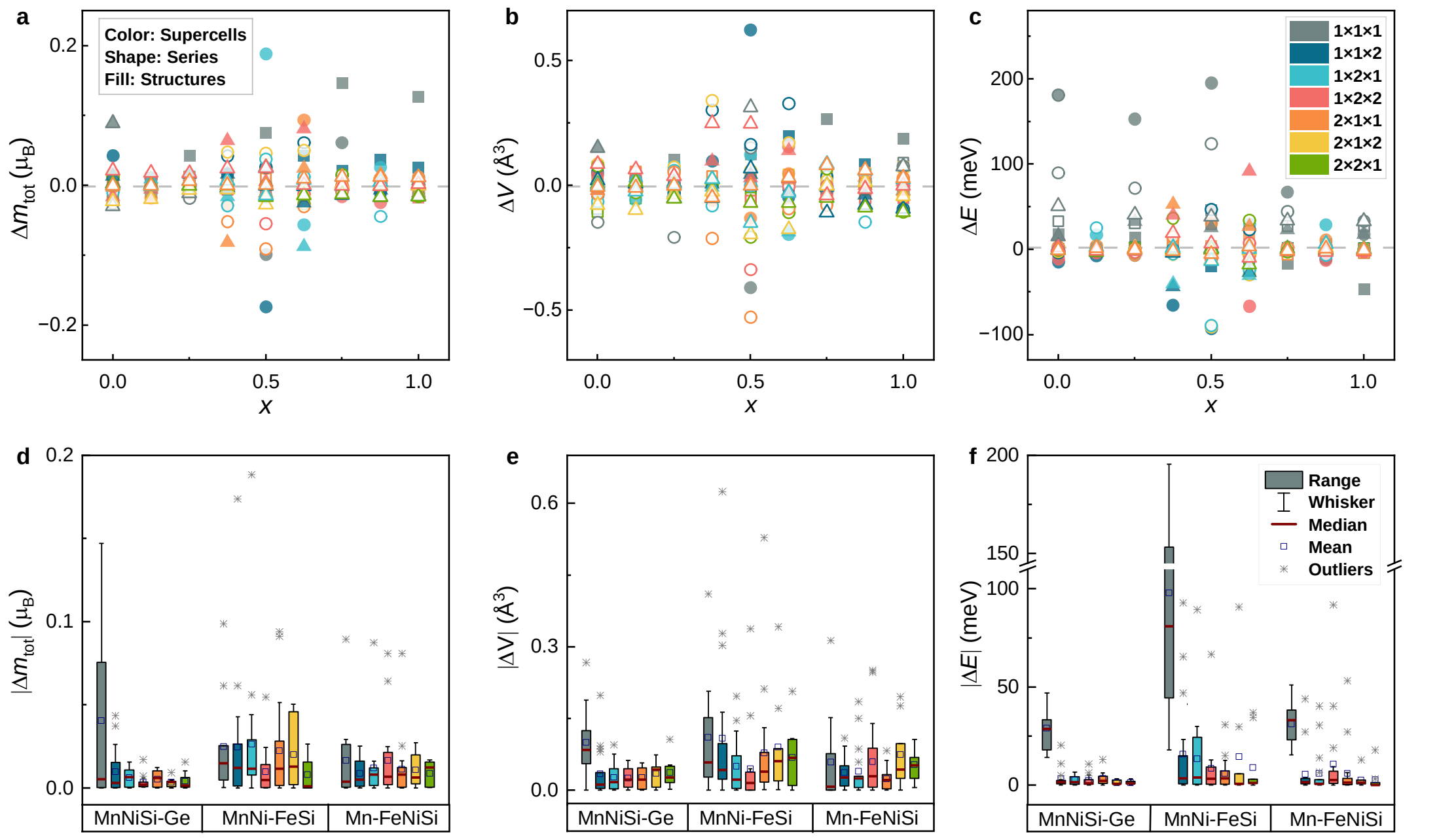


Supplementary Figure 1. Detailed residual and supercell-resolved deviation distributions supporting Fig. 2. Top panels show residuals $|\Delta P| = |P_{rep} - P_{ref}|$ as a function of composition x for magnetic moment, volume and energy. Bottom panels show the corresponding absolute deviation magnitudes, |ΔP|, grouped by substitution series and supercell size. Colours denote supercell size, symbol shapes denote substitution series, and filled/open symbols distinguish crystal structures. Boxes indicate the interquartile range (IQR), centre lines indicate medians, whiskers extend to 1.5×IQR, and points outside the whiskers indicate outliers. For each box-plot group, n denotes the number of independent computational entries used to calculate the distribution, with exact values provided in the Source Data.

**Comment 2. "Line 163 (page 5): For readers unfamiliar with these structures, it would be helpful to specify the corresponding supercell size, e.g., change "(24 atoms)" to "(24 atoms, 2x supercell)" or provide this information earlier, such as: "supercells (1×1×1 (12 atoms), 2×1×1, 1×2×1, 1×1×2 (24 atoms), 2×2×1, 1×2×2, 2×1×2 (48 atoms))".**

**Response:** We have revised the relevant sentence to explicitly identify the 24-atom cell as the 2× supercell.

*Main text, line 122-124:*

For each series, compositions from $x = 0$ to 1 were sampled in steps of 0.125, using a family of compact supercells (1×1×1 (12 atoms), 2×1×1, 1×2×1, 1×1×2 (24 atoms), 2×2×1, 1×2×2, 2×1×2 (48 atoms)).

*Main text, line 150-152:*

Consequently, we establish that the smallest symmetry-compatible supercell (24 atoms, 2× supercell) is sufficient to capture the essential physics, providing a computationally efficient yet rigorous basis for global exploration.

*Main text, **Methods**, line 455-457:*

A family of supercells (1×1×1 (12 atoms), 2×1×1, 1×2×1, 1×1×2 (24 atoms), 2×2×1, 1×2×2, 2×1×2 (48 atoms)) generated by Phonopy 80 were used to realise partial substitutions;

### Comment 3. Table 3: For consistency, consider using the full name for "avg_dev."

**Response:** We have revised **Table 3** as suggested. The abbreviation "avg_dev" has been replaced with its full name, "average deviation," to improve consistency and readability.

***Table 3:***

Table 3. Physicochemical descriptors retained in the final surrogate. The 22 features selected from the original set of 132 Magpie descriptors are grouped by physical category and mapped to the symbolic notation used throughout the text. To facilitate physical interpretation, descriptors are classified into two types: mean descriptor ($\bar{x}$), representing the compositional baseline, and dispersion descriptor ($x_{ad}$), representing the local compositional disorder (calculated as the average absolute deviation). For magnetic properties, $m$ denotes the magnetic moment, with $\sigma_{\mathrm{MST}}$ ($F_{21\_5}$) serving as the critical descriptor for magnetostructural coupling.

| Physical category | Physical symbol | Description | Physical interpretation |
|---|---|---|---|
| Electronic / chemical configuration | $MN_{\mathrm{max}}$ | maximum Mendeleev number | Chemical boundary of the alloy system |
| | $MN_{\mathrm{range}}$ | Mendeleev number range | Breadth of chemical diversity |
| | $MN_{ad}$ | average deviation of Mendeleev Number | Chemical dispersion (Driver of $m_{\mathrm{ort}}$) |
| | $MN_{\mathrm{mode}}$ | mode Mendeleev Number | Most frequent chemical species |
| | $\bar{n}_s$ | mean *s* valence electrons | *s*-orbital filling baseline |
| | $n_{d,\ ad}$ | average deviation of *d* valence electrons | *d*-orbital filling disorder (*d*-band) |
| | $n_{v,\ ad}$ | average deviation of total valence electrons | Global valence electrons variance |
| | $u_{d,\ \mathrm{ad}}$ | average deviation of unfilled *d* states | *d*-band hole dispersion |
| | $u_{\mathrm{T},\ \mathrm{min}}$ | minimum total unfilled states | Lower bound of total unfilled states (Secondary hysteresis control) |
| | $\bar{u}_{\mathrm{T}}$ | mean total unfilled states | Baseline of total unfilled states (Driver of Stability & $m_{\mathrm{hex}}$) |
| | $u_{\mathrm{T},\ ad}$ | average deviation of total unfilled states | Dispersion of total unfilled states (hysteresis-selective knob) |
| | $u_{\mathrm{T},\ \mathrm{mode}}$ | mode total unfilled states | Dominant unfilled states configuration |
| Structural / steric | $\bar{A}_w$ | mean atomic weight | Mass baseline |
| | $A_{w,\ ad}$ | average deviation of atomic weight | Mass dispersion |
| | $r_{\mathrm{cov},\ \mathrm{range}}$ | covalent radius range | Steric size limit spread |

| | | | |
|---|---|---|---|
| | $\bar{r}_{\mathrm{cov}}$ | mean covalent radius | Average atomic packing size |
| | $r_{\mathrm{cov,\ ad}}$ | average deviation of covalent radius | Atomic size distortion |
| | $r_{\mathrm{cov,\ mode}}$ | mode covalent radius | Dominant atomic size |
| Thermodynamic | $T_{\mathrm{melt,\ range}}$ | melting temperature range | Cohesive energy spread |
| | $\bar{T}_{\mathrm{melt}}$ | mean melting temperature | Cohesive energy baseline |
| | $T_{\mathrm{melt,\ mode}}$ | mode melting temperature | Dominant cohesive species |
| Magnetic | $\sigma_{\mathrm{MST}}$ | average deviation of magnetic moment (per atom, 0 K) | Magnetostructural Coupling (The trade-off Driver) |

**Comment 4. Line 426 (page 15): Replace "Note:" with "Note that" to improve text flow.**

**Response:** The wording has been revised from "Note:" to "Note that" to improve the flow of the sentence.

*Main text, **Methods**, line 350-352:*

Note that: since the orthorhombic and hexagonal phase formation energies, $E_f^{\mathrm{ort}}$ and $E_f^{\mathrm{hex}}$, share highly consistent drivers and response trends, we report the general formation-energy behaviour $E_f$ (using only $E_f^{\mathrm{hex}}$ as the representative stability metric).

**Comment 6. "In the supplement: Supplementary lines 223 and 225 (page 8): Change "hexagonal" to "orthorhombic."**

**Response:** The two instances have been corrected from "hexagonal" to "orthorhombic" in the revised Supplementary.

***Supplementary Methods**, line 274-277:*

- the hexagonal formation energy $E_{\mathrm{f}}^{\mathrm{hex}}$;
- the orthorhombic formation energy $E_{\mathrm{f}}^{\mathrm{ort}}$;
- the hexagonal magnetic moment $m_{\mathrm{hex}}$;
- the orthorhombic magnetic moment $m_{\mathrm{ort}}$;
